# New genetic and epigenetic insights into the chemokine system: the latest discoveries aiding progression toward precision medicine

**DOI:** 10.1038/s41423-023-01032-x

**Published:** 2023-05-17

**Authors:** Hanli Xu, Shuye Lin, Ziyun Zhou, Duoduo Li, Xiting Zhang, Muhan Yu, Ruoyi Zhao, Yiheng Wang, Junru Qian, Xinyi Li, Bohan Li, Chuhan Wei, Keqiang Chen, Teizo Yoshimura, Ji Ming Wang, Jiaqiang Huang

**Affiliations:** 1 College of Life Sciences and Bioengineering, School of Physical Science and Engineering, Beijing Jiaotong University, 3 ShangyuanCun, Haidian District, 100044 Beijing, P.R. China; 2 Cancer Research Center, Beijing Chest Hospital, Capital Medical University, Beijing Tuberculosis and Thoracic Tumor Institute, 101149 Beijing, China; 3 Laboratory of Cancer Innovation, Center for Cancer Research, National Cancer Institute at Frederick, Frederick, MD 21702 USA

**Keywords:** Chemokine, Chemokine receptor, Migration, Homeostasis, Genetics, Epigenetics, Chemokines, Predictive markers

## Abstract

Over the past thirty years, the importance of chemokines and their seven-transmembrane G protein-coupled receptors (GPCRs) has been increasingly recognized. Chemokine interactions with receptors trigger signaling pathway activity to form a network fundamental to diverse immune processes, including host homeostasis and responses to disease. Genetic and nongenetic regulation of both the expression and structure of chemokines and receptors conveys chemokine functional heterogeneity. Imbalances and defects in the system contribute to the pathogenesis of a variety of diseases, including cancer, immune and inflammatory diseases, and metabolic and neurological disorders, which render the system a focus of studies aiming to discover therapies and important biomarkers. The integrated view of chemokine biology underpinning divergence and plasticity has provided insights into immune dysfunction in disease states, including, among others, coronavirus disease 2019 (COVID-19). In this review, by reporting the latest advances in chemokine biology and results from analyses of a plethora of sequencing-based datasets, we outline recent advances in the understanding of the genetic variations and nongenetic heterogeneity of chemokines and receptors and provide an updated view of their contribution to the pathophysiological network, focusing on chemokine-mediated inflammation and cancer. Clarification of the molecular basis of dynamic chemokine-receptor interactions will help advance the understanding of chemokine biology to achieve precision medicine application in the clinic.

Leukocyte migration is a central component of physiological and pathological responses [1–9]. Chemokines are the largest family of cytokines and have chemotactic activity that is essential for host responses in homeostasis and diseases. Chemokines activate cell-surface G-protein-coupled receptors (GPCRs) to generate a regulatory network and play indispensable roles in many processes in immunobiology [10–17]. Imbalances and defects in this system alter host susceptibility to diseases, including diverse inflammatory disorders, infections and malignancies [17–20]. In this article, we highlight the most recent findings related to chemokines and receptors regarding their genetic variations and nongenetic heterogeneity. Our review provides molecular insights for chemokine biology to realize precision medicine.

## Background

### History

Since the discovery of the human chemokine CXCL8 or IL-8 (CXCL8/IL-8) in the last century [21–23], chemokines have been recognized to exist in a complicated mega system [10–12, 14–17]. The rather short but rich history in the field includes two waves of chemokine identification [11, 23–26]: the first discovery of inflammatory chemokines and receptors that mainly attract neutrophils and macrophages (Mφs) in the early 1990s and the second round of chemokines and receptor discovery after the mid-1990s, which identified those chemokines and receptors related to homeostasis and the trafficking of lymphocytes and dendritic cells (DCs). However, chemokine research was really initiated in 1977 after the discovery of platelet factor 4 (PF4), also called CXCL4, which was the first identified peptide containing a prototypical chemokine structure with uncharacterized chemoattractant activity [24, 26–28]. The discovery that CXCL8 and CCL2 (originally called MCP-1) [21, 22] have chemotactic activity was nevertheless a landmark finding in immunology [23, 24].

Recently, rapid advances in technologies, such as next-generation sequencing (NGS), mass spectrometry and nuclear magnetic resonance (NMR), have created abundant datasets allowing integrative multiomics analysis of chemokines even at single-cell resolution [29–40]. Additionally, increasing divergence of chemokines and their receptors has been revealed at multiple omics levels, likely underlying the functional heterogeneity and regulatory plasticity [20, 29–32, 36, 41–43]. Thus, the focus of chemokine research has been shifting from cell biology to a global perspective in life sciences, academia, and the pharmaceutical industry [37, 44–51]. Unfortunately, despite extensive pharmaceutical research, relatively few drugs are currently approved for clinical treatment [41, 44–47, 52]. An important reason is the undefined molecular basis of multiple chemokine-receptor interactions in various microenvironments [15, 20, 29, 41, 42, 45, 52–54]. Therefore, it is critical to distinguish functionally indispensable relationships from redundant ones by providing an in-depth understanding of chemokine-receptor relationships so that they can be targeted by genetic and nongenetic means. This will allow chemokine-based therapeutics to be more efficiently developed, thus likely generating a third wave of chemokine biology research.

### Cell migration and leukocyte trafficking

#### Cell migration

Migration is not only a hallmark of many normal cells that enables them to participate in diverse physiological processes, such as development, immune responses and host defense [4, 5, 7, 55–58], but is also hijacked by malignant tumor cells for dissemination [4, 6–9, 59, 60]. Notably, four commutative principles to define directed cell migration were recently proposed (e.g., chemotaxis, haptotaxis, durotaxis and topotaxis): signal generation, sensing, transmission and signal execution [1].

#### Chemotaxis and leukocyte trafficking

Chemokines are best known for their chemotactic activity, which enables them to guide cell migration: gradually increasing the concentration gradient will attract cells toward the source of the chemokine, generally the site of inflection or tissue injury. Leukocyte trafficking, homing and recirculation are pivotal to proper immune responses and immunosurveillance. Leukocyte trafficking is also an indispensable process for immune cell maturation and tissue development and homeostasis and is regulated by chemokines in concert with other cytokines and adhesion molecules [2, 4, 6–8, 14]. As a consequence, infectious or other pathological agents disrupt normal leukocyte trafficking, resulting in uncontrolled flux of immune cells through the endothelial lymph nodes and bone marrow [7–9, 17, 19, 59–61]. In addition, neutrophils also move from the sites of injury back to the vasculature by following chemokine gradients in mice. This so-called neutrophil reverse migration may play a dual role in both local damage protection and systemic inflammation spread [62–65].

Understanding the spatiotemporal migration of immune cells is vital for comprehensively understanding the significance of chemokine-receptor activities and will enable more specific utilization of chemokines [1–3, 7]. However, the biological heterogeneity of chemokines may be underestimated by current state-of-the-art tools, such as superresolution tissue-clearing techniques and real-time analyses of migratory behavior [2, 3, 7, 14, 20, 29, 30]. Therefore, determining how chemokines efficiently bind to GPCRs to initiate signaling cascades and direct migration and desensitize chemokine receptors to impede cell motility for self-limitation within the injured tissue microenvironment, which has been reshaped by chemokines and innate cell recruitment, is a challenge.

### Chemokine‒receptor system

#### Chemokines

##### Chemokine subfamilies

During the past 30 years, chemokines have been found to be one of the largest subfamilies of cytokines based on systematic nomenclature analyses (Table 1) [10–12, 14–18, 26, 66]. Chemokines are divided into four groups (CC, CXC, XC, and CX3C). The CXC chemokines are subdivided into two categories based on the presence of a glutamyl acid-lysine-arginine (ELR) motif, which determines the unique functions of the members. For example, ELR-containing CXCLs (e.g., CXCL8) are chemotactic for neutrophils, whereas ELR-negative CXC chemokines (e.g., CXCL13) tend to chemoattract lymphocytes but not neutrophils.

**Table 1:** Overview of human and mouse chemokines

Human				Mouse		
Symbol	Location	Aliases	Receptor(s)	Symbol	Aliases	Receptor(s)
**CC**						
*CCL1*	*17q12*	***I-309***, *TCA3*,*P500*,*SISe*	***CCR8***, *ACKR1*	*Ccl1*	*TCA-3*	*Ccr8*
*CCL2*	*17q12*	***MCP1***, *MCP-1*, *SCYA2*, *MCAF*, *SMC-CF*, *GDCF-2*, *HC11*, *MGC9434*	***CCR2***, *CCR4*, *CCR5*, *ACKR1*, *ACKR2*, *ACKR4*	*Ccl2*	***JE***, *MCP-1*	*Ccr2*, *Ccr4*, *Ackr1*, *Ackr2*
				*Ccl12*	*MCP-5*, *Scya12*	*Ccr2*
*CCL3*	*17q12*	***MIP-1-alpha***, *MIP1A*, *SCY3*, *G0S19-1*, *LD78ALPHA*	***CCR1***, ***CCR2***, *CCR4*, ***CCR5***, *ACKR2*	*Ccl3*	*MIP-1 alpha*	*Ccr1*, *Ccr4*, *Ccr5*, *Ackr2*
*CCL3L1*	*17q12*	***MIP1AP***, *LD78BETA*, *G0S19-2*	***CCR1***, ***CCR3***, ***CCR5***, *ACKR2*			
*CCL3L3*	*17q12*	***LD78BETA***, *MGC12815*	***CCR1***, ***CCR3***, ***CCR5***, *ACKR2*			
*CCL4*	*17q12*	***MIP-1-beta***, *ACT-2*, *AT744.1*	***CCR1***, *CCR3*, *CXCR4*, ***CCR5***, *CCR8*, *ACKR2*	*Ccl4*	*MIP-1 beta*, *AT744.1*, *Act-2*	*CCR1*, *CCR5*
*CCL4L1*	*17q12*	***LAG-1***, *MIP-1-beta*, *AT744.2*	*CCR1*, *CCR5*			
*CCL4L2*	*17q12*	***AT744.2***, *CCL4 L*, *SCYA4 L*	*CCR1*, *CCR5*			
*CCL5*	*17q12*	***RANTES***, *SISd*, *TCP228*, *MGC17164*	***CCR1***, *CCR3*, *CCR4*, ***CCR5***, *ACKR1*, *ACKR2*	*Ccl5*	*Rantes*	*Ccr1*, *Ccr3*, *Ccr4*, *Ccr5*
*CCL7*	*17q12*	***MCP-3***, *NC28*, *FIC*, *MARC*, *MCP3*	***CCR1***, *CCR2*, *CCR3*, *CCR5*, *CXCR3*, *ACKR1*, *ACKR2*	*Ccl7*	*MARC*, *FIC*, *MCP-3*	*Ccr1*, *Ccr2*, *Ccr3*
*CCL8*	*17q12*	***MCP-2***, *HC14*	*CCR1*, *CCR2*, *CCR3*, *CCR5*, ***ACKR2***, *ACKR1*, *ACKR4*	*Ccl8*	*MCP-2*, *HC14*, *Scya8*	*Ccr8*, *Ackr1*, *Ackr2*
*CCL11*	*17q12*	***Eotaxin***	***CCR3***, *CCR5*, *ACKR1*, *ACKR2*, *CXCR3*	*Ccl11*	*Eotaxin*	*Ccr3*, *Ackr1*
*CCL13*	*17q12*	***MCP-4***, *NCC-1*, *SCYL1*, *CKb10*	*CCR1*, ***CCR2***, ***CCR3***, *CCR5*, *ACKR2*, *ACKR1*, *ACKR4*			
*CCL14*	*17q12*	***HCC-1***, *HCC-3*, *NCC-2*, *SCYL2 CKb1*, *MCIF*	***CCR1***, ***CCR3***, ***CCR5***, *ACKR1*, *ACKR2*, *ACKR4*			
*CCL15*	*17q12*	***HCC-2***, *NCC-3*, *SCYL3*, *MIP-5*, *LKN-1*, *MIP-1D*, *HMRP-2B*	***CCR1***, ***CCR3***	*Ccl9*	*MIP-1 gamma*, *CCF18*, *MRP-2*	*Ccr1*, *Ccr3*
*CCL16*	*17q12*	***HCC-4***, *SCYL4*, *LEC*, *NCC-4*, *LMC*, *LCC-1*, *CKb12*, *Mtn-1*	*CCR1*, ***CCR2***, *CCR3*, *CCR5*, *CCR8*, *ACKR1*			
*CCL17*	*16q21*	***TARC***, *ABCD-2*	***CCR4***, *CCR8*, *ACKR1*, *ACKR2*	*Ccl17*	*Tarc*, *Abcd-2*	*Ccr4*
*CCL18*	*17q12*	***PARC***, *DC-CK1*, *AMAC-1*, *DCCK1*, *MIP-4*, *CKb7*	***CCR8***, *PITPNM3*, *CCR3*			
*CCL19*	*9p13.3*	*ELC*, ***MIP-3b***,*exodus-3*,*CKb11*	***CCR7***, *ACKR4*, *CCRL2*	*Ccl19*	*MIP-3 beta*, *ELC*, *Exodus-3*	*Ccr7*, *Ackr4*, *CcrL2/LCCR*
*CCL20*	*2q36.3*	*LARC*, ***MIP-3a***, *exodus-1*, *ST38*, *CKb4*	***CCR6***	*Ccl20*	*MIP-3 alpha*, *LARC*, *Exodus-1*,	*Ccr6*
*CCL21*	*9p13.3*	***SLC***, *exodus-2*, *TCA4*, *6Ckine*, *ECL*	***CCR7***, *ACKR4*	*Ccl21a*	*6Ckine*, *Exodus-2*, *SLC*, *TCA-4*, *CK beta 9*	*Ccr7*, *Ackr4*
				*Ccl21b*		*Ccr7*
				*Ccl21d*		*Ccr7*
*CCL22*	*16q21*	***MDC***, *STCP-1*, *ABCD-1*, *DC/B-CK*	***CCR4***, *ACKR2*	*Ccl22*	*ABCD-1*, *MDC*, *DC/beta-CK*	*Ccr4*
*CCL23*	*17q12*	*Ckb-8*, ***MPIF-1***, *MIP-3*, *CKb8*	***CCR1***, *CCR3*, *ACKR2*	*Ccl6*	*C10*, *MRP-1*	*Ccr1*
*CCL24*	*7q11.23*	*Ckb-6*, *MPIF-2*, ***Eotaxin-2***, *MPIF2*	***CCR3***, *ACKR2*	*Ccl24*	*Eotaxin-2*, *MPIF-2*, *CK beta 6*	*Ccr3*
*CCL25*	*19p13.2*	***TECK***, *Ckb15*	***CCR9***, *ACKR4*	*Ccl25*	*TECK*, *CKbeta 15*	*Ccr9*, *Ackr4*
*CCL26*	*7q11.23*	***Eotaxin-3***, *IMAC*, *MIP-4a*	***CCR3***, *CX3CR1*, *CCR2*, *CCR5*	*Ccl26*	*Ccl26 l*, *eotaxin-3*	
*CCL27*	*9p13.3*	***CTACK***, *ALP ILC*, *ESKINE*, *ESKY*, *CTAK*	***CCR10***	*Ccl27a*	*Ccl27*, *CTACK*, *ALP*, *ILC*, *PESKY*, *Eskine*	*Ccr10*
				*Ccl27b*	*Ctack*, *Scya27b*	*Ccr3*
*CCL28*	*5p12*	*SCYA28*, *MEC*, *CCK1*	***CCR3***, *CCR10*	*Ccl28*	*MEC*	*Ccr10*
**CXC**						
*CXCL1*	*4q13.3*	*SCYB1*, ***GROa***, *MGSA-a*, *NAP-3*	***CXCR2***, *ACKR1*	*Cxcl1*	*KC*, *Fsp*, *Gro1*, *GRO-alpha*	*Cxcr2*, *Ackr1*
*CXCL2*	*4q13.3*	*SCYB2*, *GROb*, *MIP-2a*, *MGSA-b*, *CINC-2a*	***CXCR2***, *ACKR1*	*Cxcl2*	*CINC-2a*, *Gro2*, *MIP-2*	*Cxcr2*, *Ackr1*
*CXCL3*	*4q13.3*	*SCYB3*, ***GROg***, *M IP-2b*, *CINC-2b*	***CXCR2***, *ACKR1*	*Cxcl3*	*Dcip1*, *Gm1960*	*Cxcr2*
*PF4*	*4q13.3*	***CXCL4***, *oncostatin-A*, *iroplact*	*CXCR3*, ***CXCR3B***, *ACKR1*	*Pf4*		*Cxcr3*
*PF4V1*	*4q13.3*	***PCXCL1***, *CXCL4V1*, *PF4-ALT*, *PF4A*	*CXCR3*, ***CXCR3B***, *ACKR1*			
*CXCL5*	*4q13.3*	***ENA-78***	***CXCR2***, *CXCR3B*, *ACKR1*	*Cxcl5*	*AMCF-II*, *Cxcl6*, *LIX*, *ENA-78*,	*Cxcr1*, *Cxcr2*, *Ackr1*
*CXCL6*	*4q13.3*	***GCP-2***, *CKA-3*	***CXCR1***, ***CXCR2***, *ACKR1*			
*CXCL7*	*4q13.3*	***PPBP***, ***THBGB1***, ***NAP-2***, *CTAPIII*, *beta-TG*	*CXCR1*, ***CXCR2***, *ACKR1*	*Cxcl7*	*Ppbp*, *NAP-2*, *CTAPIII*, *beta-TG*	*Cxcr1*, *Cxcr2*
*CXCL8*	*4q13.3*	***IL-8***, *SCYB8*, *LUCT*, *LECT*, *MDNCF*, *TSG-1*, *NAP-1*,*3-10* *C*, *MONAP*, *AMCF-I*, *LYNAP*, *NAF*, *b-NAP*, *GCP-1*, *K60*, *GCP1*, *NAP1*	***CXCR1***, ***CXCR2***, *ACKR1*			
*CXCL9*	*4q21.1*	***Mig***, *SCYB9*, *Humig*, *crg-10*	*CXCR1*, *CXCR2*, ***CXCR3***, *ACKR1*, *CCR3*	*Cxcl9*	*MIG*, *CRG-10*	*Cxcr3*
*CXCL10*	*4q21.1*	*IFI10*, *IP-10*, *Crg-2*, *mob-1*,*C7*,*gIP-10*	***CXCR3***, *CCR3*	*Cxcl10*	*CRG-2*, *IP-10*	*Cxcr3*
*CXCL11*	*4q21.1*	*H174*, *b-R1*,***I-TAC***,*IP-9*	***CXCR3***, *CXCR7*, *ACKR1*, *ACKR3*, *CCR3*, *CCR5*	*Cxcl11*	*I-TAC*, *beta-R1*, *H174*, *IP-9*	*Cxcr3*, *Cxcr7*
*CXCL12*	*10q11.21*	*SCYB12*, ***SDF-1***, *SDF-1b*, *PBSF*, *TLSF-a*, *TLSF-b*, *TPAR1*	***CXCR4***, *ACKR2*	*Cxcl12*	*SDF-1*, *PBSF*	*Cxcr4*, *Cxcr7*
*CXCL13*	*4q21.1*	*B****LC***, *BCA-1*, *BLR1 LANGIE*, *ANGIE2*	*CXCR3*, ***CXCR5***, *ACKR1*	*Cxcl13*	*BCA-1*, *BLC*	*Cxcr5*
*CXCL14*	*5q31.1*	***BRAK***, *NJAC*, *bolekine Kec*, *MIP-2 g*, *BMAC*, *KS1*	*CXCR4 [14]*	*Cxcl14*	*BRAK*, *BMAC Bolekine*	*Unknown*
				*Cxcl15*	*Lungkine*, *Weche*	*Unknown*
*CXCL16*	*17p13.2*	*SR-PSOX*, *CXCLG16*, *SRPSOX*	*CXCR6*	*Cxcl16*	*SR-PSOX*	*Cxcr6*
*CXCL17*	*19q13.2*	*Dcip1*, *UNQ473*, *DMC*, *VCC1*	*Unknown*	*Cxcl17*	*DMC*, *VCC-1*	*Unknown*
**XC**						
*XCL1*	*1q24.2*	*Lymphotactin*, *LPTN*, *ATAC*, *SCM-1a*, *SCM-1*	*XCR1*	*Xcl1*	*Lymphotactin*	*Xcr1*
*XCL2*	*1q24.2*	*SCM-1 beta*	*XCR1*			
**CX3C**						
*CX3CL1*	*16q21*	***Fractalkine***, *NTN*, *C3Xkine*, *ABCD-3*, *CXC3C*, *CXC3*, *DMC*, *VCC-1*	*CX3CR1*	*Cx3cl1*	***Fractalkine***, *Neurotactin*	*Cx3cr1*

Official gene names in which all letters are uppercase letters refer to human chemokines (left panel), and official gene names in which the first letter is uppercase and the rest are lowercase refer to murine chemokines (right panel). Alternate names in the ALIASES column shown in BOLD represent the most commonly recognized names. Receptors shown in BOLD are active or main receptor(s). The mouse chemokines homologous to human genes are listed in Table 2 [10–12, 14, 17–19, 46]

##### Chemokine gene orthologs

There are more than 48 human chemokines, with 53 murine counterparts (Table 1). While some chemokines have different names, e.g., murine *Ccl6* and *Ccl9* versus human *CCL15* and *CCL23*, some chemokines are only present in either humans (such as *CXCL8*) or mice (e.g., *Ccl6* and *Ccl12*). Table 2 shows that not all chemokines in humans have exact orthologs in mice. For instance, human *CXCL1* is not homologous to *Cxcl1*, and mouse *Cxcl5* (*LIX*) appears more orthologous to human *CXCL6* (*GCP-2*) than *CXCL5*. Moreover, the numbers of chemokines may not be accurate due to the presence of nonallelic splice variants (SVs) and isoforms. They create considerable genetic and nongenetic heterogeneity, impacting immunosurveillance and susceptibility to a number of diseases. For example, *CXCL4L1*, a nonallelic variant of *CXCL4*, is more angiostatic than *CXCL4* [67] and is found in humans but not in mice. Additionally, three SVs of *Ccl27* (*Ccl27a*, *b*, *c*) are found in mice but not in humans (Table 1). Clarification of orthologous chemokine genes will make it easier to reliably interpret or predict their functionality in mice versus humans [68].

**Table 2:** Orthologous chemokine genes between humans and mice

Orthologous gene pair		Functional information
Human gene	Murine gene	Shared recptor(s)
*CCL1*	*Ccl1*	CCR8
***CCL2***	***Ccl12***	CCR2
*CCL3*	\	\
*CCL3L1*	\	\
***CCL3L3***	***Ccl3***	CCR1; CCR5
*CCL4*	*Ccl4*	CCR1; CCR5
*CCL4L1*	\	\
*CCL4L2*	\	\
*CCL5*	*Ccl5*	CCR1; CCR3; CCR4; CCR5
*CCL7*	\	\
*CCL8*	\	\
*CCL11*	*Ccl11*	CCR3
***CCL13***	***Ccl2***	CCR2; D6
*CCL14*	\	\
*CCL15*	\	\
*CCL16*	\	\
*CCL17*	*Ccl17*	CCR4
*CCL18*	\	\
*CCL19*	*Ccl19*	CCR7; CCR11
*CCL20*	*Ccl20*	CCR6
***CCL21***	***Ccl21a*** ***Ccl21b*** ***Ccl21c***	CCR7; CCR11
*CCL22*	*Ccl22*	CCR4
*CCL23*	\	\
*CCL24*	*Ccl24*	CCR3
*CCL25*	*Ccl25*	CCR9; CCR11
*CCL26*	\	\
***CCL27***	***Ccl27b***	CCR10
*CCL28*	*Ccl28*	CCR10
*CXCL1*	\	\
***CXCL2***	***Cxcl1***	CXCR2
***CXCL3***	***Cxcl2***	CXCR2
*PF4*	*Pf4*	CXCR3
*PF4V1*	\	\
*CXCL5*	\	\
***CXCL6***	***Cxcl5***	CXCR1; CXCR2
*CXCL7*	\	\
*CXCL8*	\	\
*CXCL9*	\	\
*CXCL10*	*Cxcl10*	CXCR3
*CXCL11*	\	\
*CXCL12*	*Cxcl12*	CXCR4; CXCR7
*CXCL13*	*Cxcl13*	CXCR5
*CXCL14*	*Cxcl14*	Unknown
*CXCL16*	*Cxcl16*	CXCR6
*CXCL17*	*Cxcl17*	Unknown
*XCL1*	*Xcl1*	XCR1
*XCL2*	\	\
*CX3CL1*	*Cx3cl1*	CX3CR1

Orthologous chemokine genes between humans and mice were extracted from the NCBI HomoloGene database (https://www.ncbi.nlm.nih.gov/homologene/) via the R package “homologene”, which were mainly based on genetic information. Orthologous chemokine genes pairs with inconsistent names are BOLD. “Shared Receptor(s)” means that both human and murine ligands in the orthologous pair can bind to the same receptor(s), which reflects the functional similarity of homologous genes

##### Characteristic structure of chemokines

**Fig. 1:**
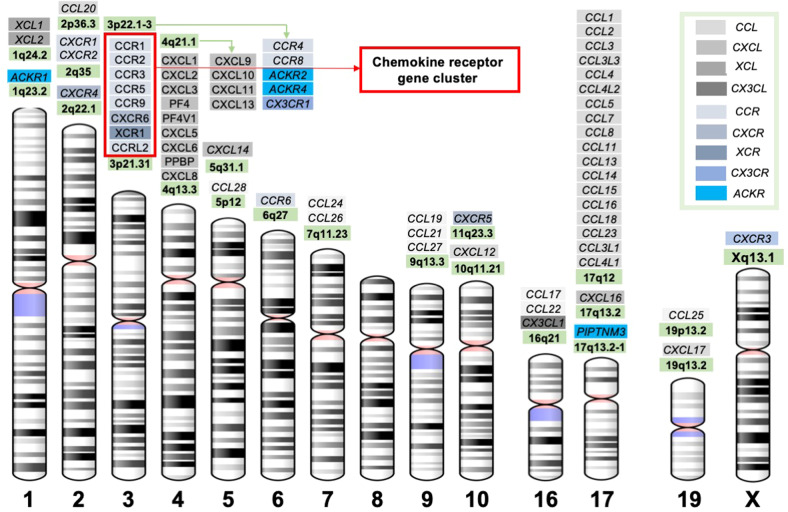
Chromosome location of chemokines and receptors. The locations of chemokines and receptors on human chromosomes. The diagrams of chromosomes were adapted from the NCBI website. The different subclasses of chemokines and receptors are highlighted with different colors

Chemokines are mostly low molecular weight proteins (~8–14 kDa) produced as pro-peptides with a signal peptide that is cleaved to produce active or mature secreted proteins. Most human CXC and CC chemokine-encoding genes are located within clusters on chromosomes 4 and 17, respectively (Table 1 and Fig. 1). Although sequence identity between chemokines varies from approximately 20% to 90%, they are highly conserved overall. The conserved amino acids among chemokines are important for creating their characteristic 3-dimensional and tertiary structures [11, 19, 66, 69]. Some chemokines, such as CCL6, CCL9, CCL23, and CXCR7, contain an extended N-terminus that is proteolytically removed to enhance receptor interaction. Some other chemokines, such as CCL21, contain an extended C-terminus that can also be proteolytically removed to enhance receptor interaction. A few chemokines, such as CX3CL1 (fractalkine) and CXCL16 (SR-PSOX), exist both as cell surface-bound proteins and in soluble forms and elicit immune cell migration and adhesion based on their specific structure (which contains a mucin-like stalk that tethers the chemokine domain to a single transmembrane spanning region). This general structure suggests that chemokine-like factor 1 (CKLF-1) is a novel cytokine, and its chemoattractant capacity is crucial for neutrophils, monocytes and lymphocytes in immune and inflammatory responses [70].

#### Chemokine receptors (CKRs)

CKRs share the seven-transmembrane GPCR architecture that mediates chemotactic signaling. Given that over one-third of clinical drugs function through GPCRs, dissecting the structure–function relationship of GPCRs that contributes to the differences in chemotactic regulatory pathways and mechanisms is crucial for better understanding human physiology and disease etiology and for rational chemokine drug design [37, 38, 44, 45, 47–52].

Chemokines exert their biological activities by interacting with two types of receptors (Table 3). The first so-called classical or conventional chemokine receptors (cCKRs) are a family of Gα_i_-protein-coupled GPCRs including 10 CCRs for CC chemokines, 6 CXCRs for CXC chemokines, XCR1 for XCL1 and XCL2, and CX3CR1 for CX3CL1 [11, 16, 18, 19, 46, 69]. Chemokines binding GPCRs typically trigger the pertussis toxin-sensitive Gα_i_ G-protein signaling pathway. The second receptor group consists of atypical chemokine receptors (ACKRs), which include six members: ACKR1-4, CCRL2 (ACKR5) and PITPNM3 (ACKR6/NIR1) [12, 19, 71]. ACKRs are also seven-transmembrane receptors that mostly couple with β-arrestins to exert diverse roles. ACKRs apparently act as chemokine scavengers or decoy receptors to negatively regulate immune responses.

**Table 3:** The definitive nomenclature of chemokine receptors

Symbol	Locus	Previous symbols	Alias symbols
***CC***			
*CCR1*	3p21.31	*SCYAR1*, *CMKBR1*	*CKR-1*, *MIP1aR*, *CD191*
*CCR2*	3p21.31	*CMKBR2*	*CC-CKR-2*, *CKR2*, *MCP-1-R*, *CD192*, *FLJ78302*
*CCR3*	3p21.31	*CMKBR3*	*CC-CKR-3*, *CKR3*, *CD193*
*CCR4*	3p22.3		*CC-CKR-4*, *CMKBR4*, *CKR4*, *k5-5*, *ChemR13*, *CD194*
*CCR5*	3p21.31	*CMKBR5*	*CKR-5*, *CC-CKR-5*, *CKR5*, *CD195*, *IDDM22*
*CCR6*	6q27	*STRL22*	*CKR-L3*, *GPR-CY4*, *CMKBR6*, *GPR29*, *DRY-6*, *DCR2*, *BN-1*, *CD196*
*CCR7*	17q21.2	*CMKBR7*, *EBI1*	*BLR2*, *CDw197*, *CD197*
*CCR8*	3p22.1	*CMKBRL2*, *CMKBR8*	*CY6*, *TER1*, *CKR-L1*, *GPR-CY6*, *CDw198*
*CCR9*	3p21.31	*GPR28*	*GPR-9-6*, *CDw199*
*CCR10*	17q21.2	*GPR2*	
***CXC***			
*CXCR1*	2q35	*CMKAR1*, *IL8RA*	*CKR-1*, *CDw128a*, *CD181*
*CXCR2*	2q35	*IL8RB*	*CMKAR2*, *CD182*
*CXCR3*	Xq13.1	*GPR9*	*CKR-L2*, *CMKAR3*, *IP10-R*, *MigR*, *CD183*
*CXCR4*	2q22.1		*LESTR*, *NPY3R*, *HM89*, *NPYY3R*, *D2S201E*, *fusin*, *HSY3RR*, *NPYR*, *CD184*
*CXCR5*	11q23.3	*BLR1*	*MDR15*, *CD185*
*CXCR6*	3p21.31		*TYMSTR*, *STRL33*, *BONZO*, *CD186*
***XC***			
*XCR1*	3p21.31	*GPR5*, *CCXCR1*	
***CX3C***			
*CX3CR1*	3p22.2	*GPR13*, *CMKBRL1*	*CMKDR1*, *V28*, *CCRL1*
**ACK**			
*ACKR1*	1q23.2	*FY*, *DARC*	*CCBP1*, *GPD*, *Dfy*, *CD234*
*ACKR2*	3p22.1	*CMKBR9*, *CCBP2*	*CCR10*, *D6*, *CCR9*
*ACKR3*	2q37.3	*CMKOR1*, *CXCR7*	*RDC1*, *GPR159*
*ACKR4*	3q22.1	*CCRL1*	*CCR11*, *CCBP2*, *VSHK1*, *CCX-CKR*, *PPR1*
*CCRL2*	3p21.31		*HCR*, *CRAM-B*, *CKRX*, *CRAM-A*, *ACKR5*
*PITPNM3*	17p13.2-p13.1	*CORD5*	*NIR1*, *RDGBA3*, *ACKR6*

Table is modified from references [11, 12, 14, 18, 19]

G protein-mediated signaling and β-arrestin-mediated signaling have generally been considered separate. However, recent findings show direct formation of Gαi:β-arrestin signaling complexes that are distinct from other canonical GPCR signaling complexes, suggesting that G proteins and β-arrestins are cooperative instead of competitive [72, 73].

### Functional characteristics of chemokines and CKRs

#### Subtypes of chemokines and CKRs

**Fig. 2:**
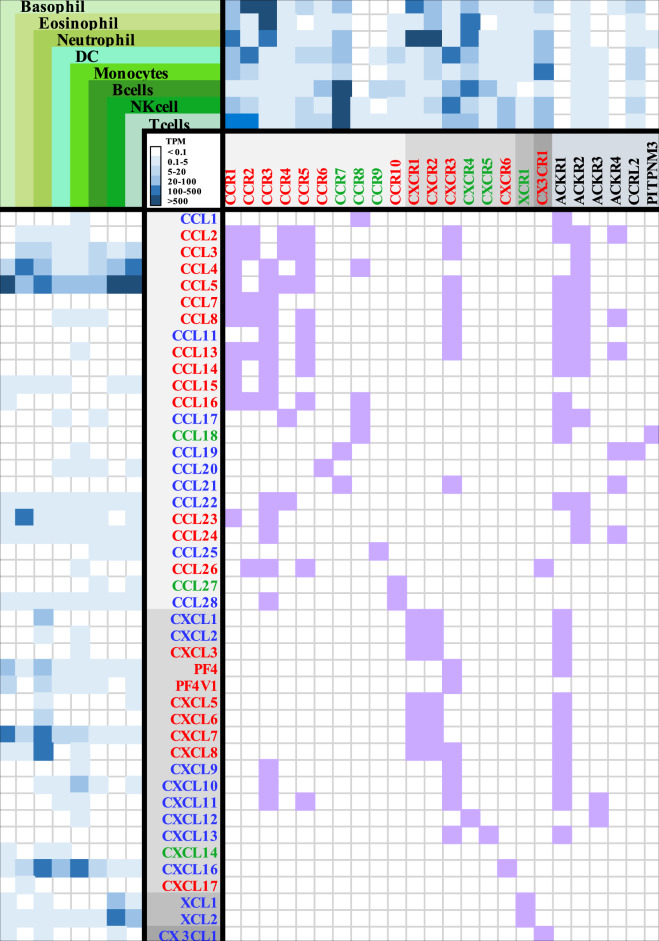
The functional roles mediated by interactions of chemokines with receptors expressed on immune cells. The RNA-seq data were derived from HPA. The relative mRNA expression of chemokines (left hand columns) and receptors (upper right-hand columns) in selected immune cells is shown in the heatmap, with the color based on their transcript per million (TPM) values. The inflammatory and homeostatic chemokines and receptors are shown in red and green, respectively. Chemokines with dual functions are indicated in blue [11, 14, 18, 46]. Chemokine receptors with dual functions are classified into inflammatory families [14]; for example, CCR10/CCL27-CCL28 have been shown to have homeostatic functions [11, 46, 352–354], and several mechanisms have been reported to be involved in inflammation [354]. The atypical chemokine receptors are shown in black. For instance, the platelet chemokine PF4/CXCL4 is quickly released as the first-line inflammatory mediator upon vascular injury and platelet activation. PF4 is also secreted by a variety of immune cells and has also been implicated in the pathology of a variety of inflammatory and autoimmune diseases and cancer [11, 355]. The association of chemokines with receptors was analyzed using STRING (https://string-db.org/), and their interaction networks identified based on the STRING analysis and published reviews [11, 14, 18, 46] are shown in the lower-right hand table, highlighted in purple

Chemokines are classified into homeostatic (or constitutive), inflammatory, and dual function (homeostatic/inflammatory) subtypes based on their expression patterns and functions [11–20, 26, 46, 47]. CKRs are also classified into inflammatory (which control both inflammation and homeostasis) and homeostatic subfamilies [14]. However, accumulated evidence suggests that nonchemokine functions that are also controlled by chemokine ligands and receptors needs to be considered [14, 19, 20, 74]. Homeostatic chemokines and receptors participate in tissue development and basal leukocyte localization, while inflammatory chemokines and receptors regulate immune cell trafficking to sites of inflammation, infection, tissue injury and cancer. The dual subtype chemokines can have either inflammatory and homeostatic activities depending on pathophysiological conditions (Fig. 2) [11, 12, 14, 17, 18, 46].

#### Nomenclature

In general, chemokines with the same name from different species are functional orthologs [11, 66, 75]. Cross-interactions between multiple chemokines and their receptors help to increase the plasticity and specificity of chemotactic functions (Fig. 2). A restricted ligand‒receptor relationship, such as a single receptor interacting with only one or two ligands, is common for chemokines primarily involved in homeostatic cell migration. Thus, the chemokine nomenclature can be helpful for understanding the functional relevance (Table 4) [10–12, 14, 26]. For instance, inflammatory chemokines (e.g., CXCL6, CXCL8, CCL2, CCL3, CCL4, and CCL5) are induced in cells or tissues upon exposure to various stimuli, and their genes are located in clusters (e.g., *CCL* on chromosome 17q12 and *CXCL* on 4q13) (Table 1 and Fig. 1). This is in contrast with the constitutive expression of homeostatic chemokines (e.g., CCL18 and CXCL13) involved in maintaining the migration and positioning of leukocytes in a steady state. Dual chemokines (e.g., CXCL12) are inducible in many tissues in response to inflammatory stimulants and are also constitutively expressed in primary lymphoid tissues. Moreover, knockout of one of the inflammatory chemokines in a cluster often induces less dramatic phenotypes than knockout of individual homeostatic chemokines. Inflammatory chemokine genes, as a product of evolution, are less stable, which may facilitate host survival and evolution [11, 12, 14, 26, 66, 75]. Since chemokines interacting with each other (chemokine interactome) and coupling with different receptors in a complicated crosstalk network can divergently modulate signal transduction [76, 77], understanding the evolution of the chemokine system may make it easier to analyze potential interactions between chemokine receptor pairs underpinning unique biological functions and to discover novel therapeutic targets.

**Table 4:** Logical nomenclature: global insights into the chemokine ligand‒receptor system

Subfamily	Inflammatory	Homeostatic
Location of genes *	Clustered	Isolated
Expression of genes	Conditional upon inflammation	Constitutive
Ligand‒receptor relationship	Multiple ligands for one receptor (e.g., CCL19/CCL21 bind CCR7)	Restrict (one to one)
Chemotactic	Neutrophils (CXC), macrophages, activated lymphocytes	Lymphocytes, dendritic cells, non-activated (homing) lymphocytes
Phenotype (KO)	Alternative	More dramatic
Genomic arrangement (evolution)	Offspring, evolutionary (mutable), dynamic	Oldest, conservative or static
Benefits (Host survival)	Immune responses	Homeostasis and development
Examples	Lack of CCR5 surface expression due to mutation: susceptible to West Nile virus but not HIV	CXCL12: fetal development across various organs

Note: * the detailed information is shown in Tables 1, 2 and Fig. 1

### The expression of chemokines and receptors

#### Bulk expression

Chemokines quantitatively dominate the chemical gradients that recruit cells expressing paired receptors. Therefore, precise assessment of chemokine expression in a spatial-temporal manner is critical for defining their functional properties. As large-scale characterization of sequence-function relations has been achieved, high-throughput, informative data are available for deciphering the normal transcriptomic landscapes of chemokine ligands and receptors. Bioinformatic analysis of these data will provide comprehensive insights into the functional diversity and complexity of the regulatory network of chemokines and receptors (Fig. 2), generating a map of chemokines and receptors that are aligned for “easy indexing” of their expression-function relationship. For example, a CCR6-expressing cell will migrate to a site where the ligand CCL20 is produced, while cells with CCR7 expression may migrate toward a site with increased expression of the ligands CCL19 and CCL21.

#### Single cell-based transcriptomic landscapes

**Fig. 3:**
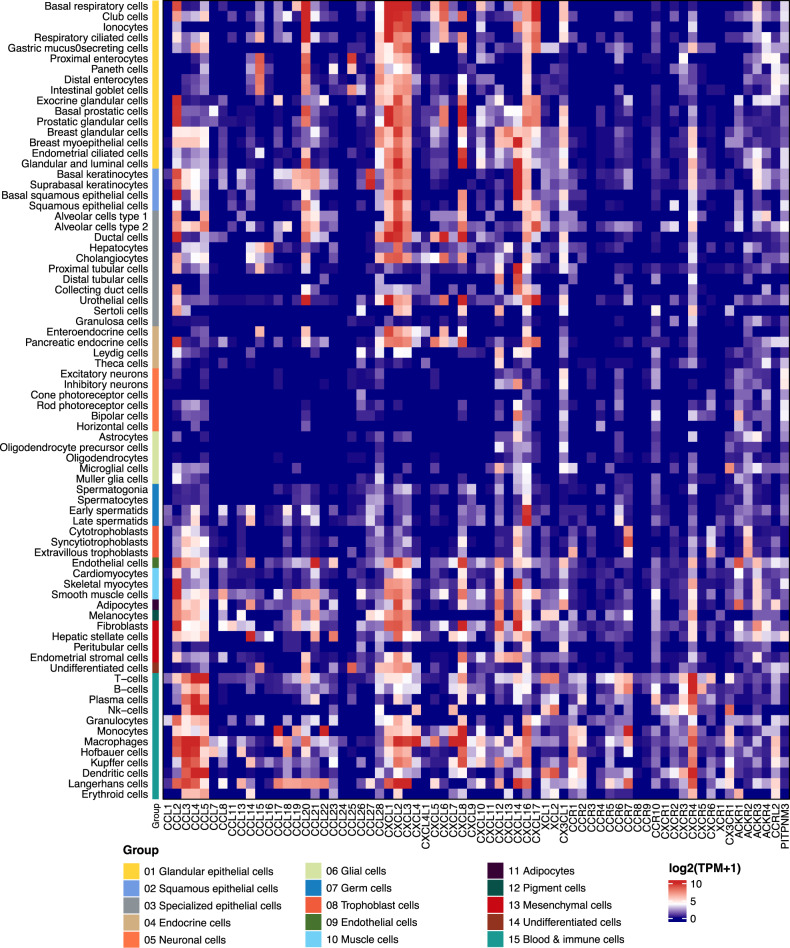
Single-cell expression of chemokines and receptors. A summary of single-cell sequencing analyses of the expression of chemokines and receptors in human tissue cells, including immune cells and total peripheral blood mononuclear cells (PBMCs). Color coding is based on cell type, and each cell type group consists of cell types with common functions. The data were extracted from HPA (https://www.proteinatlas.org/)

The integrative analysis of data from large-scale transcriptome and single-cell RNA sequencing (scRNA-seq) analyses helps to discriminate the transcriptomic heterogeneity and phenotypic divergence of chemokines and receptors underlying their protective and destructive effects [29–36, 78–82]. As shown in Fig. 2, CKR is present on a cell and interacts with one or multiple chemokines to illustrate the complexity of the chemokine network in microenvironment sites, such as, the tumor immune microenvironment (TIME) [19, 41, 45, 83] and inflammatory sites [84], in severe acute respiratory syndrome coronavirus (SARS-CoV-2) infection [35, 36, 85–88]. The landscape heatmap shown in Fig. 3 shows the patterns of chemokine and receptor genes in multiple single cells, including immune cells.

The broad expression of chemokines and CKRs has been thought to be redundant, which may be a reason that targeted drugs have not been successfully developed. For instance, multiple myeloma is a clonal plasma cell proliferative malignancy characterized by an abnormal increase in monoclonal paraprotein in the bone marrow. The application of transcriptome sequencing to reveal single-cell patterns in multiple myeloma patients at different disease stages showed distinct tumor cell populations and microenvironments during disease progression [89]. To recapitulate three populations of natural killer (NK) cells (CXCR4 + , CX3CR1+ and CD56 + ), the CXCR4+ cell-dominated primary NK population is replaced by the CD56+ population during the pretransplant stage. After autologous hematopoietic cell transplantation, the CX3CR1 + NK cell population becomes dominant, and the immune profile remains stable until the first relapse. However, in the second relapse stage, a decrease in the CX3CR1+ population was found to be accompanied by the re-emergence of the CD56 + NK cell population. Such observations highlight the highly dynamic microenvironment during disease initiation and progression, which could not have been unraveled by bulk analysis. However, mounting evidence shows that there is specificity for cell migration and nonredundancy in homeostasis [14, 90].

### Chemokine network

The interactions of multiple chemokines with multiple receptors, and vice versa, are considered a functional axis mediating different signaling events (Fig. 2). The data have illustrated a complex and dynamic chemokine network underlying the regulation of feedback loops, which confers chemotaxis-based cell behaviors in a spatial-temporal manner [1–3, 7, 14–16, 20, 76, 77, 91–94]. Various posttranslation modifications also affect the network to increase its heterogeneity under diverse extracellular and intracellular conditions. For instance, N-terminal or C-terminal truncation of chemokines catalyzed by proteases alters chemokine-receptor interactions, thus influencing the feedback of chemokine networks [16, 91, 95].

The proper migration of immune cells during infection relies on a balance of positive (rapid initiation of protective immunity) and negative (self-shutdown or limitation) feedback from chemokine networks. Compared to bacterial chemotaxis networks, which form negative feedback loops, eukaryotic chemokine networks appear to be “incoherent feedforward loops” [96], representing more complex regulatory networks. The balance between positive migratory cues and negative arrest signals is critical for the directed migration of leukocytes to sites of damage or infection; e.g., T-cell migration in inflamed tissue is shaped by the competition between T-cell receptor (TCR)-induced migratory arrest (‘stop’) and chemokine (‘go’) signals [7].

In addition to well-established positive feedback signaling, recent studies have revealed the mechanisms underlying the formation of chemokine-related “circuit breakers”, e.g., neutrophil swarming and circadian rhythms.

#### Swarming

Neutrophils are the most abundant leukocytes in peripheral blood, and their migratory dynamics in tissues are important for host homeostasis and defense [97–101]. Neutrophils also communicate with each other to be recruited to the site of infection or tissue damage through “swarming” in injured tissues to defend against various invading pathogens. However, an overresponse by neutrophils or other immune cells causes healthy tissue damage and the development of various inflammatory and degenerative diseases [97, 102]. Compared to the well-studied positive feedback loop in immune cell swarming [103], how the autoamplifying responses are eventually turned off to restore the delicate balance between protection and destruction is less clear.

GPCR-mediated negative feedback controls excessive swarm formation based on initial neutrophil activation followed by dynamic arrest in a mouse model. Neutrophils release the mediators Ltb4 and Cxcl2 as well as CAMP/CRAMP to amplify cell swarming and clustering [104, 105]. Neutrophils respond to these high concentrations of swarm mediators by desensitizing the corresponding receptors Ltbr1 and Cxcr2. Desensitization is controlled by the GPCR kinase Grk2 and involves Cxcr2 internalization, whereas desensitized Ltbr1 remains on the plasma membrane of the cells. Grk2 desensitizes Ltb4/Cxcl2-driven signaling pathways in activated neutrophils. Thus, neutrophil aggregation is limited while neutrophil bacteria killing is enhanced, a shutdown mechanism that allows them to deactivate their own receptors that respond to swarm signals [105, 106]. In addition to an interesting finding revealing that B-cell subtypes functionally enriched in the lung microvasculature by CXCL13 and CXCR5 can diminish neutrophil responses [107], another strategy to reduce excessive neutrophil recruitment in inflammatory diseases is targeting downstream regulatory element antagonist modulator (DREAM), a multifunctional transcriptional repressor promoting neutrophil recruitment in vascular inflammation by activating IKKβ and NF-κB and enhancing β2 integrin adhesiveness [108, 109].

#### Circadian rhythms

Leukocyte trafficking around the body and the interstitial migration of immune cells in tissues can be regulated by chemokines and other chemoattractants, and circadian rhythms are essential for all aspects of the relevant biological processes [5, 55–58, 110–112]. The diurnal programming of neutrophils is coordinated by the circadian-related protein Bmal1 (basic helix-loop-helix ARNT like 1, encoded by Arntl)-driven production of CXCL2, which controls neutrophil aging through CXCR2 autocrine signaling [58]; in contrast, Bmal1 coordination with CXCR4, a negative regulator of CXCR2 signaling, results in unrestrained aging. In light of the pervasive effects of circadian time on immune function [57], it is not surprising that targeting the Cxcl12-Cxcr4 axis with G-CSF to mobilize hematopoietic stem and progenitor cells was demonstrated to have more potent effects in mice in the afternoon [113], though the response in humans remains unknown.

Decoding the molecular basis of the chemokine-receptor interactions underlying the regulation of the network architecture will lead to a more comprehensive and precise interpretation of the functional redundancy and specificity of chemokines under various micromovements [20] and resolve other paradoxical aspects of chemokine biology. This may be beneficial for precise therapeutic intervention, e.g., to suppress unwanted inflammation while still enabling appropriate immune responses.

## Genetic and nongenetic alterations of chemokines and receptors

Recently developed analytical techniques and statistical capabilities have enabled integration of multiomics biological information with high-resolution quantitative data of chemokines.

### Genetic variation

#### Disease-associated variants of chemokines and receptors

**Fig. 4:**
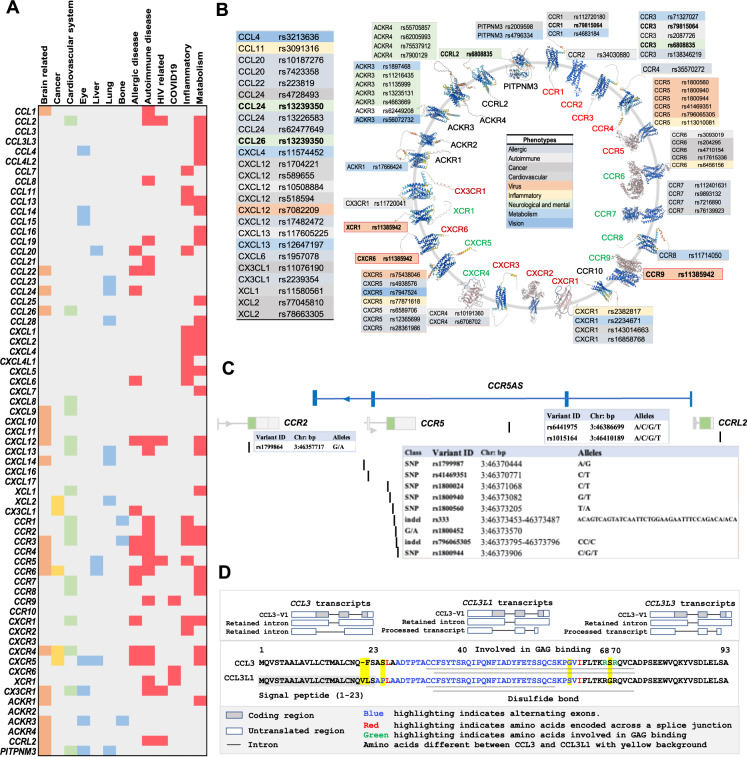
Genetic alterations of chemokine ligands and receptors associated with diseases. **A**, **B**. Clinically relevant single nucleotide variations (SNVs) affecting phenotype, as provided in recently published literature. **A** Chemokine- and receptor-associated SNVs affecting phenotype involved in health and disease. **B** Health- or disease-related SNVs of chemokine genes (left panel) or chemokine receptor genes (right panel) are highlighted with different colors. The predicted three-dimensional (3-D) structure models of the receptors were downloaded from AlphaFold DB (https://alphafold.ebi.ac.uk/). The inflammatory, homeostatic, and dual chemokine receptors are shown in red, blue, and green, respectively. **C** Genetic variations in the CCR5/CCR2 gene cluster at 3p21.31. **D** The structure of the *CCL3L* gene cluster in 17q12, showing common genetic variations. The 17q location contains the genes encoding most of the CCL subfamily members, including CCL1-5, 7, and 8, indicating their functional relevance. *CCL3L*, *CCL3L3* and the pseudogene C-C motif chemokine ligand 3 pseudogene 1 (*CCL3P1*, gene ID: 390788, previous name: *CCL3L2* (upper panels)) are also found in this location. The amino acid alignments and protein domains (lower panels) of *CCL3* (gene ID: 6348), *CCL3L1* (gene ID: 6349), and *CCL3L3* (gene ID: 414062) are shown

The current understanding of the genomic landscape regarding heterogeneity proposes that multiple genomic alterations rather than a single genomic driver should be used in the molecular classification of diseases or as health risk factors [114–116]. The increased availability of transgenic mouse models (Table S1) and human disease-associated genetic data (Table S2) may make it easier to define genetic aberrations related to chemokines as potential standalone targets [54] or combined biomarkers [117–119]. For instance, omics-based approaches such as genome-wide association studies (GWASs) have been applied to detect numerous genetic variants of chemokines and receptors, among which single nucleotide polymorphisms (SNPs) are the main type of aberration associated with susceptibility to diseases, and some have been identified as host genetic risk factors for clinical testing (Table 5, references shown in Table S2). Figure 4A shows the genetic variants of chemokines and receptors, such as single nucleotide variants (SNVs) and deletions (Dels), found in different health conditions and diseases based on the most recent literature (since 2019). These data suggest that many variants of chemokines and receptors are present in metabolic disorders, and the relationship of these variants with immunity has recently been identified [20, 30, 33, 35, 37–39, 42, 43, 120, 121]. Most variants of chemokines and receptors are associated with multiple diseases or disorders, suggesting their contribution to genetic heterogeneity. Figure 4B shows health disorder-associated chemokine or receptor SNVs, some of which have been used in the clinic for standalone or combined tests (Table 5).

**Table 5:** Clinical interpretation of the genomic variants of chemokines and receptors

Gene	Variant(s)	Type	Disease name	CLNSIG	Status	ID
*CXCR4*	NM_003467.3:c.893_1034dup (p.Glu345_Ser346insProHisProLeuCysPheProTrpSerGlnIleTer)	Dup	Warts, hypogammaglobulinemia, infections, and myelokathexis	Likely pathogenic	1 Star	1513755
*CXCR4*	NM_003467.3:c.1025_1028del (p.Thr342fs)	Del	Warts, hypogammaglobulinemia, infections, and myelokathexis	Likely pathogenic	1 Star	1319371
*CXCR4*	NM_003467.3:c.1027 G>T (p.Glu343Ter)	SNV	WHIM syndrome 1	Pathogenic	none	14022
*CXCR4*	NM_003467.3:c.1016_1017del (p.Ser339fs)	Del	Warts, hypogammaglobulinemia, infections, and myelokathexis|WHIM syndrome 1	Likely pathogenic	1 Star	14021
*CXCR4*	NM_003467.3:c.1012_1015dup (p.Ser339fs)	Dup	not provided	Pathogenic	1 Star	1338437
*CXCR4*	NM_003467.3:c.1013 C>G (p.Ser338Ter)	SNV	Warts, hypogammaglobulinemia, infections, and myelokathexis|WHIM syndrome 1|not provided	Pathogenic	2 Stars	14023
*CXCR4*	NM_003467.3:c.1013 C>A (p.Ser338Ter)	SNV	WHIM syndrome 1	Likely pathogenic	1 Star	1685294
*CXCR4*	NM_003467.3:c.1006 G>T (p.Gly336Ter)	SNV	Warts, hypogammaglobulinemia, infections, and myelokathexis	Pathogenic	1 Star	1453229
*CXCR4*	NM_003467.3:c.1003 G>A (p.Gly335Ser)	SNV	Warts, hypogammaglobulinemia, infections, and myelokathexis|not provided	Conflicting interpretations of pathogenicity	1 Star	372600
*CXCR4*	NM_003467.3:c.1000 C>T (p.Arg334Ter)	SNV	Warts, hypogammaglobulinemia, infections, and myelokathexis|WHIM syndrome 1|not provided	Pathogenic	2 Stars	14020
*CXCR4*	NM_003467.3:c.994 G>T (p.Gly332Ter)	SNV	Warts, hypogammaglobulinemia, infections, and myelokathexis	Likely pathogenic	1 Star	574352
*CXCR4*	NM_003467.3:c.988_989del (p.Ser330fs)	MS	not provided	Pathogenic	1 Star	1163801
*CXCR4*	NM_003467.3:c.959_960del (p.Val320fs)	MS	Warts, hypogammaglobulinemia, infections, and myelokathexis	Likely pathogenic	1 Star	1067193
*CXCR4*	NM_003467.3:c.950_953del (p.Leu317fs)	Del	Inherited Immunodeficiency Diseases	Pathogenic	1 Star	827702
*CXCR4*	NM_003467.3:c.952dup (p.Thr318fs)	Dup	not provided	Pathogenic	1 Star	988527
*CXCR4*	NM_003467.3:c.582 G>C (p.Leu194Phe)	SNV	Warts, hypogammaglobulinemia, infections, and myelokathexis|not provided	Conflicting interpretations of pathogenicity	1 Star	624148
*CXCR4*	NM_003467.3:c.219 G>A (p.Thr73 = )	SNV	Warts, hypogammaglobulinemia, infections, and myelokathexis|not specified	Conflicting interpretations of pathogenicity	1 Star	1096326
*CXCR4*	NM_003467.3:c.157 A>C (p.Ile53Leu)	SNV	Warts, hypogammaglobulinemia, infections, and myelokathexis|WHIM syndrome 1|not specified|not provided	Conflicting interpretations of pathogenicity	1 Star	709395
*CXCR4*	NM_003467.3:c.153 T>A (p.Thr51 = )	SNV	Warts, hypogammaglobulinemia, infections, and myelokathexis|not provided	Conflicting interpretations of pathogenicity	1 Star	374560
*CXCR2*	NM_001557.4:c.458 G>A (p.Arg153His)	SNV	not provided	Conflicting interpretations of pathogenicity	1 Star	809151
*CXCR2*	NM_001557.4:c.472 A>T (p.Lys158Ter)	SNV	not provided	Likely pathogenic	1 Star	809152
*CXCR2*	NM_001557.4:c.623 G>A (p.Arg208Gln)	SNV	WHIM syndrome 2	Likely pathogenic	1 Star	1339556
*CXCR2*	NM_001557.4:c.968del (p.His323fs)	Del	WHIM syndrome 2	Pathogenic	none	1177051
*CCR2*	NM_001123396.4:c.190 G>A (p.Val64Ile)	SNV	Susceptibility to HIV infection	Protective	none	8267
*CCR5*	NM_000579.4:c.-301 + 246 A > G	SNV	CCR5 PROMOTER POLYMORPHISM|Susceptibility to HIV infection|Acquired immunodeficiency syndrome, delayed progression to	Conflicting interpretations of pathogenicity; Protective	none	8189
*CCR5*	NM_001394783.1:c.180 G>T (p.Arg60Ser)	SNV	Susceptibility to HIV infection	Protective	none	8191
*CCR5*	NM_001394783.1:c.303 T>A (p.Cys101Ter)	SNV	Susceptibility to HIV infection	Protective	none	8188
*CXCL12*	NM_199168.4:c.*531 G > A	SNV	Susceptibility to HIV infection	Protective	none	8762
*CCL2*	NG_012123.1:g.2493 A > G	SNV	Spina bifida, susceptibility to|Mycobacterium tuberculosis, susceptibility to|Coronary artery disease, modifier of|Coronary artery disease, development of, in hiv	Pathogenic; risk factor	none	14207
*CCL2*	NG_012123.1:g.2936=	SNV	Susceptibility to HIV infection	Protective	none	14205
*CCL2*	NM_002982.4:c.77-109=	SNV	Susceptibility to HIV infection	Protective	none	14206
*CCL11*	NG_012212.1:g.3760=	SNV	Susceptibility to HIV infection	Protective	none	8367
*CCL5*	NM_001278736.2:c.76+231 T > C	SNV	Human immunodeficiency virus type 1, rapid disease progression with infection by	Pathogenic	none	12740
*CCL5*	NG_015990.1:g.4973 C > G	SNV	Human immunodeficiency virus type 1, delayed disease progression with infection by	Pathogenic	none	12739

The clinically significant genetic variants in chemokine genes were searched from the ClinVar database (https://www.ncbi.nlm.nih.gov/clinvar), which is a freely accessible, public archive of reports of the relationships among human variations and phenotypes, with supporting evidence. In the Type column, Dup: duplication; Del: deletion; SNV: single nucleotide variant; and MS: microsatellite. In the Disease Name column, different diseases are separated by “|”. CLNSIG: clinical significance. The column ID ID: clinvar access ID

#### Genetic variants of chemokines and receptors in viral infection

Understanding the genetic basis of the host immune response to viral infection and host resistance will help delineate the plausible genetic determinants of immune diseases and cancer. For example, CCR5 plays an essential role in lymphocyte migration to sites of inflammation and immunosurveillance by binding its natural agonist ligands, including CCL3, CCL3L1, CCL4/MIP-1*β* and CCL5 (Fig. 2). *CCR5*, *CCR2*, *CCR3*, and *CXCR4* are the genes encoding viral coreceptors, and the allelic variants and natural ligands (e.g., *CCL3* transcripts and *CXCL12/SDF-1*) of these genes have been well studied in correlation with natural susceptibility or resistance to human immunodeficiency virus (HIV) infection [122]. Genetic loss-of-function of *CCR5/RANTES* (CCR5-Δ32, a 32-bp natural deletion resulting in a nonfunctional receptor) confers HIV-1 resistance [123, 124], although CCR5-Δ32 was not shown to be a factor protecting against HIV infection in an analysis of ClinVar data (Table 5). CXCR4, a specific receptor for CXCL12/SDF-1, plays an essential role in hematopoiesis and carcinogenesis (Fig. 4A). Mutations in its gene have been associated with WHIM syndrome. CCR5 and CXCR4 are major coreceptors (CD4 being the primary receptor) for HIV to enter host cells, and these genetic variants have been targeted for antiretroviral therapy interruption, attracting R&D interest [125–131].

##### CCR5/CCR2 gene cluster and HIV

The *CCR5/CCR2* gene cluster, which spans 20 kb on chromosome 3p21.31, has been found to be a highly diverse region with many phenotypic SNVs (Fig. 1 and Fig. 4C); thus, *CCR5/CCR2* haplotypes are used for analysis of the association of candidate genes with HIV-1 infection [132, 133]. For instance, *CCR2*-V64I (rs1799864) has an association with certain SNPs (e.g., rs1799987) in the *CCR5* cis-regulatory region (Fig. 4C) and plays a beneficial role during HIV-1 infection [133, 134]. Genotyping of multiple variants (9 in *CCR5/CCR2*, 2 in *CCL3* and 2 in *CCL5*) was performed in HIV-seropositive individuals, and the results showed that specific combinations of variants in genes from the same signaling pathway could define an HIV-1 resistant phenotype [135]. As shown in a longitudinal case-controlled study of 502 adult HIV-positive participants, the circulating concentrations and gene expression patterns of *CXCL12* (rs1801157) and *CCL2* (rs1799864) were associated with immune recovery status; furthermore, strong linkage disequilibrium (LD) between *CCR2* rs1799864 and *CCR5* rs1800024 and between *CCR2* rs1799864 and *CCR5* rs333 determined the baseline plasma CCR2 and CCR5 concentrations in participants with poor immune response. This suggests that dual blockade (CXCL12 and CCL2, CCR2 and CCR5) may be a useful therapeutic strategy for future clinical trials [117]. Further integrated genome and transcriptome analyses of antibody response and viral antigen positivity elucidated novel genetic determinants related to viral infection and the immune response, and *CXCR5* was identified as one of 7 novel genes associated with viral antibody response. This indicates that chemokine genes beyond the human leukocyte antigen (HLA)-class II region not only contribute to host‒virus interactions but dominate the landscape of the viral antibody response [119].

To the SNP rs7082209 affects an area upstream of *CXCL12* and is associated with decreased susceptibility to tuberculosis (TB) in HIV-positive individuals [136]. *CCR5* promoter polymorphisms, including rs2734648 and rs1799987, in the Chinese Han population were shown to confer an extraordinarily increased risk of susceptibility to pulmonary TB and TB progression, possibly because they affect transcription factor-binding sites to regulate *CCR5* expression [137]. Deficiency of the GATA-1 binding site in the *ACKR1/DARC* promoter, which abolishes erythroid gene expression in Duffy-negative individuals, thus conferring resistance to *Plasmodium vivax*, was demonstrated to be the underlying mechanism [138, 139]. Another novel mechanism of an SNP in the regulation of HIV-1 infection was recently uncovered by Kulkarni et al. [140]. The SNP rs1015164A/G maps downstream of *CCR5* (approximately 34 kilobases) and leads to variation in an activating transcription factor 1 (ATF1)-binding site that controls the expression of CCR5AS (Fig. 4C). CCR5AS blocks interactions between the RNA-binding protein Raly and the *CCR5* 3ʹ untranslated region, protecting *CCR5* mRNA from Raly-mediated degradation. Reduced CCR5 expression induced by inhibition of CCR5AS diminished infection of CD4 + T cells with CCR5-tropic HIV, thus influencing HIV disease outcome [140]. Since the genetic factors affecting these chemokines and receptors are located in noncoding regions, such as promoters, enhancers and intergenic regions, their alterations may increase the transcriptional regulatory plasticity of chemokine molecules. This is evidenced by the common super-enhancer (SE) located in the genomic region for *XCR1* and *CCR1*; the SE is near the *CCR1* gene locus and is linked to high transcriptional activity of *CCR1* [141]. Differential polymorphisms occurring at splicing sites may lead to aberrant alternative splicing variants (SVs) with functional divergence and even opposing activities. However, this possibility remains to be further explored.

##### The CCL3/CCL3L1-CCR5 axis in HIV

CCL3 is a natural ligand for the HIV-1 coreceptor CCR5, colocalizing with *CCL3L3* and the pseudogene C-C motif chemokine ligand 3 pseudogene 1 (*CCL3P1*) in a region of human 17q12 containing most of the CCL chemokines (Fig. 1, Fig. 4D), indicating their functional relevance. *CCL3* has three SVs, but only *CCL3-V1* encodes the 92-aa chemokine CCL3. *CCL3L1 (SCYA3 L/MIP1A)* in the 17q12 alternate locus shares ~96% nucleotide sequence identity with *CCL3* and encodes a 93-amino acid preprotein with differences in several key amino acid residues. *CCL3L3* is a centromeric copy of *CCL3L1* with identical amino acids (Fig. 4D).

The affinity of CCL3L1 binding to CCR5 was much higher than that to CCL3 and CCL5, and CCL3L1 is the most potent agonist of CCR5 and suppresses HIV-1 infection [142, 143], whereas CCL7/MCP-3 is the main antagonistic ligand of CCR5. The inhibitory effect of CCL3L1 on the entry of HIV-1 into CCR5-expressing cells is due to the proline (P) that is visible in position 2 of mature CCL3L1 (after removal of the signaling peptide). Moreover, individuals tend to have distinct copy number variations (CNVs) of *CCL3L1*, whereas there is typically only a single copy of *CCL3* per haploid genome. Thus, CCL3L1 may be a dominant HIV-suppressive chemokine. Generic variants such as CNVs of *CCL3L1* have been implicated in HIV-1 susceptibility [144]. Interestingly, *CCL3* antisense RNA 1 (*CCL3-AS1*) has several SVs and was found to map near *CCL3* in 17q12, with yet to be clarified patterns of expression and function.

##### Chemokine variants in COVID-19

**Fig. 5:**
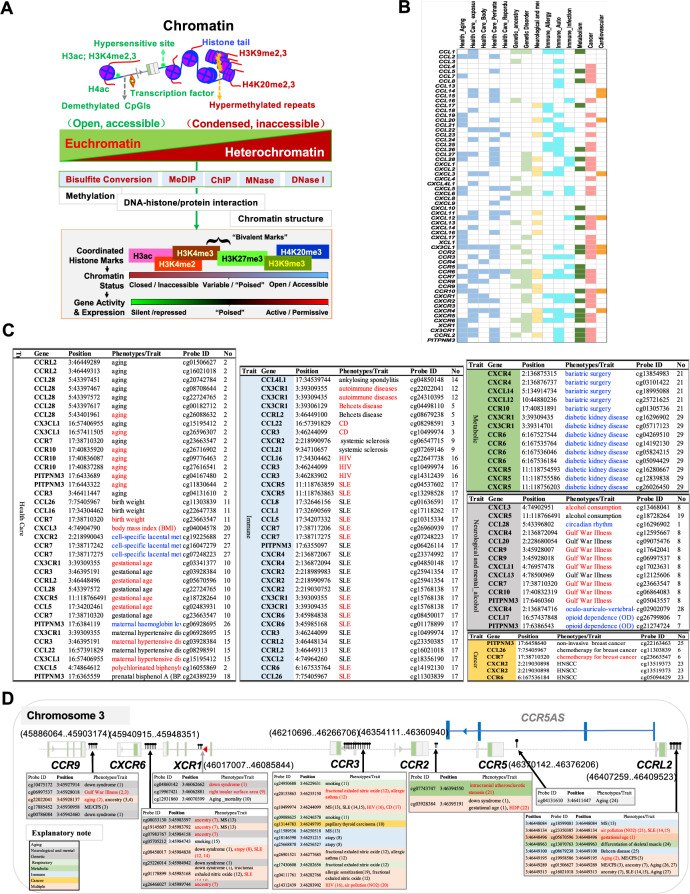
Regulatory chromatin markers and health- and disease-associated CpG methylation. **A** Chromatin in nondividing cells can be divided into euchromatin and heterochromatin, and the two chromatin states refer to areas that are transcriptionally active and inactive, respectively. Epigenetic factors include DNA/RNA methylation and histone modifications, RNA transcript variations (e.g., different splice forms of RNA as epigenetic regulators), and noncoding RNAs (ncRNAs, such as miRNAs, sRNAs, and ncRNAs as well as RNAi and AS), as well as chromatin architecture remodeling [150, 157–166]. Covalent epigenetic modifications of histones and DNA are the most common epigenetic marks, and they alter neighboring nucleosomes to impact the accessibility of loci for transcription factors and coregulators. The gene or regulatory element associated with these epigenetic modification marks indicates the status (active, repressive or poised). These epigenetic marks can be determined using epigenetic analyses. Examples include chromatin immunoprecipitation (ChIP), micrococcal nuclease (MNase) and DNase I hypernasality site (DHS) assays with PCR or sequencing techniques [152, 161, 162, 169–174]. **B** Heatmap showing the differentially methylated chemokine genes associated with health and disease. **C** Chemokine genes with differential CpG methylation associated with normal processes such as aging, body weight control, immune responses, metabolism and diseases such as neurological and mental disorders. **D** Health- and disease-associated CpG methylation is found in the CCR5/CCR2 gene cluster

An understanding of the genetic and immunological determinants of resistance to infection (e.g., autosomal recessive deficiencies of CCR5 in HIV-1 infection and deficiency of ACKR1 in *Plasmodium vivax* infection) may provide a road map for identifying monogenic or common determinants of resistance or susceptibility to infection with SARS-CoV-2 [54, 118]. In addition to a suggestive association between *CCL2*-A2518G gene variants and the severity of COVID-19 [145], a genome-wide study showed associations between the risk of severe COVID-19 and a multigene locus at 3p21.31 and the *ABO* blood group locus at 9q34.2. Regarding the locus at 3p21.31, the frequency of the rs11385942 insertion–deletion GA or G variant is related to predisposition to the most severe forms of COVID-19; and the gene cluster including *CCR9*, *CXCR6* and *XCR1* (Fig. 5D) is involved in T-cell and dendritic cell function. The identified 3p21.31 (CCR5/CCR2) gene cluster may thereby act as a genetic biomarker for susceptibility to COVID-19 infection [146]. Exploring the effect of chemokine gene variants on SARS-CoV-2 infection and disease severity will provide important insights into the immune mechanisms preventing infection.

However, a growing number of studies have revealed that pervasive somatic mutations may occur in nonmalignant tissues, and not all genetic abnormalities lead to functional changes or increased susceptibility to diseases [147, 148]. Unlike the monogenetic determinants affecting *CCR5*, some genetic variants may act as “noise” and may not be good markers of disease conditions or biomarkers, resulting in poorly targeted immunotherapies [15, 20, 29, 41, 42, 45, 52–54].

### Epigenetic alterations in the regulation of chemokine genes

Nongenetic heterogeneity propagated by epigenomic and transcriptomic alterations facilitates cellular functional plasticity, tissue specificity and phenotypic diversity [6, 20, 30–33, 35, 37–39, 42, 43, 120, 121, 149, 150]. Many novel sequencing-based approaches have been developed to unravel the heterogeneous and diverse epigenetic mechanisms, which has increased the understanding of the evolutionary and ecological roles of ‘nongenetic’ inheritance (NGI) [151–155]. The identification of epigenetic markers and distinct epigenotypes related to health and disease conditions can help identify promising strategies for disease management. Here, we summarize recent findings and discuss current concepts related to the role of chemokine epigenetics in the regulation of immune surveillance, host protection and tissue development.

#### Epigenetic regulation of gene expression

##### Common epigenetic mechanisms

Among the numerous intracellular mechanisms and mediators, epigenetic alterations, that is, nongenetic heritable alterations, play an indispensable role in regulating chemokine molecules; some epigenetic factors are key determinants of immune cell migration and memory, development and homeostasis [6, 31–33, 149, 150, 156], thus being defined as the “epiregulome” [149, 150]. Epigenetic events affect diverse gene regulation mechanisms leading to epigenetic modifications, as well as remodeling and modification of the conformation of chromatin architecture [150, 157–166]. Chemokine epigenetic marks can be combined with reference epigenomes to define cell function and identity with high resolution and spatiotemporal dynamics and in a cell type/tissue-specific manner [31–33, 167, 168]. Cell type/tissue-specific epigenomic patterns and transcriptional patterns define immune cell lineages and can be used in future studies of the role of chemokines in immune dysregulation in diseases and aging (Fig. 5A).

##### Epigenetic technologies

Many novel computational strategies can be used for analysis of data derived from chromatin immunoprecipitation (ChIP), micrococcal nuclease (MNase) and DNase I hypernasality site assays with next-generation sequencing (Fig. 5B) [152, 161, 162, 169–174]. ChIP assays and related technologies, such as chromosome conformation capture (3 C) coupled to sequencing (Hi-C), Hi-ChIP technologies, and chromatin interaction analysis by paired-end tag sequencing (ChIA-PET), are more accurate assays for detecting chromatin architecture at the genome scale [164, 175–179]. For instance, Hi-ChIP technologies have been employed to identify topologically associating domains (TADs), genomic regions organized by preferential interactions between chromatin and DNA sequences that play important roles in the proper control of chemokine gene expression by inducing the formation of chromatin loops. e.g., via promoter–enhancer interactions and super-enhancer (SEs).

#### DNA methylation

##### The levels of CpG methylation and demethylation

DNA methylation (DNAm), also called CpG methylation (CpGm) or 5-methylcytosine (5mC) modification, is a dynamic process catalyzed by members of the DNA methyltransferase (DNMT) enzyme family, which add methyl groups to the 5ʹ carbon of cytosine bases to create 5mC. Notably, demethylation of 5mC can occur throughout different physiological processes and is involved in many pathological conditions: 5mC is oxidized by ten-eleven translocation methylcytosine dioxygenases (TETs) to produce 5-hydroxymethylcytosine (5hmC), which has been shown to regulate the pluripotency of embryonic stem cells, neuron development, and tumorigenesis [180, 181].

##### Regulator of DNA methylation

In mammals, DNMT3A and DNMT3B respond to de novo methylation patterns early in development, while DNA methylation is maintained during cellular replication by DNMT1 interacting with ubiquitin-like with PHD and RING finger domain 1 (UHRF1), a key epigenetic regulator [182]. Recently, UHRF1 has been identified as a modulator suppressing multiple exacerbating factors in rheumatoid arthritis (RA) and found to contribute to negative feedback mechanisms that suppress multiple pathogenic events in arthritis, including epigenetic silencing of *CCL20*, a common UHRF1 target gene among cytokine-, RA-, and antiapoptosis-related genes. This suggests that the epigenetic mechanisms associated with the induction of RA-specific aberrations should be elucidated so that they can be controlled by epigenetic drugs for RA therapy [183].

The cooccurrence of DNMT-associated methylation and TET-associated demethylation confers methylation heterogeneity and is related to tumorigenesis; for example, tumor suppressor genes can be repressed by methylation rather than hypermethylation [184]. Therefore, cooccurrence of several factors, such as DNA methylation, may represent a unique layer of epigenetic regulation of gene expression that may facilitate breaking of symmetry during differentiation [181, 184–186]. Although an increasing number of studies have reported a role of reagent-induced or TET-mediated demethylation of chemokines in various disorders, such as *CXCL8* [187] in osteoarthritis, *Cxcl1* [188] in lung inflammation, and *CCL2* [189] and *CXCL12* [190] in carcinogenesis, further studies should consider the cooccurrence of several factors related to methylation, such as the ratio between the levels of methylation and demethylation (including 5-mC and 5hmC levels), to precisely interpret the regulatory effect of DNA methylation on chemokine expression in immune cells [181, 191–193].

Although many DNA methylation-associated chemokines have been found to be related to epigenetically driven pathways in the context of the specific immune microenvironment, few studies have focused on 5hmC modification of chemokines. A study using immunohistochemistry to detect 5hmC and T-cell-attracting chemokines in different-grade cervical lesions demonstrated that 5hmC was positively associated with the expression of T-cell-attracting chemokines (including CXCL9, CXCL10, and CXCL11) but negatively associated with the severity of cervical lesions, indicating that immunosuppression was present in precancerous cervical lesions [194]. Furthermore, 5hmC levels were increased in *CXCR4* gene bodies in colorectal cancer (CRC) compared to adjacent mucosa, although differential *CXCR4* methylation was not found [195]. Considering the therapeutic potential of the CXCL12-CXCR4/ACKR3 axis in cancer, 5hmC is a promising biomarker for precision medicine [196–198]. However, the challenge that remains is to develop innovative tools to reveal the differences between 5mC and 5hmC modification, which will enable more accurate data interpretation, as these modifications have different effects (5mC is a repressive mark, while 5hmC is an intermediate form of demethylation), and especially aid the development of techniques to interrogate circulating cell-free DNA (cfDNA) [191, 199].

##### Localization of DNA methylation underpins immune cell and tissue type specificity

That disruption of DNA methylation, not only CpG methylation density but also CpG methylation position, occurs early in tumors makes DNA methylation the best epigenetic marker, as it conveys information about health conditions and diseases, and targeting DNA methylation is a promising approach for disease management [158]. In addition to the well-known epigenetic silencing of tumor suppressive chemokines that results from promoter CpG island (CpGI) hypermethylation, CpG methylation can occur in CpGI shores, CpGI shelves, and open seas. Different methylation statuses exist in differentially methylated regions (DMRs), which contain multiple consecutive methylated CpGs and have implications for disease development and progression. These differentially methylated positions (DMPs) and/or DMRs are vital for tissue development and cell differentiation in a tissue-/cell-specific manner [200].

The DMPs and DMRs scattered throughout the genome also have functional implications that remain to be explored. For instance, CpG or CpGI methylation (iCpGIm) in the gene-body has opposite effects to pCpGIm, which affects mRNA splicing, contributing to transcriptome diversity [191, 201]. More tissue-specific DMRs are found in CpGI shores (~2 kb away from islands), the methylation of which shows a higher correlation with gene expression than the methylation of CpG islands [202]. In general, DNA methylation and demethylation regulate spatial and temporal gene expression (e.g., CpGI methylation silencing of tumor suppressor genes), impact chromatin remodeling (hypermethylated heterochromatin repeats), and are critical for embryonic development, lineage identity and cellular differentiation processes. Since epigenetic regulation of myeloid and lymphoid cell differentiation and function is important for appropriate host defense and organ homeostasis, which shape innate and adaptive immune responses, DNA methylation was proposed as “a transcriptional regulator of the immune system” [203]. The immune system has thus become a prototypical model for studying epigenetic effects on immune cell type- and stimulus-specific transcriptional programs, and relevant studies have generated a wealth of data [31, 161, 169, 170, 203]; furthermore, integrated analysis focusing on chemokine epigenetics may provide in-depth opinions about immune surveillance and homeostasis development. For instance, Roy et al. observed that differentially methylated sites were hypomethylated in innate immune cells but hypomethylated in adaptive immune cells [31]. These cell-specific differential methylation patterns may be used to define epigenetic states and gene expression profiles of innate and adaptive immune cell types that may underpin the functional differences of developmentally distinct cell types. Interestingly, that *CXCR5* has B-cell-specific DMRs reveals that cell-specific differentially methylated sites are associated with enhancer-related epigenetic marks (e.g., DNase I hypernasality sites, H3K4me1, and H3K27ac) but not with H3K4me3.

##### Differential CpG site methylation in health conditions and diseases

The distribution of DNA methylation is a main consideration when selecting methodology, designing experiments and performing bioinformatic analysis [200, 204]. Epigenome-wide association studies (EWAS) have increased ability to measure global CpG methylation and are thus useful for uncovering context-dependent regulatory roles of chemokines [205–207]. Using system-level approaches, relevant studies of epigenetic epidemiology have revealed extensive DMPs in chemokine genes that are phenotypically associated with different health conditions and diseases (Table S3, Table 6). Furthermore, these DMPs could be combined to develop aging- or perinatal-related risk factors for chemical hazard (such as air pollution) assessment. These methylation-driven chemokine gene signatures may be prognostic biomarkers in immune and genetic, metabolic, neurological and mental disorders and cancer (Fig. 5B, C) (Table 7).

**Table 6:** Differentially methylated CpG sites occurring in chemokines and receptors are associated withhealth conditions and diseases

The categorization of health condition or disease-associated phenotypes/traits is highlighted with different colors as above

The data were selected from the literature since 2019, and detailed information is shown in Table S3. In the phenotypes/trait, RE: the correlation with positive, BLAC: negative, or BLUE: no indication (NA).

*CAD* coronary artery disease, *CD* Crohns disease, *HDP* hypertensive disorders in pregnancy, *HNSCC* head and neck squamous cell carcinoma, *SLE* systemic lupus erythematosus

**Table 7:** Overview of selected clinical studies of agents targeting chemokines and receptors

Target	Drug	Mechanism of Action	NCT	Status	Condition or Disease	Phase
CCR2/CCR5	Cenicriviroc (CVC) (TAK-652; TBR-652)	dual **antagonist** of CCR2/CCR5	NCT04593940	Completed	Covid19	Phase 3
CCR4	Mogamulizumab (KW-0761)	humanized monoclonal **antibody** that binds to CCR4	NCT01728805	Completed	Cutaneous T-Cell Lymphoma	Phase 3
CCR5	Vicriviroc	**antagonist** of CCR5	NCT00523211	Completed	HIV Infections|Acquired Immunodeficiency Syndrome	Phase 3
			NCT00474370	Completed	HIV Infections|Acquired Immunodeficiency Syndrome	Phase 3
CCR5	Maraviroc	CCR5 **antagonist**	NCT02881762	Completed	Hepatitis C|Human Immunodeficiency Virus	Phase 4
			NCT02159027	Completed	AIDS Dementia Complex	Phase 2|Phase 3
			NCT01389063	Unknown	Endothelial Dysfunction	Phase 4
			NCT01866267	Completed	Human Immunodeficiency Virus|AIDS	Phase 4
			NCT01190293	Completed	HIV Infection	Phase 4
			NCT01449006	Completed	Human Immunodeficiency Virus (HIV) | HIV Associated Neurocognitive Disorders (HAND)	Phase 4
			NCT03402815	Completed	HIV Infection With Other Conditions|Cardiovascular Risk Factor|Atherosclerosis|Inflammation	Phase 4
			NCT01235013	Unknown	HIV-1 Infection	Phase 4
			NCT01348308	Completed	HIV-1 Infection|AIDS	Phase 3
			NCT00884858	Completed	HIV Infections	Phase 4
			NCT00666705	Completed	Healthy	Phase 4
			NCT00735072	Completed	HIV Infection	Phase 4
			NCT00853840	Completed	AIDS	Phase 4
			NCT01896921	Completed	HIV	Phase 3
			NCT00875368	Completed	HIV Infections	Phase 4
			NCT03178084	Completed	HIV/AIDS	Phase 3
			NCT01327547	Completed	HIV Coinfection	Phase 4
			NCT01384682	Completed	HIV	Phase 4
			NCT00966329	Completed	HIV | HIV Infections	Phase 4
			NCT01275625	Completed	HIV	Phase 4
			NCT00870363	Completed	HIV Infections	Phase 4
			NCT00426660	Completed	HIV Infections	Phase 3
			NCT01680536	Completed	HIV	Phase 4
			NCT03129113	Completed	Hepatic Steatosis|HIV-1-infection	Phase 2|Phase 3
			NCT01013987	Unknown	HIV-1 Adults Patients|AIDS|Triple Class Failure	Phase 4
			NCT00478231	Completed	Acquired Immunodeficiency Syndrome|HIV Infection	Phase 3
			NCT00925756	Completed	HIV Infections	Phase 4
			NCT00808002	Completed	HIV Infections	Phase 3
			NCT00844519	Completed	HIV Infection|Cardiovascular Disease|Inflammation|HIV Infections	Phase 3
			NCT01533272	Completed	HIV Infection	Phase 4
			NCT00717067	Completed	Human Immunodeficiency Virus (HIV) Infection	Phase 4
			NCT02519777	Completed	HIV Infections	Phase 4
			NCT01060618	Completed	HIV Infections	Phase 2|Phase 3
			NCT00098293	Completed	HIV-1	Phase 3
			NCT00098722	Completed	HIV Infections	Phase 2|Phase 3
			NCT00098306	Completed	HIV Infections	Phase 2|Phase 3
			NCT00098748	Completed	HIV Infections	Phase 2|Phase 3
			NCT03218592	Completed	HIV/AIDS	Phase 4
			NCT01154673	Completed	Acute HIV Infection	Phase 2|Phase 3
			NCT01637259	Completed	Proteinuria|HIV	Phase 4
			NCT01367236	Completed	HIV|Impaired Cognition	Phase 4
			NCT04965662	Completed	HIV-1-infection	Phase 4
			NCT01033760	Completed	HIV-1 Infections	Phase 3
			NCT01378910	Completed	HIV	Phase 4
			NCT00935480	Completed	HIV Infections	Phase 3
			NCT00624195	Completed	HIV Infections	Phase 2|Phase 3
			NCT02302547	Completed	HIV	Phase 3
			NCT02588820	Unknown	HIV Infections	Phase 3
			NCT00537394	Completed	HIV Infections	Phase 3
			NCT02016924	Recruiting	Acquired Immune Deficiency Syndrome (AIDS) | HIV Infections	Phase 2|Phase 3
			NCT03631732	Completed	HIV-1 Infection	Phase 3
			NCT02121795	Completed	HIV-1 Infection	Phase 3
			NCT02469246	Completed	HIV-1 Infection	Phase 3
			NCT00708162	Completed	HIV Infection	Phase 3
			NCT02859961	Active,	HIV	Phase 2|Phase 3
CCR5	Leronlimab (PRO140)	a humanized monoclonal **antibody** to CCR5	NCT04901676	Suspended	COVID-19 Pneumonia	Phase 3
			NCT04901689	Suspended	COVID-19 Pneumonia	Phase 3
			NCT03902522	Active,	HIV-1-infection	Phase 2|Phase 3
			NCT02859961	Active,	HIV	Phase 2|Phase 3
			NCT02990858	Active,	Hiv|Human Immunodeficiency Virus	Phase 2|Phase 3
			NCT02483078	Completed	HIV	Phase 2|Phase 3
			NCT05271370	Active,	HIV-1-infection	Phase 2|Phase 3
CCR9	Vercirnon (CCX282-B; GSK1605786)	**antagonist** of CCR9	NCT01277666	Completed	Crohn’s Disease	Phase 3
CXCR1/CXCR2	Ladarixin	dual CXCR1 and CXCR2 **antagonist**	NCT04628481	Recruiting	Drug: Ladarixin|Drug: Placebo	Phase 3
CXCR1/CXCR2	Reparixin	CXCR1/2 **antagonist**	NCT05254990	Recruiting	COVID-19 Pneumonia|Sars-CoV-2 Infection	Phase 3
			NCT04878055	Completed	Pneumonia, Viral	Phase 3
			NCT01967888	Completed	Pancreatectomy for Chronic Pancreatitis	Phase 2|Phase 3
			NCT01817959	Completed	Islet Transplantation in Diabetes Mellitus Type 1	Phase 3
CXCR4	Plerixafor (SDZ-SID-791; JLK-169; SID-791; AMD3100, AMD-3100, JM-3100, JM 3100; trade name Mozobil)	**antagonist** of CXCR4	NCT02056210	Completed	Diabetes	Phase 4
			NCT05087212	Recruiting	Autologous Haematopoietic Stem Cell Transplant	Phase 4
			NCT00838357	Completed	Lymphoma (Non-Hodgkin’s Lymphoma)|Hodgkin’s Disease or Multiple Myeloma|Front Line Mobilization|Transplantation	Phase 3
			NCT01164475	Completed	Non-Hodgkin’s Lymphoma	Phase 4
			NCT02006225	Unknown	Autologous Stem Cell Transplantation	Phase 4
			NCT01767714	Completed	Non-Hodgkin’s Lymphoma	Phase 3
			NCT02231879	Completed	Myelokathexis|Infections|Neutropenia|Warts|Hypogammaglobulinemia	Phase 2|Phase 3
			NCT00103662	Completed	Multiple Myeloma	Phase 3
			NCT00103610	Completed	Lymphoma, Non-Hodgkin	Phase 3
			NCT01146834	Completed	Multiple Myeloma	Phase 3
			NCT04000698	Recruiting	Refractory Acute Myeloid Leukemia|Refractory Acute Lymphoblastic Leukemia	Phase 3
			NCT04293185	Recruiting	Sickle Cell Disease	Phase 3
CXCR4	AMD-070 (AMD11070; AMD070; X4P-001; Mavorixafor)	**antagonist** of CXCR4	NCT03995108	Active,	WHIM Syndrome	Phase 3
	BL-8040 (Motixafortide; TF-14016; BKT-140; T-140)	**antagonist/inhibitor** of CXCR4	NCT03246529	Active,	Multiple Myeloma	Phase 3
CXCR4	POL6326 (Balixafortide TFA)	CXCR4 **Antagonist**	NCT03786094	Active,	Metastatic Breast Cancer|Locally Recurrent Breast Cancer	Phase 3
CCL5	OTR4120 (CACICOL20)	Glycomimetic	NCT02119039	Completed	Keratoconus	Phase 4

Data source: clinicaltrials.gov (https://www.clinicaltrials.gov/). The selected drug was ongoing over phase III or completed. In the column of NCT, green: completed; black: ongoing

In the column of mechanism, bold black: antagonist, bold blue: antibody

Studying the DMPs in chemokine clusters will help to elucidate relevant epigenetic mechanisms underlying their effects on immune gene regulation, and the results will highlight the importance of accounting for cellular heterogeneity and phenotypic diversity in chemokine biology. As shown in Fig. 5D, most of the differentially methylated CpGs in the *CCR5/CCR2* gene cluster are located in intergenic regions of CCR genes, which may contain interspersed repetitive sequences (IRSs) or functional elements (e.g., tissue-specific enhancers or SEs). Their epigenetic disruption may affect the expression of chemokines that are linked to diseases. IRSs (e.g., LINE-1, SINE-1, and Alu elements) are identical or nearly identical tandem DNA repeats that are disseminated throughout the genome; they are often packaged in heterochromatin or exist in regulatory and intragenic regions as a result of transposition or retrotransposition events. These elements were originally called “junk” repeats, but they are now recognized to represent a large source of individual variation among humans, and long stretches of these elements are usually called CNVs. Aberrant methylation of IRSs has been shown to alter chromosomal stability and cause genetic variations and abnormal RNA splicing and expression, thus playing a role in chemokine-mediated immune disorders and carcinogenesis [208, 209]. For instance, LINE-1 and other repeats were found to be hypomethylated in lymphocytes and neutrophils from patients with systemic lupus erythematosus (SLE) [210], possibly affecting SLE-related genes, and this finding may have implications for diagnosis or immune system modification in immunity and inflammation.

Since the CCR5/CCR2 gene cluster acts as a central regulatory region, it might be a useful model for studying disease-associated epigenetic alternations and genetic variants controlling chemokine expression and function to identify cell-specific enhancers buried in intergenic regions [207, 211]. As mentioned, dissection of global site-specific methylation patterns related to transcription factors, other epigenetic modifications, and gene expression in human immune cell types showed differential methylation sites in enhancer-related DMRs of *CXCR5* that defined cell specificity [31].

#### RNA methylation

Chemical modifications of ncRNA and N^6^-methyladenosine (m^6^A) are novel epigenetic modifications that can be studied to decipher functional correlations between mRNAs and certain biological processes, including cell differentiation and cell fate determination, a field termed “epitranscriptomics” [160]. For instance, the hypoxia-induced m^6^A demethylase alkB homolog 5 (ALKBH5) removes m^6^A and stimulates tumor macrophage recruitment and tumor immune escape through epigenetic and epitranscriptomic upregulation of *CXCL8* in glioblastoma [212]. ALKBH5 in neutrophils can be downregulated during bacterial infection. ALKBH5-mediated m^6^A promoted the migration capability of neutrophils by altering RNA decay, affecting the protein expression of its targets (for example, upregulating the expression of the neutrophil migration-promoting factor CXCR2 and downregulating the expression of the neutrophil migration-suppressing GPCR PTGER4). Therefore, activation or upregulation of the ALKBH-5-m6A demethylation axis is an intrinsic mechanism that drives efficient neutrophil migration [213]. Genome-phenome studies of the chemokines that dominate chemokine biological and regulatory pathways are needed to identify disease-specific epigenetic markers and targets [31, 214–217].

#### Epigenetic modifications

##### Super-enhancer regulation of chemokines and receptors

Studies of epigenetics using innovative techniques have revealed that promoter-enhancer compatibility is important in higher-order chromatin structures, e.g., three-dimensional (3D) chromatin loops known as TADs may recruit and stabilize transcription factor complexes to exert long-range gene transcriptional regulation [177, 178, 218–221], and most regulators binding distal enhancers in intronic or intergenic regions regulate tissue-specific pathways and drive condition-specific gene expression, ultimately determining cell identity [218, 220, 222]. SEs are large clusters of enhancers with aberrantly high levels of transcription factor binding and are thus critical for cell type specification and oncogenic transcription [223–226]. The epigenetic reader protein bromodomain‐containing protein 4 (BRD4) belongs to the family of bromodomain and extraterminal (BET) chromatin proteins, which are important targets for small molecule compounds [227, 228]. In addition, an in vivo study provided proof-of-concept for targeting BRD4 with a cell-permeable small molecule (JQ1) in NUT midline carcinoma (NMC), an aggressive squamous carcinoma that develops due to a fusion oncogene (*e.g*., *NUT* in frame with *BRD4*) [229]. I-BET, a synthetic compound that selectively binds BET, showed the capacity to interfere with the binding of BETs to acetylated histones to disrupt the formation of the chromatin complexes. For example, I-BET induced highly selective suppression of the expression of key LPS-inducible cytokines (*Il6*, *Ifnb*, *Il1b*, *Il12a*) and chemokines (*Cxcl9* and *Ccl12*) as well as the chemokines *Ccl2-5 and Cxcl1/2*, but did not affect the cytokine *Tnf*, in bone marrow-derived macrophages (BMDMs). However, treatment of BMDMs with I-BET suppressed the expression of TNF-inducible key proinflammatory cytokine (*Il1b*, *Il1a*) and chemokine genes (*Ccl5*, *Cxcl10*, *Cxcl2/3*) associated with epigenetic modifications and CpG content and that contribute to sepsis pathogenesis, conferring protection against LPS-induced endotoxic shock and bacteria-induced sepsis [230, 231].

Dysregulation of the inflammatory response disrupts the tissue homeostasis resulting from coordinated epigenetic regulation of the master transcription factor NF‐κB, rapidly inducing inflammatory gene expression [232, 233]. In human umbilical vein endothelial cells (HUVECs), the key inflammatory factor TNF‐α, induces the formation of large NF‐κB‐bound enhancer clusters (NF‐κB‐SEs) associated with active histone marks (H3K27ac), and BRD4 forces the expression of proinflammatory genes, including chemokine genes [231]. A recent study showed that TNF‐α rapidly induces co‐occupancy of lysine demethylases 7 A (KDM7A) and 6 A (UTX) at NF‐κB‐associated SEs in human ECs, which is essential for activation of NF‐κB‐dependent inflammatory genes, such as demethylated KDM7A H3K9 in the target genes *CXCL2* and *CXCL8* and demethylated UTX H3K27 in *CCL2*. As exemplified by *CXCL8* and other gene loci, Hi‐C in combination with ChIA‐PET revealed that TNF‐α‐responsive SE‐SE interactions were newly formed within sub‐TADs with decreased levels of H3K9me2 and H3K27me3 in SEs immediately following TNF‐α stimulation. These data suggest that coordinated demethylation of H3K9 and H3K27 by KDM7A and UTX might be functionally involved in the formation of SEs and the chromosomal conformation changes that activate their associated genes during early inflammatory responses in human ECs [234, 235]. Interestingly, the vital roles of KDM7A and UTX in the regulation of TNF-NF-κB axis-dependent inflammatory genes were found to be regulated by a TNF-responsive microRNA, miR-3679-5p. This is in line with the results of an integrative meta-analysis of the relationship between SEs and miRNA networks, which showed that SEs mark cell-type-specific miRNAs associated with cancer hallmarks, suggesting that SEs are major drivers of the tissue-specific miRNome [236].

Along the same lines, Fanucchi et al. showed that TNF-responsive genes, including chemokine genes, are arranged in TADs to form chemokine-SEs [237, 238]. These chromosome loops allow chemokines located in different chromosomes to form chemokine-SEs that are spatially available to be regulated by a subset of lncRNAs expressed within the TADs of HUVECs, termed immune gene-priming lncRNAs (IP-lncRNAs or IPLs). IPLs can direct the WD repeat-containing protein 5 (WDR5)–mixed lineage leukemia protein 1 (MLL1) complex across multiple chemokine promoters (e.g., *CXCL8*, *CXCL1*, *CXCL2* and *CXCL3* in human 4q21) by forming *cis* contacts with TNF-responsive genes associated with H3K4me3. One particular IPL, upstream master lncRNA of the inflammatory chemokine locus (UMLILO), forms the UMLILO–WDR5–MLL1 axis in the *cis* regulation of H3K4me3 modification at CXCL chemokine promoters within the same TAD. TNF-activated UMLILO is also related to a classic inducer of trained immunity, β-glucan, which can increase the transcription of several IPLs and chemokines to train immunity responses. Moreover, UMLILO is absent in mouse CXC-chemokine SEs, and mice lack β-glucan-trained immune responses. Insertion of UMLILO into mouse chemokine SEs resulted in training of CXCL genes with H3K4me3 epigenetic accumulation. Considering the differences in CXCL gene loci between mice and humans, this study may partly explain why mice are more resistant to inflammatory stimuli than humans. The study supports the epigenetic regulation of InscRNAs by chemokines [239] and provides strong evidence that UMLILO–WDR5–MLL1 axis-mediated chromatin looping of CXC-chemokine SEs controls immune gene priming in response to innate immune cell signaling to generate a nonspecific enhanced response to pathogen reinfection.

By using ChIP–seq and 4C-seq and analyzing published databases, a putative SE for multiple CXCLs located 20 kb upstream from the CXCL gene loci was identified in alcoholic hepatitis (AH) and found to orchestrate TNFα/NF‐κB-induced upregulation of CXCL chemokines (e.g., CXCL1, CXCL6 and CXCL8, related to neutrophil recruitment and infiltration) associated with active histone modifications in liver sinusoidal endothelial cells (LSECs), a major source of CXCL chemokines regulated by the TNFα/NF-κB signaling axis in the liver. BET inhibitors suppressed the expression of CXCLs by inhibiting transcription factor binding at CXCL SE and promoter sites. These high-throughput epigenomic studies in both humans and mice support a conserved role for CXCL SEs in regulating CXCL gene involvement in propagating inflammatory signaling by inducing chemokine expression and show the therapeutic potential of BET inhibition in AH treatment [240]. Owing to their broad activity against a large number of inflammatory genes and their specificity for their target genes, SEs are attractive candidates for pharmacological intervention [164, 218, 240].

##### Epigenetic modifications of chemokines in tumor-infiltrating lymphocytes (TILs)

**Polycomb group (PcG) proteins** are crucial epigenetic regulators that function as transcriptional repressors via two main epigenetic complexes, polycomb repressive complex 1 (PRC1) and PRC2, the aberrant activity of which is involved in carcinogenesis. The core components of PRC2 include embryonic ectoderm development (EED), suppressor of Zeste 12 homolog protein (SUZ12) and enhancer of Zeste homolog 1/2 (EZH1/2). EZH1/2 have a Su(var) 3–9, enhancer-of-zeste and trithorax (SET) domain with histone methyltransferase activity that monomethylates, dimethylates or trimethylates lysine 27 of histone H3 (H3K27me1/2/3). PRC2 exerts repressive effects by binding to the repressive marker H3K27me3 to repress expression from neighboring nucleosomes. PcG proteins can form distinct multiprotein complexes in various contexts, such as in early development, during an immune response, and cancer and play a role in proliferation-differentiation balance and metabolism. PcG proteins thus provide the basis for mechanistic divergence, and interfering with PcG functions may be a powerful strategy to counter tumor progression [241, 242].

**Trafficking of T cells to tumors** Tumor-infiltrating lymphocytes (TILs) are key players generating “hot” tumor microenvironment (TMEs), and chemokines direct the trafficking of T cells and other immune cells [243]. TILs are more responsive to immunotherapy combined with inhibitors of programmed cell death protein 1 (PD1) and its ligand PDL1 [244]. Impaired intratumoral accumulation of T cells in the TME leads to poor cancer immunotherapy efficacy and resistance, and chemokines, e.g., CCL5, CXCL9, CXCL10, and CX3CL1, are crucial for T-cell infiltration due to their ability to induce migration of immune cells [245–247]. An increasing number of studies have recently revealed the importance of the epigenetic modification of chemokines in the specific regulation of the trafficking of T cells to tumors.

**Epigenetic modification for T-cell trafficking and PD-L1 checkpoint blockade** A study showed that EZH2-mediated H3K27me3 modification and DNMT1-mediated DNA methylation block ovarian tumor production of the Th1-type chemokines CXCL9 and CXCL10 (CXCL9/10) and subsequently enable effector T-cell trafficking to the TME. Combined inhibition of EZH2 and DNMT1 augmented the expression of the inflammatory chemokines CXCL9 and CXCL10, which increased TILs and decreased tumor progression, thus improving the therapeutic efficacy of PD-L1 checkpoint blockade and adoptive T-cell transfusion in tumor-bearing mice [248]. In addition, epigenetic silencing of the Th1-type chemokine *CXCL9/10* via deposition of H3K27me3 mediated by PRC2 components (EZH2, SUZ12 and EED) impaired T-cell trafficking toward colon tumors, suggesting that PRC2/H3K27me3-mediated Th1-type chemokine silencing is a novel immune evasion mechanism in human colon cancer. Therefore, epigenetic restoration of repressed Th1-type chemokine expression to enhance T-cell infiltration into tumors may improve the clinical efficacy of cancer therapy [249]. Consistent with these reports, a class of pyrimidone compounds, represented by BR-001, was recently found to exert antitumor effects by upregulating CXCL10 to trigger CD8^+^ T-cell trafficking toward tumor sites. This may be associated with the capacity of BR-001 to directly bind EED in the H3K27me3-binding pocket to disrupt the EED-H3K27me3 interaction. Although no synergistic effect was observed in the BR-001 and anti-PD-1 combination group, the study suggests that the regression of colon tumors may be induced by inhibiting PRC2 modulation of the tumor immune microenvironment [250].

Downregulation of interferon-γ inducible protein 16 (IFI16), a direct target of EZH2, decreases stimulator of interferon genes (STING) activation and downstream CXCL10/11 expression in response to trastuzumab treatment in HER2+ breast cancer (BC). Dual inhibition of EZH2 and histone deacetylases (HDACs) significantly activated IFI16-dependent immune responses to trastuzumab. Another combination strategy, a novel histone methylation inhibitor combined with an HDAC inhibitor, induced complete tumor eradication and long-term T-cell memory in a HER2 + BC mouse model. These findings reveal the IFI16-CXCL10/11 signaling pathway as the crucial pathway conferring anti-HER2 trastuzumab resistance, and this pathway can be epigenetically targeted by EZH2 and HDAC inhibitor combination therapy to induce complete tumor eradication through increased CD8 + T-cell infiltration and induction of long-term T-cell memory in HER2+ breast cancer [251]. An analysis of TCGA data from clinical specimens from patients with triple-negative breast cancer (TNBC) showed that the expression of immune regulatory genes, including CD8 + T-cell attracting chemokine genes (*CCL5*, *CXCL9*, *CXCL10*) and the gene encoding the immune checkpoint molecule *PD-L1*, was negatively associated with the levels of histone lysine specific demethylase 1 (LSD1). Furthermore, LSD1 inhibition resulted in H3K4me2-induced restoration of immune regulatory gene expression, which in turn increased CD8 + T-cell tumor infiltration to overcome resistance to immunotherapy [252].

##### Epigenetic regulation of the CCL19/21-CCR7 axis in dendritic cells (DCs)

CCR7, coupled with its natural ligands CCL19 and CCL21 (the CCL19/21-CCR7 axis), controls the trafficking of DCs and metastasis and invasion of some malignant tumor cells [6, 253–255]. Abnormal DC trafficking results in immune pathologies, including autoimmune responses, infectious diseases, allergic diseases and cancer [6, 256]. Epigenetic modifications such as the transcriptionally repressive H3K27me3 modification associated with *Ccr7* were shown to determine the migratory capacity of distinct DC subsets (migratory conventional DCs vs nonmigratory bone marrow DCs) [257] and affect epigenetic alteration of *CCR7* and *CXCR4* in tumor cells [258], and the NAD-dependent deacetylase sirtuin 6 (SIRT6) may promote the ability of CXCR4-positive DCs to migrate to the afferent lymph nodes in the development of experimental autoimmune encephalomyelitis (EAE) [259].

A recent study was possibly inspired by the role of lncRNAs in the epigenetic regulation of chemokine signals; for example, breast cancer antigen-resistance 4 (BCAR4) mediates cooperative epigenetic regulation of the CCR7-CCL21 axis to promote tumor cell migration [239] and regulates DC differentiation by interacting with transcription factors [260]; the study identified epigenetic regulation of the timely termination of DC trafficking at the late stage to prevent unwanted inflammation [261]. CCR7 mediates rapid but transient DC migration to initiate protective immunity and maintain immune homeostasis. In addition to the well-established CCR7-triggered DC recruitment during the early stages of immune defense against invading pathogens, CCR7 stimulation also upregulates the long noncoding RNA *Lnc-Dpf3* via m^6^A demethylation to prevent its degradation, and *Lnc-Dpf3* feedback directly binds the transcription factor hypoxia-inducible factor 1-alpha (HIF-1α) and suppresses its activity to restrain CCR7-mediated DC migration and inhibiting glycolysis. This study provided important insights into the crosstalk between epigenetic mechanisms and metabolic pathways in regulating the network of DC-based immune responses. Therefore, understanding of the epigenetic regulation of CCR7-dependent DC migration is essential for developing therapeutic and vaccination strategies for inflammatory and autoimmune disease treatment.

#### Chromatin organization of chemokines in neutrophil extracellular traps (NETs)

##### NET formation and its inducing factors

Upon activation, neutrophils eliminate pathogens through phagocytosis, degranulation, and cytokine production. NETs are net-like extracellular fibers of processed chromatin (DNA-histone complexes) decorated with neutrophil-derived and adhered proteins that trap and neutralize microbes. NET formation follows a well-orchestrated cell death program called **NETosis**. During NETosis, neutrophils release large amounts of DNA and histones into tissues, where they can target microbes or serve as chemoattractants [99, 262–265]. The well-described role of histones as damage-associated molecular patterns (DAMPs), such as PAD4-mediated citrullinated histone H3 (**citH3**), contributes to the antimicrobial function and pathogenic effect of NETs. DNA, as a sticky polyanionic molecule, is capable of binding to bacterial cell walls for immobilization of pathogens on NETs to direct contact with cytotoxic molecules in the NET-DNA complex. Therefore, citH3 and cell-free DNA (**cfDNA**) are considered more specific NET markers under various disease conditions [264, 266–268]. Although NETs protect against infection, their inappropriate release is also implicated in the pathology associated with inflammatory and autoimmune diseases and cancer [98, 266, 267, 269–272]. As such, an understanding of NET formation and its inducing factors will enable the development of improved therapeutic targeting strategies, and NETs and their inducing factors represent a good model to study the epigenetic regulation of the inflammatory chemokines underlying dynamic changes in chromatin configuration and spatiotemporal remodeling.

##### NETs in SLE

The cfDNA structures released due to chromatin decondensation and spreading can also directly clog blood vessels and establish vessel-blocking thrombi or interact with anti-nuclear antibodies, forming immune complexes in SLE [273, 274]. SLE also features low-density granulocytes (LDGs) and increased levels of a pathogenic neutrophil subset. A detailed analysis of the bulk and single-cell transcriptomic, epigenetic, and functional profiles of lupus LDGs showed that lupus neutrophil subsets differed phenotypically and functionally in terms of NET formation, chemotaxis mediated by formyl peptide receptors 1 (FPR1), CXCR1 and CXCR3, and other processes, suggesting neutrophil heterogeneity and the putative role of neutrophils in the pathogenesis of SLE associated with vascular damage [274].

##### NETs in malignancy

**NET components in cancer** Experimental and clinical studies have revealed the presence of NETs and their components in a variety of cancers [275, 276]. The effect of NETs on malignancy and metastasis and the contribution of NETs to TME heterogeneity have attracted emerging interest. NET-DNA binds to CCDC25, a transmembrane DNA receptor, on tumor cells and enhances cell motility and facilitates NET-mediated distant metastases, revealing therapeutic target potential of targeting the cytoplasmic membrane DNA sensor for metastasis [277]. NETs induced by tumor-derived CXCL8 coupled with CXCR2 promoted diffuse large B-cell lymphoma (DLBCL) progression by activating Toll-like receptor 9 (TLR9), an important DNA sensor, and its downstream pathways. Aggressive interactions of tumor cells and NETs via the CXCL8–CXCR2 axis in DLBCL thus have implications for prognostication and targeting NET formation, and this crosstalk represents a new therapeutic target for this challenging disease [278] and other diseases; e.g., the HMGB1/RAGE/CXCL8 axis could be targeted to inhibit glioma progression [279]. Park et al. demonstrated that metastatic breast cancer cells can recruit neutrophils via the expression of CXCL1/2 and induce NET formation at sites of dissemination in the absence of infection. The NETs in turn support the spread of metastasis, and this could be inhibited by administration of DNase I-coated nanoparticles [280]. Inflammatory stimulants (e.g., CXCL1, CXCL2 and CXCL8) can stimulate neutrophil chemotaxis and activation to generate chromatin webs, thereby inducing NET formation in the omentum, a preferential metastasis site of ovarian cancer, while inhibition of NETs decreased the implantation of cancer cells [281].

**NETs and proteases** In addition to the IFI16-CXCL10/11 signaling pathway conferring anti-HER2 trastuzumab resistance [251], a study in a mouse model revealed that NET formation induced from sustained lung inflammation could convert dormant disseminated cancer cells (DCCs) into aggressive lung metastases by affecting NET-associated proteases, neutrophil elastase (NE) and matrix metalloproteinase 9 (MMP9), providing important insights into the microenvironmental control of DCC reactivation from dormancy, which could have therapeutic implications [282]. Indeed, proteases that actively degrade proinflammatory mediators have been shown to be enriched in NETs. As trypsin activation, leukocyte recruitment, and impaired microvascular perfusion participate in the pathophysiology of severe acute pancreatitis (AP) with systemic inflammation and lung damage, the relationship of NETs with trypsinogen activation-mediated inflammation and tissue injury was investigated in a mouse AP model induced by taurocholate or L-arginine [283]. Neutrophil depletion blocked taurocholate-induced deposition of NETs in the pancreas. The administration of DNase I to mice reduced neutrophil infiltration and tissue damage in the inflamed pancreas and lung, accompanied by decreased levels of blood amylase, IL-6, HMGB1 and CXCL2/MIP-2. The addition of NETs and histones to acinar cells induced the production of trypsin and STAT3. Notably, increased levels of cfDNA and DNA–histone complexes were found in the serum of AP animals and patients with severe AP. That NETs contribute to the development of AP and regulate organ inflammation and injury suggests that they might be a useful target for ameliorating local and systemic inflammation in severe AP. Therapeutic strategies directed against NET formation may provide a clinical benefit by reducing inflammatory tissue damage in patients.

The digestive activity of the trypsin enzyme may facilitate tissue inflammation and cell migration/metastasis in association with inflammatory chemokines [284, 285], and thus, it would be interesting to determine the interplay between trypsin family members and chemokines in chromatin dynamics during NETosis. A novel biohybrid platform that was recently developed by conjugating DNase I to a nonfouling microgel could be employed as a nonthrombogenic active microgel-based coating for blood-contacting surfaces to reduce NET-mediated inflammation and microthrombi formation [286], thus aiding monitoring of processes related to cell mobility, including inflammatory infiltration and cancer metastasis.

##### NETs for neutrophil self-limitation

In-depth studies of inflammation-related carcinogenesis have provided proof of concept for NET inhibition strategies for the prevention of thrombotic/vascular complications, cancer propagation, and severe infections, such as sepsis and COVID-19 [269, 272, 287, 288]; however, NETs are also involved in noninfectious, sterile inflammation and acute injuries associated with autoimmunity and cancer [289]. Furthermore, NETs participate in a powerful negative feedback mechanism that self-limits neutrophil activation by providing a temporary (pop-up) chemokine-degrading scaffold [268, 290, 291]. For instance, CXCL8-induced NETs have been preliminarily shown to contribute to cancer development and progression; furthermore, blockade of CXCL8 or its receptors (CXCR1 and CXCR2) is being pursued for drug development, and clinical trials of such drugs used alone or in combination with anti-PD-L1 checkpoint inhibitors are already ongoing [271]. Although NETs are highly dynamic and complicated chromatin structures, recent technological advances in strategies such as Hi-ChIP [152], spatially-resolved transcript amplicon readout mapping (STARmap), a 3D intact-tissue RNA sequencing [162, 292] may help us to dissect the epigenetic interactions between DNA and histones at high resolution and the epigenetic regulation of chemokine-mediated pathways. Owing to the central role of chemokines in the control of cell mobility, such studies will shed light on the immune response and tissue homeostasis and lead to the identification of translatable precision biomarkers and therapeutic targets.

Overall, these insights suggest that epigenetic modifications are dynamically controlled to regulate chemokine expression via specific inflammatory and homeostasis pathways and serve as reversible controls that have potential as therapeutic targets for disease prevention and management. However, researchers still need to develop convenient techniques to rapidly assess immune cell responses to treatments at single-cell resolution [293].

### Abnormal expression of chemokines and receptors

#### Differential expression of chemokines confers phenotypic heterogeneity

Aberrant expression of chemokines and receptors has been reported in various diseases, including inflammatory diseases and cancer [14, 18, 19, 294–296]. For example, the serum levels of the IFN-α-induced chemokines CCL2, CXCL10 and CCL19 were found to correlate with lupus patient age and disease duration and thus have implications for monitoring disease activity and the determining the degree of organ damage in SLE [297]. In contrast to low expression of the favorable prognostic marker *CX3CL1* induced by epigenetic silencing, expression of *CCL3*, *CCL8*, *CCL15*, *CCL18* and *CXCL9* was negatively correlated with prognosis and T-cell infiltration in nephroblastoma [298].

**Fig. 6:**
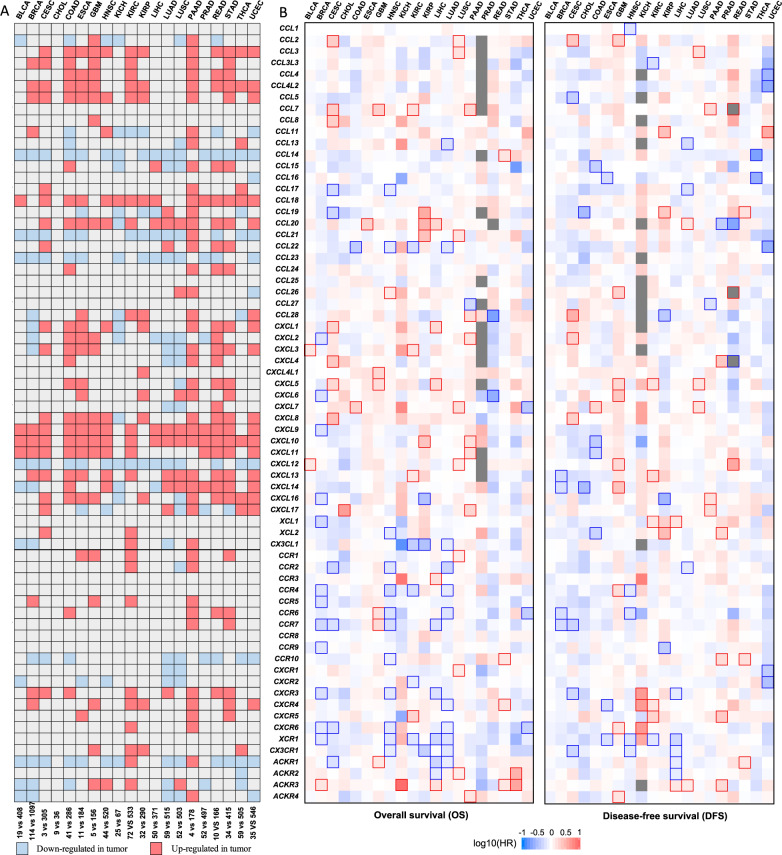
Chemokines and receptor expression and its association with clinical outcomes in human cancer. The associations of chemokine expression and receptor expression (**A**) with clinical patient outcomes (**B**) in multiple cancer types was identified using the limma method and the GEPIA tool (http://gepia.cancer-pku.cn/). Red: upregulated in tumor samples (log2FC > 1 and adjusted *p* < 0.05), blue: downregulated in tumor samples (log2FC < -1 and adjusted *p* < 0.05), gray: stable. BLCA bladder urothelial carcinoma, BRCA breast invasive carcinoma, CESC cervical squamous cell carcinoma, CHOL cholangiocarcinoma, ESCA esophageal carcinoma, GBM glioblastoma multiforme, HNSC head and neck squamous cell carcinoma, KICH kidney chromophobe, KIRC kidney renal clear cell carcinoma, KIRP kidney renal papillary cell carcinoma, LIHC liver hepatocellular carcinoma, LUAD lung adenocarcinoma, LUSC lung squamous cell carcinoma, PRAD prostate adenocarcinoma, READ rectum adenocarcinoma, STAD stomach adenocarcinoma, THCA thyroid carcinoma, UCEC uterine corpus endometrial carcinoma

Analysis of TCGA data showed differential expression patterns of chemokines and receptors in cancer patients with different clinical outcomes (Fig. 6), suggesting that cancer type-related transcriptional heterogeneity may cause functional heterogeneity affecting clinical outcomes, revealing potential prognostic targets for translational studies [20]. For example, decreased expression of *CXCL12* was associated with unfavorable overall survival (OS) and disease-free survival (DFS) in all types of cancer, whereas *CXCR4* was highly expressed in several cancer types. For example, in stomach cancer (STAD), high *CXCR4* expression was associated with favorable OS, while its high expression was associated with poor DFS in patients with kidney renal clear cell carcinoma (KIRC). An even more extreme example is that dysregulation of CCL19, a homeostatic chemokine that interacts with CCR7 to play a crucial role in the development of lymphoid organs [299] (Fig. 2), showed both tumor-suppressive and oncogenic effects in cancer. Despite there being no significant changes in *CCL19* expressed in an analysis of TCGA data, higher *CCL19* expression and secretion were found in metastatic nodes of patients with head and neck squamous cell carcinoma (HNSC) than in benign nodes or primary tumors, and the CCL19-CXCR5 axis was found to exert prosurvival signaling associated with tumor progression and disease relapse [300]. In contrast, CCL19 was expressed at significantly lower levels in CRC tissues. Upregulation of CCL19 expression could inhibit CRC angiogenesis by promoting inhibition of the Met/ERK/Elk-1/HIF-1α/VEGF-A pathway by miR-206, suggesting a novel therapeutic strategy for antivascular treatment in CRC [301].

Notably, new findings continue to improve the understanding of chemokine biology. For instance, CCL22 is a dual chemokine constitutively expressed or induced upon inflammation, serving as an antimicrobial protein (Fig. 2). CCL22-deficient mice display partially penetrant preweaning lethality (Table S1) and increased susceptibility to inflammatory diseases [302]. T-cell-derived cytokines maintain the constitutive expression of CCL22 at high levels in lymphoid organs during homeostasis [302]. CCL22 expressed on dendritic cells (DCs) interacts with CCR4 (CCL22-CCR4 axis) to mediate DC–T-cell contacts that are crucial for immune regulation by Tregs, suggesting that the CCL22–CCR4 axis is also an immune checkpoint and that targeting the interaction of CCL22 with its receptor may be an effective but less harmful therapeutic strategy [303]. A recent study showed that CCL22 was abundantly expressed by tumor-associated macrophages (TAMs) from humans in esophageal squamous cell carcinoma (ESCC) tissues. ESCC TAM-released CCL22 promoted tumor invasion and reduced patient survival via activation of the CCR4/DGKα/FAK complex in ESCC cells, revealing opportunities for targeting the tumor-promoting microenvironment to achieve anticancer effects [304]. Thus, the differential expression and regulation patterns of chemokines contribute to the site- and cell-specific divergent pathophysiological responses. Chemokines exert dual roles and produce paradoxical effects in the TME in a context-dependent manner; these roles and effects may confer functional tumor heterogeneity and thus phenotypic plasticity.

#### Alternative splicing (AS) contributes to phenotypic heterogeneity

##### An introduction to AS

**Fig. 7:**
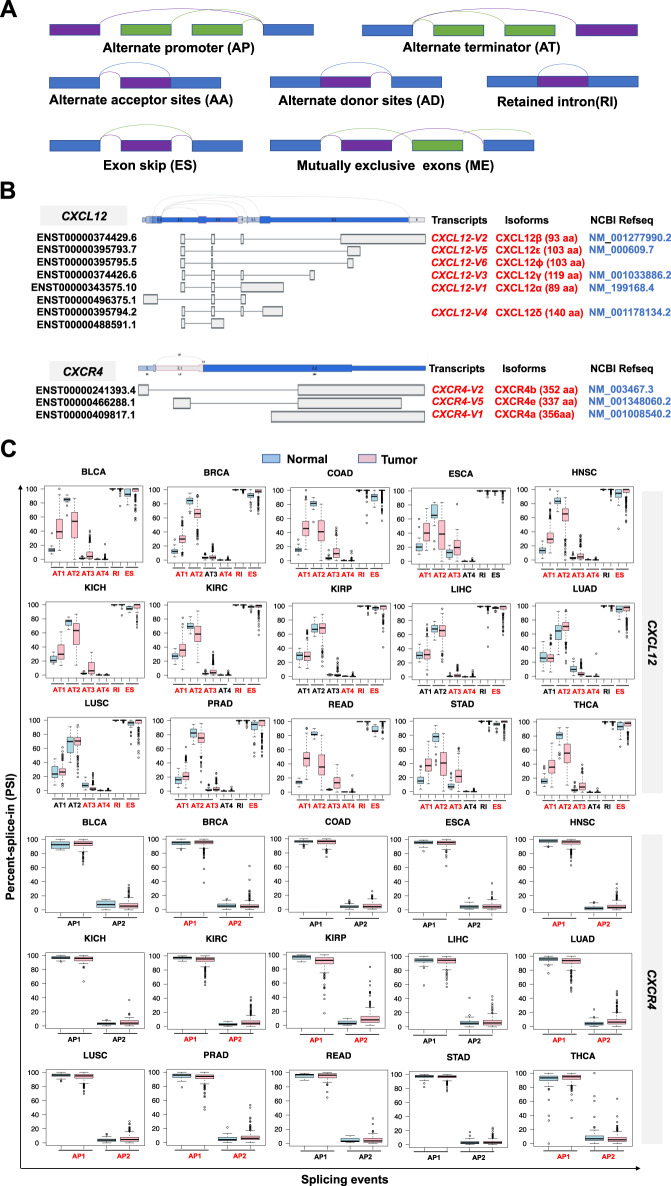
RNA splicing of chemokines and receptors. **A** Schematic representation of alternative splicing (AS) and different splicing events. Human protein-coding genes undergo AS through the use of alternate acceptor (AA) sites, alternate donor (AD) sites, alternate promoters (APs), alternate terminators (ATs), exon skipping (ES), mutually exclusive exons (ME), and retained introns (RIs), and the most common form of RIs is mutually exclusive exons (MEs), which allows constitutive splicing (Fig. 6A). **B** Schematic of the CXCL12 and CXCR4 transcripts. **C** Comparison of the alternative splicing events of CXCL12 and CXCR4 between multiple types of tumor and normal tissues. The data were extracted from TCGA RNA-seq data (https://bioinformatics.mdanderson.org/TCGASpliceSeq/). For each splicing event, the percent spliced in (PSI) was compared between normal and tumor samples by the Wilcoxon rank sum test, and splicing events with significant differences (*p* < 0.05) are marked with red labels. For CXCL12, AT1 is an AT event affecting exon 5.2; AT2 is an AT event affecting exon 3.3; AT3 is an AT event affecting exon 4; AT4 is an AT event affecting exon 6; RI is an RI event affecting exon 3.2; and ES is an ES event affecting exons 2.2, 3.1 and 5.1. For CXCR4, AP1 is an AP event affecting exon 1, and AP2 is an AP event affecting exon 2.1

Most human protein-coding genes undergo AS, a key transcriptional and posttranscriptional process that leads to the formation of multiple transcript variants or splicing variants (*SVs*) that exert diverse effects via multiple mechanisms, including nonsense-mediated mRNA decay (NMD) (Fig. 7A). These splicing events are functionally important for innate and adaptive immune responses [305, 306] due to their capacity to generate tissue- and cell type-specific or stimulus-responsive *SVs* [307–309], which have diverse or even opposing functions [310, 311]. Abnormal SVs preferentially produced in various diseases have been proposed as biomarkers for diagnosis and treatment, and studies of such SVs have revealed precision therapy approaches to correct disease-specific defects caused by mis-splicing [312, 313].

Over 77% (37 of 48) of chemokines and receptors have more than one transcript and protein isoform. Although splicing factors and the processing cascades necessary for spliceosome function are well known [307–309], most of the abnormal chemokine *SVs* detected at the transcriptional level can be translated into distinct protein isoforms. As confusion mounts over the role of RNA isoforms in functional diversity and phenotypic plasticity [314, 315], most chemokine transcript variants have not been studied, and their contribution to immune disorders and malignancy remains unknown [305, 306, 315]. A few notable examples of chemokine SVs with altered ligand-binding or signaling properties have been reported [316–322]. We summarize findings related to the CXCL12-CXCR4 axis as an example to illustrate the transcriptional heterogeneity that contributes to nongenetic phenotypic divergence.

##### The CXCL12-CXCR4 axis

***CXCL12-SV*** *CXCL12* is located on chromosome 10q11 and is broadly expressed in multiple tissues and cells (Figs. 2 and 3**)**. *CXCL12* has multiple transcript variants, five of which, *CXCL12-V1 to CXCL12-V5*, are currently NCBI-annotated transcripts. *CXCL12-V1 to CXCL12-V4* encode CXCL12 isoforms α to δ, while *CXCL12-V5* encodes CXCL12 isoform 5 or isoform **ε**. Another transcript, *CXCL12-V6*, encodes CXCL12 isoform ϕ, which is identical to isoform **ε**. *CXCL12-V1*, *CXCL12-V2* to *CXCL12-V6* are produced through AT, RI, ES, and their combination. Other transcripts (i.e., ENST00000395795.5) still remain to be experimentally validated (Fig. 7A, B) [323, 324]. It is interesting to note that three SVs have been identified in mice, different from the six SVs in humans.

***CXCR4-SVs*** *CXCR4*, located on chromosome 2q22.1, has five transcripts. *CXCR4-V3* has three exons encoding the longest CXCR4 isoform, isoform C. *CXCR4-V1* (also known as CXCR4-Lo) has only one exon transcribed through alternative promoters (APs), encoding CXCR4 isoform A. *CXCR4-V2* encodes CXCR4 isoform B, and *CXCR4-V5* encodes CXCR4 isoform E (Fig. 7B) [325–327]. CXCR4 displays diverse expression in the BM, lymph nodes, spleen and appendix and high expression in immune cells (Figs. 2, 3).

**CXCL12/CXCR4 functionality** CXCL12 induces diverse effects on hematopoietic progenitor cells, endothelial cells, and leukocytes by interacting with the classical receptor CXCR4 and the atypical receptor ACKR3/CXCR7 (Fig. 2) [323, 328]. Another atypical receptor, ACKR1/DARC, has also been shown to bind the CXCL12 dimer but not the monomer, and thus its binding is dependent on the differential expression of CXCL12 isoforms [329]. Deletion of *Cxcl12* or *Cxcr4* in mice results in a variety of developmental abnormalities and embryonic death (Table S1), whereas genetic variants of *CXCL12* or *CXCR4* are associated with resistance to HIV-1 infection and the development of WHIM (warts, hypogammaglobulinemia, infections, and myelokathexis) syndrome (Table S2) [330]. Therefore, the CXCL12/CXCR4/ACKR3 interaction in the chemokine network is indispensable for the development of hematopoietic and cardiovascular organs. In addition to being an essential player in embryogenesis, hematopoiesis, and angiogenesis, CXCL12 displays inflammatory functions in immune surveillance, the inflammatory response, autoimmune diseases, and tumor growth and metastasis [328]. In fact, the CXCL12/CXCR4 axis is among the most studied chemokine axes in cancer metastasis due to its capacity to support cancer cell proliferation, migration and invasion [324, 331].

**Differential expression of** ***SVs*** Differences in transcriptional related to cancer type may cause functional heterogeneity and differences in clinical outcomes that make it difficult to identify potential prognostic biomarkers for translational studies. CXCL12 is the most primitive chemokine and is highly conserved through evolution, and it may have diverse cellular functions in various biological processes because it has multiple SVs capable of encoding different isoforms [323, 324]. As shown in Fig. 7C, an analysis of the AS events of *CXCL12* and *CXCR4* transcripts between multiple tumor and normal tissues suggested divergent expression of *CCL12*- or *CXCR4-SVs* in tumor tissues. Compared to CXCR4, which showed a smaller difference in SVs, *CXCL12* displayed a significant difference in most splicing events in normal versus tumor tissues. The bulk expression of *CXCL12* was decreased in HNSC tissues but was not changed in STAD tissues, and it was not associated with patient clinical outcomes (Fig. 6). This may be because of different and even opposing changes in *CCL12-SVs*, such as an increase in the level of alternate terminator 1 (AT1) but a decrease in the level of AT2 (both of which effected the expression of *CXCL12*) in HNSC and STAD tumors, making the expression ultimately no different from that in controls (Fig. 7C). If the differentially expressed SVs have functional differences and are not distinguished, it may impair the final functional output or increase uncertainty risk. Although *CXCL12* is subjected to more posttranslational than transcriptional regulation [196, 332, 333], cell- or tissue-type specific RNA isoforms may be the cause of some of the controversial or paradoxical effects of chemokine‒receptors on different signaling pathways in immune and cancer cells under specific microenvironments.

Many transcripts exist per gene, most of which are thought to not be functionally relevant, and some even have opposing effects. For precise evaluation of clinical effectiveness and drug resistance, the specific expression of functionally distinct SVs, rather than their overall expression, should be considered for assay design to accurately reflect transcriptional heterogeneity. Therefore, the next round of translational studies in chemokine biology should focus on improving the understanding the differential expression and functionality of these transcript isoforms to guide the discovery and validation of biomarkers and targets.

## Chemokines and receptors for precision medicine

### Chemokines as noninvasive biomarkers for liquid biopsy

**Fig. 8:**
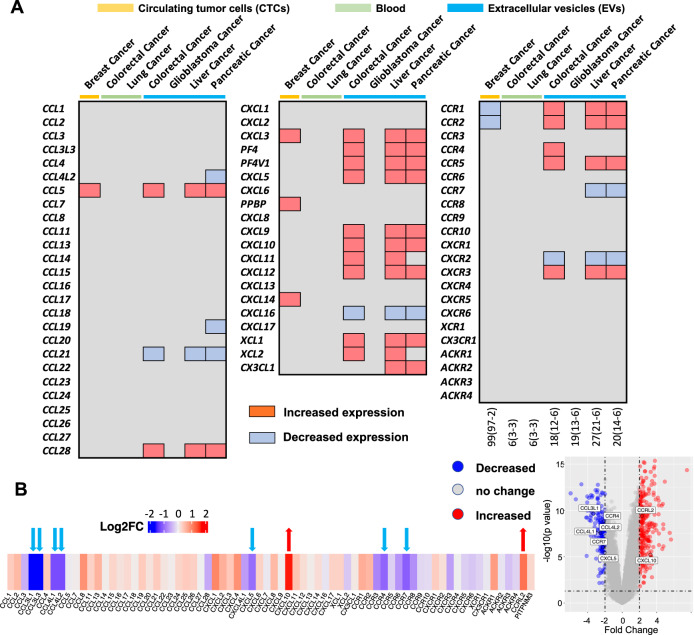
Chemokine molecules as potential noninvasive biomarkers. Heatmaps showing that differential expression of chemokines and receptors in tumor tissues from cancer patients compared to normal controls (**A**) or in COVID-19 specimens compared to healthy controls (**B**). The significant differences in between tumor tissues and normal tissues are shown in red (upregulation) or blue (downregulation) ( | log2FC | >1 & adjusted *p* value < 0.05). The data were downloaded from the Bbcancer database (http://bbcancer.renlab.org). The total sample number (tumor and normal samples) is shown at the bottom right. The color bars on the top indicate the sample type (yellow: CTCs; green: blood; blue: extracellular vesicles, EVs)

Chemokines have unique characteristics in cell mobility and immunity; for example, they establish concentration gradients and effect secretion under multiple layers of dynamic regulation, included genetic and epigenetic modification of chemokine genes (e.g., SNVs, chemokine-SEs and cfDNA). Due to their identifiable tissue specificity, chemokines may serve as ideal liquid biopsy-based biomarkers for early diagnosis or to guide targeted therapy for immune disorders and cancer [53, 334]. Figure 8A summarizes the differential expression of chemokines and receptors in liquid biopsy elements, including extracellular vesicles, circulating tumor cells (CTCs) and blood, of patients with several cancer types; the results suggest the potential of assessing chemokine expression by liquid biopsy. In addition, the serum levels of IFN-α-induced chemokines used to monitor SLE [297] and CX3CL1 methylation predicted T-cell infiltration in nephroblastoma [298]. As mentioned above, high serum levels of CCL3 and CCL4 and high CCR5 expression in primary specimens were found to be associated with poorer prognosis in patients with CRC [335]. The roles of CXCL13/CXCR5 and CCL22/CCR4 in multiple sclerosis (MS) and other autoimmune diseases have been reported [336, 337]. However, in an examination of a wide panel of cytokines and chemokines (CCL1, CCL2, CCL3, CCL22, CXCL11, CXCL13, and IL-16) in the cerebrospinal fluid of relapsing-remitting MS patients, only CCL3 was found to be associated with both MS diagnosis and oligoclonal IgG, a typical marker for inflammation in MS [338].

### Induction of chemokines and receptor expression by SARS-CoV-2

#### The CCL2-CCR2 axis

As mentioned above, SARS-CoV-2 infection in patients with poor clinical outcomes is characterized by high levels of proinflammatory cytokines and chemokines (e.g., IL-6, CCL2/MCP1, CCL3/MIP1α, CCL4/MIP1β and CXCL10/IP-10) and cytokine storms [85, 87]. CCL2 is an inflammatory chemokine that exerts both agonistic and antagonistic effects by binding to CCR2 expressed by monocytes/macrophages, plasmacytoid dendritic cells (pDCs), T cells and natural killer T (NKT) cells (Fig. 2). Trafficking of monocytes/macrophages and T cells is impaired by *Ccr2* deficiency (Table S1). The CCL2-CCR2 axis contributes to an immunosuppressive TME; thus, antagonistic drugs targeting CCR2 may be beneficial for cancer therapy or decrease undesired immune responses in COVID-19 and autoimmune diseases (i.e., nonalcoholic steatohepatitis) (Table S3). *CCL2* genetic and epigenetic alterations, such as *CCL2*-A2518G in COVID-19 [145], *CCR2* rs1799864 in HIV [117], and *CCL2*-SE [234, 235], are promising biomarkers for clinical translation. However, the CCR2 pathway was also found to promote viral control and restrict inflammation within the respiratory tract during SARS-CoV-2 infection [339], suggesting that CCR2 and its ligands have dual functions.

#### Inflammatory chemokines predicting severity of infection

Increased *CXCL8* plays a key role in promoting acute SARS infection, viral bronchiolitis, severe immunopathology, and respiratory syncytial virus (HRSV) infection disease progression [340]. However, an analysis of TGCA data showed decreased expression of *CCL3L1*, *CCL3L3*, *CCR4L1*, *CCR4L2*, *CXCL5*, *CCR4*, *CCR7* and *CXCR5*, but increased expression of *CXCL10* and *CXCRL2* was the most significant factor related to the host response to SARS-CoV-2 infection (Fig. 8B).

A recent study using scRNA-seq revealed differential expression of inflammatory cytokines in COVID-19 patients with different disease severities [36]. In addition to well-known cytokines (e.g., IL-1, IL-6 and IL-10), chemokines, including *CCL3*, *CXCL10*, *CXCL5*, and *CCR2*, were found to have increased expression in peripheral blood mononuclear cells (PBMCs) derived from COVID-19 patients with moderate, severe and critical disease, and the levels of CCL3 and CXCL10 were also assessed in plasma. While the expression of CCR6, CCR7 and *CXCR4* in PBMCs decreased with severity, the transcript levels of *XCL1*, *XCL2*, *CCL5* and *CXCR3* increased from moderate to severe disease in COVID-19 patients but returned to normal with the development of critical disease. Moreover, high levels of expression of favorable chemokine genes were observed in B cells (*CCL5*, *XCL1* and *XCL2*), T cells (*CCL4*, *CXCR3* and *CXCR6*) and monocytes (*CCL2*, *CXCL8* and *CXCL10*) in patients with moderate, severe and critical disease. Pivotal inflammatory chemokine receptor‒ligand pairs were found to mediate the intensity of interactions between CD8 effector T/NK cells and monocytes, as they were elevated in moderate and severe COVID-19 cases but diminished in critical cases. *CCL3L1-DDP4* was increased in critical cases, whereas *CCL3-CCR5*, *CCL4-CCR5*, *CCL4-SLC7A1* and *CCL4L2-VSIR* were enhanced in moderate and severe cases but decreased in critical cases. This study therefore suggests that inflammatory chemokines respond dynamically and nonredundantly to SARS-CoV-2 infection and that chemokine signatures may reflect disease severity and may be conducive to drug development [271].

#### Hypertension with COVID-19

Hypertensive patients are more likely to develop severe pneumonia or organ damage than patients without hypertension [341], and the cellular serine protease TMPRSS2 can prime the SARS-2-S protein for entry. An inhibitor of TMPRSS2, camostat mesylate, blocks SARS-CoV-2 infection of lung cells [342]. A recent observation showed that macrophages and neutrophils from hypertension patients with COVID-19 exhibited higher expression of the proinflammatory cytokines CCL3 and CCL4 and the chemokine receptor CCR1. Antihypertensive blockade of the renin–angiotensin–aldosterone system (RAAS), specifically with the use of an angiotensin-converting enzyme inhibitor (ACEi), might improve outcomes in patients with hypertension and COVID-19 [341, 343].

### Chemokines in targeted therapy

The therapeutic targeting of chemokines and receptors has been reviewed in several recent publications [41, 44–51, 344]. Current ongoing (later phases) and completed clinical trials of drugs targeting chemokines and receptors are listed in Table S3, and as can be seen, drugs targeting chemokine receptors are the major drugs used for antiviral therapy. Several clinical trials targeting chemokines are still in the early phases [47]. Targeting chemokine-receptor axes for precision therapy will require a comprehensive understanding of their differential expression and mechanisms in different tumor microenvironments, as targeting these axes may result in effects from target pathway redundancy and context-dependent immunosuppressive actions of the antagonist [45]. Readers should also refer to excellent specific review articles for more in-depth information [41, 43–52, 95].

#### CXCR4 antagonists

Based on the role of the CXCL12-CXCR4 axis in cancer metastasis, many CXCR4 antagonists for cancer therapy are in clinical development (Table S3). Of these, plerixafor is a bicyclam with hematopoietic stem cell mobilizing activity that selectively and reversibly antagonizes the binding of CXCL12 to CXCR4 on bone marrow stromal cells [345]. A phase II clinical trial is in progress to evaluate its use in combination with standard temozolomide chemoradiotherapy for patients with glioblastoma (NCT03746080). Plerixafor combined with granulocyte-colony stimulating factor (GCSF) has been shown to mobilize hematopoietic stem cells more efficiently than plerixafor alone [345]. BL-8040/motixafortide, a short, high-affinity synthetic peptide antagonist for CXCR4 with longer receptor occupancy, is being tested in a phase Ib/II trial (NCT02826486). This trial is investigating the safety, pharmacokinetics and anticancer activity of a combination immunotherapy in patients with advanced or metastatic gastric/gastroesophageal junction cancer/esophageal cancer.

#### The CCL3-CCR5 axis

As mentioned above, CCL3, CCL4, and CCL5 are HIV-suppressive factors produced by CD8-positive T cells to modulate virus-induced inflammation. CCL3 is produced by macrophages, CD4 + T cells, CD8 + T cells, NK cells, fibroblasts and mast cells and is an important activator of both innate and adaptive responses (Fig. 2). For example, atherosclerotic plaque-resident T cells differentially express several chemokine receptors that bind with their corresponding ligands to form CCL3-CCR5 and CX3 CL1-CX3 CR1 interactions, which induce T-cell migration into human atherosclerotic plaques, where T-cell accumulation contributes to plaque destabilization and atherosclerosis [346]. Ccl3 induced by administration of the antimitotic chemotherapy drug docetaxel (DTX) promoted proinflammatory macrophage polarization to suppress tumor progression and increased DTX chemosensitivity in breast cancer via the CCR5-p38/interferon regulatory factor 5 pathway [347]. CCL3 is also considered a neutrophil chemoattractant; it activates and enhances the cytotoxicity of NK cells and plays a critical role in both immune surveillance and tolerance by regulating lymph node homing of dendritic cell subsets and inducing antigen-specific T-cell responses [348]. For example, the innate immune mediators CCL3 and CCL4 were found to be elevated in the lungs of patients with chronic beryllium disease (CBD), a granulomatous lung disorder that is triggered in susceptible individuals by inhalation of beryllium-containing particulates. These chemokine-derived peptides may serve as neoantigen epitopes that can activate specific CD4 + T cells, thus revealing a direct link between persistent innate and adaptive immune activation [349]. However, CCL3 plays roles in both antitumor and protumor activities depending on the underlying signaling cascades that are activated through binding to the receptors CCR1, CCR4 and CCR5 and/or interacting with CCL4. For instance, the *β*-catenin-metadherin/CEACAM1-CCL3 positive feedback cascade has been shown to lead to metastasis in ovarian cancer by increasing the level of infiltrating tumor-associated macrophages (TAMs) at the metastatic site [350]. CCL3 derived from TAMs and cancer cells in esophageal squamous cell carcinoma (ESCC) promoted tumor cell migration and invasion via the CCL3-CCR5 axis and the PI3K/Akt and MEK/ERK pathways [351]. Therefore, the CCL3-CCR5 axis represents a potential therapeutic target for cancer treatment.

## Concluding remarks

Substantial progress has been achieved in chemokine biology, and multiomics data have enabled the identification of genome and metabolome profiles with complex regulatory networks and functional plasticity. A more comprehensive understanding of the chemokine interactome will not only enable more rational management of complex diseases but also promote the development of robust, convenient, sensitive, and specific assays for the noninvasive but reliable detection of chemokines for diagnosis and treatment guidance. While the rational design of cancer immunotherapies targeting disrupted epigenetic pathways related to chemokines may be a more realistic goal for pharmacological development, appropriate interpretation of the data requires an understanding of the spatial-temporal genetic variations and nongenetic heterogeneity in different microenvironments. Further proof-of-concept is warranted for translational studies of chemokine applications in precision medicine. Therefore, novel technology to be used in combination with single-cell-based 3-D imaging should be developed to allow more sensitive quantification of the complex chemokine interactome in health and diseases. In this context, processing bioinformatic analysis data with artificial intelligence (AI) systems has emerged as a major achievement in the era of chemokine biology research. Given these advances, it is time to further reveal the science behind chemokine biology to achieve precision medicine.

## Supplementary information

Sup Table 1

Sup Table 2

Sup Table 3
