# Supplementary material for: New genetic and epigenetic insights into the chemokine system: the latest discoveries aiding progression toward precision medicine
Source: Cell Mol Immunol. 2023 May 17;20(7):739–76. doi: 10.1038/s41423-023-01032-x (PMC10189238; doi:10.1038/s41423-023-01032-x)
Supplement: Supplementary file 1 — Sup Table 1 [file 41423_2023_1032_MOESM1_ESM.docx]

**Table S1.** A list of knockout mice deficient with the gene coding chemokine or chemokine receptor

| **Allele** | **Phenotype** | **Procedure** | **Parameter** | **Zygosity** | **Sex** | **Life Stage** | **P Value** |
| --- | --- | --- | --- | --- | --- | --- | --- |
| Ccr2^em1(IMPC)H^ | abnormal gait | Combined SHIRPA and Dysmorphology | Gait | hom | F | Early adult | 9.98E-5 |
|  | decreased lymphocyte cell number | Hematology | Lymphocyte differential count | hom | F | Late adult | 1.33E-5 |
|  | decreased monocyte cell number | Hematology | Monocyte differential count | hom | F | Late adult | 2.07E-11 |
|  |  |  |  | hom | M | Late adult | 4.11E-8 |
|  | decreased neutrophil cell number | Hematology | Monocyte cell count | hom | M | Late adult | 3.57E-8 |
|  | enlarged heart | Gross Pathology and Tissue Collection | Heart | hom | M | Late adult | 0.0 |
|  | increased eosinophil cell number | Hematology | Eosinophil differential count | hom | F | Late adult | 3.39E-21 |
|  |  |  | Eosinophil cell count | hom | F | Late adult | 9.58E-14 |
| Ccr3^em1(IMPC)Ccpcz^ | abnormal cornea morphology | Eye Morphology | Cornea | hom | F | Early adult | 8.87E-6 |
|  | abnormal heart morphology | Gross Pathology and Tissue Collection | Heart | hom | M | Early adult | 0.0 |
|  |  |  |  | hom | M | Early adult | 0.0 |
|  |  |  |  | hom | M | Early adult | 0.0 |
|  | abnormal spleen morphology | Gross Pathology and Tissue Collection | Spleen | hom | F | Early adult | 0.0 |
|  |  |  |  | hom | M | Early adult | 0.0 |
|  |  |  |  | hom | F | Early adult | 0.0 |
|  |  |  |  | hom | F | Early adult | 0.0 |
|  | abnormal thymus morphology | Gross Pathology and Tissue Collection | Thymus | hom | F | Early adult | 0.0 |
|  |  |  |  | hom | F | Early adult | 0.0 |
|  | enlarged heart | Gross Pathology and Tissue Collection | Heart | hom | M | Early adult | 0.0 |
|  |  |  |  | hom | M | Early adult | 0.0 |
|  |  |  |  | hom | M | Early adult | 0.0 |
|  | enlarged spleen | Gross Pathology and Tissue Collection | Spleen | hom | F | Early adult | 0.0 |
|  |  |  |  | hom | M | Early adult | 0.0 |
|  |  |  |  | hom | F | Early adult | 0.0 |
|  | enlarged thymus | Gross Pathology and Tissue Collection | Thymus | hom | F | Early adult | 0.0 |
|  |  |  |  | hom | F | Early adult | 0.0 |
| Ccr6^tm1.1(KOMP)Vlcg^ | abnormal spleen morphology | Gross Pathology and Tissue Collection | Spleen | hom | M | Early adult | 0 |
|  | decreased heart rate | Electrocardiogram (ECG) | HR | hom | M | Early adult | 2.64E-05 |
|  | prolonged RR interval | Electrocardiogram (ECG) | RR | hom | M | Early adult | 6.69E-05 |
| Ccr8^em1(IMPC)Mbp^ | abnormal eye morphology | Gross Pathology and Tissue Collection | Eye with optic nerve | hom | F | Early adult | 0.0 |
|  | blind uterus | Gross Pathology and Tissue Collection | Uterus | hom | F | Early adult | 0.0 |
| Cxcr1^tm1a(EUCOMM)Wtsi^ | decreased body length | Body Composition (DEXA lean/fat) | Body length | hom | M | Early adult | 3.82E-7 |
|  | decreased bone mineral content | Body Composition (DEXA lean/fat) | Bone Mineral Content (excluding skull) | hom | M | Early adult | 7.54E-5 |
|  | increased circulating insulin level | Insulin Blood Level | Insulin | hom | M | Early adult | 0.0 |
|  | increased fasting circulating glucose level | Intraperitoneal glucose tolerance test (IPGTT) | Fasted blood glucose concentration | hom | M | Early adult | 7.1E-5 |
|  | thrombocytopenia | Hematology | Platelet count | hom | U | Early adult | 7.74E-5 |
| Cxcr2^tm1a(EUCOMM)Wtsi^ | decreased B cell number | Bone marrow immunophenotyping | Total B cells - % of CD45+ | hom | U | Early adult | 0.0 |
|  |  | Whole blood peripheral blood leukocyte immunophenotyping | B cell percentage | hom | F |  | 0.0 |
|  | decreased CD8-positive, alpha-beta T cell number | Spleen Immunophenotyping | Total CD8+ T cells - % of ab T cells | hom | U | Early adult | 0.0 |
|  |  |  | Total CD8+ T cells - % of CD45+ | hom | U |  | 0.0 |
|  | decreased circulating fructosamine level | Clinical Chemistry | Fructosamine | hom | F | Early adult | 2.08E-12 |
|  | decreased circulating serum albumin level | Clinical Chemistry | Albumin | hom | F | Early adult | 1.4E-5 |
|  | decreased circulating thyroxine level | Clinical Chemistry | Thyroxine | hom | F | Early adult | 1.58E-5 |
|  | decreased immature B cell number | Bone marrow immunophenotyping | Immature B cells (Hardy fraction E) - % of CD45+ | hom | U | Early adult | 0.0 |
|  | decreased mature B cell number | Bone marrow immunophenotyping | Mature B cells (Hardy fraction F) - % of CD45+ | hom | U | Early adult | 0.0 |
|  | decreased mean corpuscular hemoglobin | Hematology | Mean corpuscular hemoglobin | hom | F | Early adult | 1.46E-8 |
|  | decreased pre-B cell number | Bone marrow immunophenotyping | Pre-B cells (Hardy fraction D) - % of CD45+ | hom | U | Early adult | 0.0 |
|  | decreased pre-pro B cell number | Bone marrow immunophenotyping | Pre-pro B cells (Hardy fraction A) - % of CD45+ | hom | U | Early adult | 0.0 |
|  | decreased T cell number | Bone marrow immunophenotyping | T cells - % of CD45+ | hom | U | Early adult | 0.0 |
|  | improved glucose tolerance | Intraperitoneal glucose tolerance test (IPGTT) | Area under glucose response curve | hom | U | Early adult | 6.97E-6 |
|  | increased anti-nuclear antigen antibody level | Anti-nuclear antibody assay | ANA classification | hom | F | Early adult | 0.0 |
|  |  |  |  | hom | M |  | 0.0 |
|  |  |  | ANA score | hom | F |  | 0.0 |
|  |  |  |  | hom | M |  | 0.0 |
|  | increased CD103-positive CD11b-low dendritic cell number | Spleen Immunophenotyping | CD103+ CD11b-low cDC - % of CD11b-low cDC | hom | U | Early adult | 0.0 |
|  | increased CD4-positive, alpha-beta memory T cell number | Whole blood peripheral blood leukocyte immunophenotyping | CD4+ CD44+ CD62L- alpha beta effector T cell number | hom | M | Early adult | 0.0 |
|  |  |  |  | hom | F |  | 0.0 |
|  |  |  |  | hom | M |  | 0.0 |
|  |  |  | CD8+ CD44+ CD62L- alpha beta effector T cell percentage | hom | M |  | 0.0 |
|  |  |  |  | hom | M |  | 0.0 |
|  |  |  |  | hom | F |  | 0.0 |
|  |  |  |  | hom | F |  | 0.0 |
|  | increased circulating calcium level | Clinical Chemistry | Calcium | hom | F | Early adult | 1.49E-7 |
|  | increased circulating creatinine level | Clinical Chemistry | Creatinine | het | F | Early adult | 4.25E-8 |
|  | increased circulating magnesium level | Clinical Chemistry | Magnesium | het | U | Early adult | 1.56E-5 |
|  | increased circulating total protein level | Clinical Chemistry | Total protein | hom | F | Early adult | 6.92E-13 |
|  |  |  |  | hom | M |  | 1.43E-11 |
|  | increased effector memory CD8-positive, alpha-beta T cell number | Spleen Immunophenotyping | Resting CD8+ T cells - % of CD45+ | hom | U | Early adult | 0.0 |
|  | increased effector memory T-helper cell number | Spleen Immunophenotyping | Effector CD4+ T helper cells - % of CD4+ T helper cells | hom | U | Early adult | 0.0 |
|  |  | Spleen Immunophenotyping | Resting CD4+ T helper cells - % of CD4+ T helper cells | hom | U |  | 0.0 |
|  | increased eosinophil cell number | Spleen Immunophenotyping | Eosinophils - % of CD45+ | hom | U | Early adult | 0.0 |
|  | increased gamma-delta T cell number | Whole blood peripheral blood leukocyte immunophenotyping | Gamma delta T cell percentage | hom | F | Early adult | 0.0 |
|  |  |  |  | hom | M |  | 0.0 |
|  |  |  | Gamma delta T cell number | hom | F |  | 0.0 |
|  |  |  |  | hom | M |  | 0.0 |
|  | increased granulocyte number | Bone marrow immunophenotyping | Granulocytes - % of CD45+ | hom | U | Early adult | 0.0 |
|  | increased KLRG1+ CD4 alpha beta T cell number | Whole blood peripheral blood leukocyte immunophenotyping | CD4+ KLRG1+ alpha beta T cell number | hom | F | Early adult | 0.0 |
|  |  | Mesenteric Lymph Node Immunophenotyping | KLRG1+ CD8+ T cells - % of CD45+ | hom | U |  | 0.0 |
|  | increased leukocyte cell number | Hematology | White blood cell count | hom | F | Early adult | 1.52E-13 |
|  | increased Ly6C high monocyte number | Whole blood peripheral blood leukocyte immunophenotyping | Ly6Chigh monocyte number | hom | F | Early adult | 0.0 |
|  | increased Ly6C low monocyte number | Whole blood peripheral blood leukocyte immunophenotyping | Ly6Clow monocyte number | hom | F | Early adult | 0.0 |
|  |  |  |  | hom | M |  | 0.0 |
|  | increased memory-marker CD4-positive NK T cell number | Spleen Immunophenotyping | Effector CD4+ NKT cells - % of CD45+ | hom | U | Early adult | 0.0 |
|  | increased memory-marker gamma-delta T cell number | Spleen Immunophenotyping | Resting gd T cells - % of gd T cells | hom | U | Early adult | 0.0 |
|  |  |  | Effector gd cells - % of CD45+ | hom | U | Early adult | 0.0 |
|  |  |  | Effector gd T cells - % of gd T cells | hom | U | Early adult | 0.0 |
|  |  | Mesenteric Lymph Node Immunophenotyping | Effector gd cells - % of CD45+ | hom | U | Early adult | 0.0 |
|  | increased memory-marker NK cell number | Spleen Immunophenotyping | Resting NK cells - % of CD45+ | hom | U | Early adult | 0.0 |
|  | increased monocyte cell number | Whole blood peripheral blood leukocyte immunophenotyping | Monocyte number | hom | M | Early adult | 0.0 |
|  |  |  |  | hom | F | Early adult | 0.0 |
|  |  |  |  | hom | F | Early adult | 0.0 |
|  |  |  | Neutrophil percentage | hom | F | Early adult | 0.0 |
|  |  |  |  | hom | F | Early adult | 0.0 |
|  |  |  |  | hom | M | Early adult | 0.0 |
|  | increased red blood cell distribution width | Hematology | Red blood cell distribution width | hom | U | Early adult | 2.07E-5 |
|  | thrombocytosis | Hematology | Platelet count | hom | F | Early adult | 4.2E-6 |
| Cxcr4^em1(IMPC)Mbp^ | abnormal eyelid morphology | Eye Morphology | Eyelid morphology | het | F | Early adult | 4.07E-5 |
|  | abnormal limb morphology | Gross Morphology Embryo E14.5-E15.5 | Limb morphology | hom | F | E15.5 | 0.0 |
|  |  |  |  | hom | M | E15.5 | 0.0 |
|  | abnormal placenta morphology | Gross Morphology Placenta E14.5-E15.5 | Placenta Morphology | hom | M | E15.5 | 0.0 |
|  |  |  |  | hom | F | E15.5 | 0.0 |
|  |  |  |  | het | M | E15.5 | 0.0 |
|  |  |  |  | het | F | E15.5 | 0.0 |
|  | abnormal placenta size | Gross Morphology Placenta E14.5-E15.5 | Placenta Size | het | M | E15.5 | 0.0 |
|  |  |  |  | het | F | E15.5 | 0.0 |
|  |  |  |  | hom | F | E15.5 | 0.0 |
|  |  |  |  | hom | M | E15.5 | 0.0 |
|  | edema | Gross Morphology Embryo E14.5-E15.5 | Edema | hom | F | E15.5 | 0.0 |
|  |  |  |  | hom | M | E15.5 | 0.0 |
|  | embryonic growth retardation | Gross Morphology Embryo E14.5-E15.5 | Delayed embryonic development | het | M | E15.5 | 0.0 |
|  |  |  |  | hom | F | E15.5 | 0.0 |
|  |  |  |  | hom | M | E15.5 | 0.0 |
|  |  |  |  | het | F | E15.5 | 0.0 |
|  | increased leukocyte cell number | Hematology | White blood cell count | het | M | Early adult | 2.47E-5 |
|  | increased lymphocyte cell number | Hematology | Lymphocyte cell count | het | U | Early adult | 4.4E-5 |
|  | increased monocyte cell number | Hematology | Monocyte cell count | het | M | Early adult | 8.72E-6 |
|  |  |  |  | het | F | Early adult | 4.42E-5 |
|  | microphthalmia | Gross Morphology Embryo E14.5-E15.5 | Microphthalmia | het | F | E15.5 | 0.0 |
|  |  |  |  | het | M | E15.5 | 0.0 |
|  | persistence of hyaloid vascular system | Eye Morphology | Persistence of hyaloid vascular system | het | M | Early adult | 2.58E-8 |
|  |  |  |  | het | F | Early adult | 3.99E-8 |
|  | preweaning lethality, complete penetrance | Viability Primary Screen | Homozygous females viability | hom | F | Early adult | 0.0 |
|  |  |  |  | hom | M | Early adult | 0.0 |
|  |  |  |  | hom | U | Early adult | 0.0 |
| Cx3cr1^em1(IMPC)Mbp^ | abnormal caudal vertebrae morphology | X-ray | Number of caudal vertebrae | hom | U | Early adult | 1.19E-6 |
|  | abnormal kidney morphology | Gross Pathology and Tissue Collection | Kidney | hom | M | Early adult | 0.0 |
|  |  |  |  | hom | F | Early adult | 0.0 |
|  | enlarged kidney | Gross Pathology and Tissue Collection | Kidney | hom | M | Early adult | 0.0 |
|  | small kidney | Gross Pathology and Tissue Collection | Kidney | hom | F | Early adult | 0.0 |
| Ackr3^tm2b(EUCOMM)Wtsi^ | decreased grip strength | Grip Strength | Forelimb grip strength measurement mean | het | M | Early adult | 2.09E-6 |
|  |  |  | Forelimb grip strength normalised against body weight | het | M | Early adult | 2.72E-5 |
|  | decreased total retina thickness | Eye Morphology | Right total retinal thickness | het | M | Early adult | 2.16E-5 |
|  |  |  | Left total retinal thickness | het | U | Early adult | 2.55E-5 |
|  | increased mean corpuscular hemoglobin concentration | Hematology | Mean cell hemoglobin concentration | het | M | Early adult | 1.6E-5 |
|  | preweaning lethality, complete penetrance | Viability Primary Screen | Viability Outcome | hom | U | Early adult | 0.0 |
|  | tremors | Combined SHIRPA and Dysmorphology | Tremor | het | U | Early adult | 7.93E-5 |
| Pitpnm3^em1(IMPC)Bay^ | abnormal bone structure | Body Composition (DEXA lean/fat) | Bone Area | hom | F | Early adult | 5.55E-13 |
|  | increased bone mineral content | Body Composition (DEXA lean/fat) | BMC/Body weight | hom | M | Early adult | 2.15E-5 |
|  |  |  |  | hom | F | Early adult | 1.99E-15 |
|  |  |  | Bone Mineral Content (excluding skull) | hom | F | Early adult | 7.48E-12 |
|  |  |  |  | hom | M | Early adult | 5.08E-5 |
|  | preweaning lethality, incomplete penetrance | Viability Primary Screen | Homozygous males viability | hom | M | Early adult | 0.0 |
|  |  |  | Homozygous animals viability | hom | U | Early adult | 0.0 |
| Ccl3^em1(IMPC)H^ | decreased circulating total protein level | Clinical Chemistry | Total protein | hom | F | Early adult | 3.05E-8 |
|  | decreased circulating serum albumin level | Clinical Chemistry | Albumin | hom | F | Early adult | 5.48E-5 |
| Ccl9^tm1(KOMP)Vlcg^ | enlarged heart | Gross Pathology and Tissue Collection | Heart | hom | M | Early adult | 0 |
|  | abnormal heart morphology | Gross Pathology and Tissue Collection | Heart | hom | M | Early adult | 0 |
|  | increased circulating HDL cholesterol level | Clinical Chemistry | HDL-cholesterol | hom | M | Early adult | 3.88E-06 |
|  | increased circulating cholesterol level | Clinical Chemistry | Total cholesterol | hom | M | Early adult | 4.83E-05 |
| Ccl17^em1(IMPC)H^ | increased spleen weight | Organ Weight | Spleen weight | hom | F | Early adult | 5.89E-19 |
| Ccl22^tm1a(EUCOMM)Wtsi^ | preweaning lethality, incomplete penetrance | Viability Primary Screen | Viability Outcome | hom | U | Early adult | 0.0 |
| Ccl25^em1(IMPC)H^ | enlarged spleen | Gross Pathology and Tissue Collection | Spleen | hom | F | Late adult | 0.0 |
|  | increased spleen weight | Organ Weight | Spleen weight | hom | F | Late adult | 2.7E-6 |
|  | increased grip strength | Grip Strength | Forelimb grip strength measurement mean | hom | U | Late adult | 2.85E-5 |
|  | decreased fasting circulating glucose level | Intraperitoneal glucose tolerance test (IPGTT) | Fasted blood glucose concentration | hom | F | Early adult | 7.6E-5 |
| Ccl26^tm1.1(KOMP)Vlcg^ | abnormal sleep behavior | Sleep Wake | Sleep daily percent | hom | F | Early adult | 1.28E-6 |
|  |  |  | Light sleep bout lengths standard deviation | hom | F | Early adult | 7.37E-5 |
|  | hyperactivity | Light-Dark Test | Time mobile dark side | hom | F | Early adult | 1.35E-5 |
|  |  |  | Side changes | hom | F | Early adult | 7.36E-5 |
|  | improved glucose tolerance | Intraperitoneal glucose tolerance test (IPGTT) | Area under glucose response curve | hom | M | Early adult | 4.88E-6 |
|  | increased mean corpuscular hemoglobin | Hematology | Mean corpuscular hemoglobin | hom | F | Early adult | 6.86E-6 |
| Ccl28^em1(IMPC)Ccpcz^ | abnormal eye morphology | Gross Pathology and Tissue Collection | Eye with optic nerve | hom | M | Early adult | 0.0 |
|  | abnormal heart morphology | Gross Pathology and Tissue Collection | Heart | hom | M | Early adult | 0.0 |
|  | abnormal liver morphology | Gross Pathology and Tissue Collection | Liver | hom | F | Early adult | 0.0 |
|  | abnormal skin morphology | Gross Pathology and Tissue Collection | Skin | hom | M | Early adult | 0.0 |
|  |  |  |  | hom | F | Early adult | 0.0 |
|  | abnormal spleen morphology | Gross Pathology and Tissue Collection | Spleen | hom | F | Early adult | 0.0 |
|  | abnormal thymus morphology | Gross Pathology and Tissue Collection | Thymus | hom | M | Early adult | 0.0 |
|  |  |  |  | hom | F | Early adult | 0.0 |
|  |  |  |  | hom | M | Early adult | 0.0 |
|  |  |  |  | hom | F | Early adult | 0.0 |
|  |  |  | Uterus | hom | F | Early adult | 0.0 |
|  | enlarged heart | Gross Pathology and Tissue Collection | Heart | hom | M | Early adult | 0.0 |
|  | enlarged spleen | Gross Pathology and Tissue Collection | Spleen | hom | F | Early adult | 0.0 |
|  | enlarged thymus | Gross Pathology and Tissue Collection | Thymus | hom | F | Early adult | 0.0 |
|  |  |  |  | hom | F | Early adult | 0.0 |
|  |  |  |  | hom | M | Early adult | 0.0 |
|  |  |  |  | hom | M | Early adult | 0.0 |
|  | hydrometra | Gross Pathology and Tissue Collection | Uterus | hom | F | Early adult | 0.0 |
|  | increased circulating alanine transaminase level | Clinical Chemistry | Alanine aminotransferase | hom | F | Early adult | 2.74E-7 |
|  | increased grip strength | Grip Strength | Forelimb and hindlimb grip strength normalised against body weight | hom | U | Early adult | 8.38E-5 |
| Cxcl1^em1(IMPC)Mbp^ | abnormal eye morphology | Gross Pathology and Tissue Collection | Eye with optic nerve | hom | F | Early adult | 0 |
|  |  |  |  | hom | F | Early adult | 0 |
|  | abnormal lymph node morphology | Gross Pathology and Tissue Collection | Lymph node | hom | M | Early adult | 0 |
|  | decreased bone mineral content | Body Composition (DEXA lean/fat) | Bone Mineral Content (excluding skull) | hom | F | Early adult | 5.90E-06 |
|  | enlarged lymph nodes | Gross Pathology and Tissue Collection | Lymph node | hom | M | Early adult | 0 |
|  | increased monocyte cell number | Hematology | Monocyte differential count | hom | F | Early adult | 1.48E-05 |
| Cxcl2^em1(IMPC)J^ | hyperactivity | Open Field - centre start | Whole arena resting time | hom | U | Early adult | 2.05E-5 |
|  |  | Light-Dark Test | Time mobile light side | hom | F | Early adult | 4.55E-5 |
| Cxcl5^em1(IMPC)KMPC^ | decreased startle reflex | Acoustic Startle and Pre-pulse Inhibition (PPI) | Response amplitude - S | hom | M | Early adult | 1.77E-06 |
|  | hyperactivity | Open Field | Whole arena average speed | hom | U | Early adult | 2.05E-05 |
|  |  |  | Distance travelled - total | hom | U | Early adult | 3.42E-05 |
|  | increased lean body mass | Body Composition (DEXA lean/fat) | Lean/Body weight | hom | U | Early adult | 7.81E-05 |
| Cxcl7^tm1.1(KOMP)Vlcg^ | decreased lymphocyte cell number | Hematology | Lymphocyte differential count | hom | M | Early adult | 1.46E-07 |
|  | increased neutrophil cell number | Hematology | Neutrophil differential count | hom | M | Early adult | 2.35E-10 |
|  |  |  | Neutrophil cell count | hom | U | Early adult | 2.12E-07 |
|  | increased spleen weight | Organ Weight | Spleen weight | hom | U | Early adult | 1.13E-06 |
| Cxcl9^tm1b(EUCOMM)Wtsi^ | decreased fasting circulating glucose level | Intraperitoneal glucose tolerance test (IPGTT) | Fasted blood glucose concentration | hom | M | Early adult | 1.22E-14 |
|  | decreased neutrophil cell number | Hematology | Neutrophil cell count | hom | M | Early adult | 1.45E-08 |
|  |  |  | Neutrophil differential count | hom | M | Early adult | 3.83E-06 |
|  | increased circulating cholesterol level | Clinical Chemistry | Total cholesterol | hom | M | Early adult | 7.72E-05 |
|  | increased circulating HDL cholesterol level | Clinical Chemistry | HDL-cholesterol | hom | M | Early adult | 2.17E-09 |
|  | increased lymphocyte cell number | Hematology | Lymphocyte differential count | hom | M | Early adult | 1.34E-05 |
| Cxcl12^tm1.1(KOMP)Vlcg^ | abnormal uterus morphology | Gross Pathology and Tissue Collection | Uterus | het | F | Early adult | 0.0 |
|  | enlarged uterus | Gross Pathology and Tissue Collection | Uterus | het | F | Early adult | 0.0 |
|  | hydrometra | Gross Pathology and Tissue Collection | Uterus | het | F | Early adult | 0.0 |
|  | increased mean platelet volume | Hematology | Mean platelet volume | het | M | Early adult | 9.26E-5 |
|  | preweaning lethality, complete penetrance | Viability Primary Screen | Viability Outcome | hom | U | Early adult | 0.0 |
|  | thrombocytopenia | Hematology | Platelet count | het | M | Early adult | 9.75E-6 |
| Cxcl13^em2(IMPC)H^ | increased basophil cell number | Hematology | Basophil cell count | hom | M | Early adult | 4.60E-07 |
|  | increased red blood cell distribution width | Hematology | Red blood cell distribution width | hom | M | Early adult | 7.20E-06 |
|  | increased lymphocyte cell number | Hematology | Lymphocyte cell count | hom | M | Early adult | 1.98E-05 |
|  | increased leukocyte cell number | Hematology | White blood cell count | hom | M | Early adult | 2.61E-05 |
| Cxcl14^em1(IMPC)H^ | decreased circulating serum albumin level | Clinical Chemistry | Albumin | hom | M | Early adult | 4.91E-05 |
|  | decreased circulating total protein level | Clinical Chemistry | Total protein | hom | U | Early adult | 6.95E-05 |
| Cxcl17^tm1b(EUCOMM)Wtsi^ | abnormal coat/hair pigmentation | Combined SHIRPA and Dysmorphology | Coat - color - back | hom | U | Early adult | 2.21E-7 |
|  |  |  | Coat - color - head | hom | U | Early adult | 2.21E-7 |
|  |  |  | Coat - color - abdomen | hom | U | Early adult | 3.29E-7 |
|  | abnormal heart morphology | Gross Pathology and Tissue Collection | Heart | hom | F | Early adult | 0.0 |
|  | abnormal kidney morphology | Gross Pathology and Tissue Collection | Kidney | hom | M | Early adult | 0.0 |
|  | abnormal skin morphology | Gross Pathology and Tissue Collection | Skin | hom | M | Early adult | 0.0 |
|  |  |  |  | hom | M | Early adult | 0.0 |
|  |  |  |  | hom | M | Early adult | 0.0 |
|  | abnormal uterus morphology | Gross Pathology and Tissue Collection | Uterus | hom | F | Early adult | 0.0 |
|  | enlarged heart | Gross Pathology and Tissue Collection | Heart | hom | F | Early adult | 0.0 |
|  | enlarged uterus | Gross Pathology and Tissue Collection | Uterus | hom | F | Early adult | 0.0 |
|  | increased circulating alanine transaminase level | Clinical Chemistry | Alanine aminotransferase | hom | F | Early adult | 2.11E-11 |
|  | increased circulating aspartate transaminase level | Clinical Chemistry | Aspartate aminotransferase | hom | F | Early adult | 1.39E-6 |
|  | increased mean platelet volume | Hematology | Mean platelet volume | hom | F | Early adult | 1.04E-5 |
| Cx3cl1^em3(IMPC)Ics^ | decreased exploration in new environment | Combined SHIRPA and Dysmorphology | Locomotor activity | hom | U | Early adult | 1.08E-6 |
|  | decreased locomotor activity | Open Field | Whole arena average speed | hom | U | Early adult | 3.59E-6 |
|  |  |  | Periphery average speed | hom | U | Early adult | 2.18E-5 |
|  | decreased vertical activity | Open Field | Number of rears - total | hom | M | Early adult | 2.73E-5 |
|  | increased respiratory quotient | Indirect Calorimetry | Respiratory Exchange Ratio | hom | U | Early adult | 4.39E-10 |

**Note:** The data were derived from the IMPC database (<https://www.mousephenotype.org/>). In the column of Sex, M: male, F: female, U: not considered.
