# Supplementary material for: New genetic and epigenetic insights into the chemokine system: the latest discoveries aiding progression toward precision medicine
Source: Cell Mol Immunol. 2023 May 17;20(7):739–76. doi: 10.1038/s41423-023-01032-x (PMC10189238; doi:10.1038/s41423-023-01032-x)
Supplement: Supplementary file 2 — Sup Table 2 [file 41423_2023_1032_MOESM2_ESM.docx]

**Table S2.** Phenotype, disease and trait annotations associated with genomic variants in chemokine ligands and receptors.

| Group | Phenotypes | Gene | Variant ID | Chr: bp | Alleles | Type | Reference |
| --- | --- | --- | --- | --- | --- | --- | --- |
| Brain_AD | Alzheimer disease | CCL22 | rs8044834 | 16:57290158 | C/G/T | SNP | PMID:17998437 |
|  | Cerebrospinal fluid levels of Alzheimer's disease-related proteins; Blood protein levels | CCR5 | rs6808835 | 3:46408373 | G/A/T | SNP | PMID:28240269, PMID:25340798 |
|  | Cerebrospinal fluid levels of Alzheimer's disease-related proteins; Blood protein levels | CCR3 | rs6808835 | 3:46408373 | G/A/T | SNP | PMID:28240269, PMID:25340798 |
|  | Cerebrospinal fluid levels of Alzheimer's disease-related proteins; Blood protein levels | CCRL2 | rs6808835 | 3:46408373 | G/A/T | SNP | PMID:28240269, PMID:25340798 |
|  | Cerebrospinal fluid p-tau levels in Alzheimer's disease dementia | CXCL14 | rs114090132 | 5:135810905 | C/G/T | SNP | PMID:29274321 |
|  | Cerebrospinal fluid t-tau levels | CXCL14 | rs71587588 | 5:135737280 | G/T | SNP | PMID:29274321 |
| Brain | Attention deficit hyperactivity disorder (ADHD) | CXCR4 | rs34442475 | 2:136306815 | T/A/C | SNP | PMID:32595297 |
|  | Brain morphology min-P | ACKR3 | rs11216435 | 11:117518217 | C/G/T | SNP | PMID:32665545 |
|  | Brain morphology min-P | ACKR4 | rs62005993 | 15:61480480 | C/T | SNP | PMID:32665545 |
|  | Brain morphology MOSTest | ACKR3 | rs4663669 | 2:236802433 | C/T | SNP | PMID:32665545 |
|  | Brain morphology MOSTest | ACKR4 | rs75537912 | 3:132572312 | A/G | SNP | PMID:32665545 |
|  | Carotid Artery Diseases; Erythrocytes; Uric Acid; Echocardiography; Alkaline Phosphatase | CCL1 | rs190038 | 17:34501507 | A/C/T | SNP | PMID:17903303, PMID:17903293, PMID:17903292, PMID:17903294, PMID:17903301 |
|  | Cognitive decline rate in late mild cognitive impairment | CX3CR1 | rs111231532 | 3:39135911 | C/T | SNP | PMID:26252872 |
|  | Cortical surface area MOSTest | ACKR4 | rs7900129 | 10:125990834 | A/G | SNP | PMID:32665545 |
|  | Cortical surface area MOSTest | ACKR3 | rs13235131 | 7:96583398 | G/T | SNP | PMID:32665545 |
|  | DUFFY BLOOD GROUP SYSTEM, FY(a-b-) PHENOTYPE | ACKR1 | rs587776507 | 1:159205719-159205738 | CCTGGCTGGCCTGTCCTGGC/CCTGGC | indel | MIM:613665 |
|  | DUFFY BLOOD GROUP SYSTEM, FY(bwk) PHENOTYPE | ACKR1 | rs34599082 | 1:159205704 | C/T | SNP | MIM:613665 |
|  | Hip | PITPNM3 | rs952849 | 17:6518129 | G/A/T | SNP | PMID:17903296 |
|  | Hippocampal volume | CXCL9 | rs139707122 | 4:75976951 | T/C/G | SNP | PMID:29274321 |
|  | Hippocampal volume | CXCL10 | rs139707122 | 4:75976951 | T/C/G | SNP | PMID:29274321 |
|  | Hippocampal volume | CXCL11 | rs139707122 | 4:75976951 | T/C/G | SNP | PMID:29274321 |
|  | Hippocampal volume | CXCL9 | rs79955867 | 4:76103243 | C/T | SNP | PMID:29274321 |
|  | Hippocampal volume | CXCL10 | rs79955867 | 4:76103243 | C/T | SNP | PMID:29274321 |
|  | Hippocampal volume | CXCL11 | rs79955867 | 4:76103243 | C/T | SNP | PMID:29274321 |
|  | Left superior temporal gyrus thickness (schizophrenia interaction) | CXCL12 | rs2802493 | 10:44058135 | T/C | SNP | PMID:26249676 |
|  | Psychosis proneness (hypomanic personality scale and revised social anhedonia scale); Psychosis proneness (perceptual aberration scale and revised social anhedonia scale); Psychosis proneness (revised physical anhedonia scale and revised social anhedonia scale); Psychosis proneness (revised social anhedonia scale) | CCR4 | rs11919880 | 3:32920559 | A/G/T | SNP | PMID:28525603 |
|  | Response to cognitive-behavioural therapy in major depressive disorder | CCL24 | rs13239350 | 7:75717941 | G/A | SNP | PMID:31123309 |
|  | Response to cognitive-behavioural therapy in major depressive disorder | CCL26 | rs13239350 | 7:75717941 | G/A | SNP | PMID:31123309 |
|  | Subcortical volume min-P | ACKR3 | rs1135999 | 16:15038105 | A/C/G | SNP | PMID:32665545 |
|  | Subcortical volume MOSTest | ACKR3 | rs62449208 | 7:31406487 | A/G | SNP | PMID:32665545 |
|  | Subcortical volume MOSTest | ACKR4 | rs55705857 | 8:129633446 | A/G | SNP | PMID:32665545 |
|  | Schizophrenia | CXCL12 | rs10900020 | 10:44331749 | A/T | SNP | PMID:23212062 |
|  | Social autistic-like traits | CCR6 | rs11575088 | 6:167143546 | C/A | SNP | PMID:24133439 |
| cancer | Basal cell carcinoma | CCR6 | rs4710154 | 6:166981146 | A/C/T | SNP | PMID:31174203 |
|  | Colorectal Cancer | CXCR4 | rs4954585 | 2:136240824 | C/G/T | SNP | PMID:29228715 |
|  | Follicular lymphoma | CXCR5 | rs4938573 | 11:118871133 | C/A/G/T | SNP | PMID:25279986 |
|  | Keratinocyte cancer MTAG | CCR6 | rs204295 | 6:167087074 | C/G/T | SNP | PMID:31174203 |
|  | Lung Cancer | XCL2 | rs77045810 | 1:168535779 | A/C | SNP | PMID:28604730 |
|  | Lung Cancer | CCR6 | rs17615336 | 6:167093246 | A/C/G | SNP | PMID:28604730 |
|  | Sensory peripheral neuropathy in microtubule targeting agent-treated breast cancer | CX3CL1 | rs11076190 | 16:57393024 | C/T | SNP | PMID:32562552 |
|  | squamous cell lung carcinoma | XCL2 | rs78663305 | 1:168528503 | A/G | SNP | PMID:28604730 |
| Cardiovascular system | Cardiac Troponin-T levels | CXCL13 | rs2201728 | 4:99242716 | G/A/T | SNP | PMID:23247143 |
|  | Carotid plaque burden | CCL26 | rs60454790 | 7:75789309 | C/T | SNP | PMID:28282560 |
|  | Coronary Artery Disease | CXCL12 | rs518594 | 10:44261659 | T/A/C | SNP | PMID:32469254 |
|  | Coronary heart disease; Coronary Artery Disease | CXCL12 | rs501120 | 10:44258419 | T/A/C | SNP | PMID:24262325, PMID:17634449 |
|  | Daytime sleep phenotypes | CCR8 | rs4276125 | 3:39243701 | C/A/G/T | SNP | PMID:27126917 |
|  | Daytime sleep phenotypes | CX3CR1 | rs4276125 | 3:39243701 | C/A/G/T | SNP | PMID:27126917 |
|  | Diastolic blood pressure | CXCL12 | rs2246438 | 10:44777631 | G/A | SNP | PMID:27841878 |
|  | Diastolic blood pressure | PITPNM3 | rs2009598 | 17:6567278 | G/A | SNP | PMID:30578418 |
|  | Diffusing capacity of carbon monoxide | CXCR1 | rs143014663 | 2:218175100 | G/A | SNP | PMID:30694715 |
|  | Echocardiography | CXCR4 | rs10496739 | 2:135994399 | G/A | SNP | PMID:17903301 |
|  | Echocardiography | CCR2 | rs10510751 | 3:46304534 | T/A/C/G | SNP | PMID:17903301 |
|  | Echocardiography | CCR3 | rs10510751 | 3:46304534 | T/A/C/G | SNP | PMID:17903301 |
|  | Electrocardiogram morphology amplitude at temporal datapoints | XCL1 | rs11580561 | 1:168629978 | C/T | SNP | PMID:32916098 |
|  | Heart Rate | CXCL9 | rs2276886 | 4:76007275 | C/A/T | SNP | PMID:17903306 |
|  | Myocardial infarction | CXCL12 | rs1704221 | 10:44215482 | G/A | SNP | PMID:33532862 |
|  | Myocardial infarction | CXCL12 | rs589655 | 10:44249867 | C/G | SNP | PMID:33532862 |
|  | Myocardial infarction | CCR1 | rs112720180 | 3:46152407 | A/G | SNP | PMID:33532862 |
|  | Myocardial infarction (early onset); Coronary heart disease | CXCL12 | rs1746048 | 10:44280376 | C/T | SNP | PMID:21378990, PMID:19198609 |
|  | Myocardial infarction; Coronary artery disease (myocardial infarction, percutaneous transluminal coronary angioplasty, coronary artery bypass grafting, angina or chromic ischemic heart disease); Coronary Artery Disease | CXCL12 | rs1870634 | 10:43985363 | T/G | SNP | PMID:26343387, PMID:28714975 |
|  | Narcolepsy with cataplexy | CCR1 | rs3181077 | 3:46209161 | C/T | SNP | PMID:25986216 |
|  | Pulse pressure; Systolic blood pressure | PITPNM3 | rs7226020 | 17:6570508 | T/C | SNP | PMID:27841878 |
|  | Takayasu arteritis | CCR7 | rs76139923 | 17:40563356 | C/T | SNP | PMID:33308445 |
| Eye | ClinVar: phenotype not specified; CONE-ROD DYSTROPHY 5; Retinitis pigmentosa | PITPNM3 | rs76024428 | 17:6468237 | C/G | SNP | MIM:608921 |
|  | ClinVar: phenotype not specified; OCULOMOTOR-ABDUCENS SYNKINESIS | ACKR3 | rs200582844 | 2:236581237 | G/A/T | SNP | MIM:610376 |
|  | Cognitive decline rate in late mild cognitive impairment | CX3CR1 | rs111231532 | 3:39135911 | C/T | SNP | PMID:26252872 |
|  | Disease progression in age-related macular degeneration | ACKR3 | rs56072732 | 2:236610853 | C/T | SNP | PMID:29346644 |
|  | Refractive error | CXCR5 | rs7947524 | 11:117800376 | C/A/T | SNP | PMID:32231278 |
|  | Spherical equivalent or myopia (age of diagnosis) | CCL4 | rs3213636 | 17:36147750 | A/C | SNP | PMID:29808027 |
|  | Upper eyelid sagging severity | CCL14 | rs7217473 | 17:35992028 | C/T | SNP | PMID:24869959 |
|  | Upper eyelid sagging severity | CCL15 | rs7217473 | 17:35992028 | C/T | SNP | PMID:24869959 |
| Liver | Primary biliary cholangitis | CXCR5 | rs77871618 | 11:118862915 | C/T | SNP | PMID:28425483 |
|  | Primary biliary cholangitis | CXCR5 | rs6421571 | 11:118873063 | T/A/C | SNP | PMID:26394269, PMID:21399635 |
|  | Primary biliary cholangitis | CCL20 | rs4973341 | 2:227795646 | C/G/T | SNP | PMID:26394269 |
|  | Primary biliary cholangitis | CCR6 | rs6456156 | 6:167108812 | T/A/C/G | SNP | PMID:28425483 |
| lung | Age at smoking initiation in chronic obstructive pulmonary disease | CXCL14 | rs10045413 | 5:135565466 | T/C | SNP | PMID:21685187 |
|  | Diffusing capacity of carbon monoxide | CXCL13 | rs12647197 | 4:77616391 | A/C/G | SNP | PMID:30694715 |
|  | Emphysema imaging phenotypes | XCL2 | rs72637224 | 1:168505772 | C/A/G/T | SNP | PMID:26030696 |
|  | Emphysema imaging phenotypes | XCL2 | rs75565482 | 1:168525756 | A/G | SNP | PMID:26030696 |
|  | FEV1 | PITPNM3 | rs4796334 | 17:6566473 | G/A | SNP | PMID:30804560 |
|  | Post bronchodilator FEV1/FVC ratio in COPD | CCL28 | rs78958340 | 5:43411501 | C/T | SNP | PMID:26634245 |
|  | PULMONARY FUNCTION | CCL23 | rs712046 | 17:36031260 | C/T | SNP | PMID:23932459 |
|  | Sarcoidosis | CCL24 | rs4728493 | 7:75817656 | C/A/T | SNP | PMID:32826979 |
| Bone | Bone mineral density (Ward's triangle area) | CCR3 | rs3091309 | 3:46261693 | A/C/G/T | SNP | PMID:27397699 |
|  | Heel bone mineral density | ACKR3 | rs1897468 | 2:236779553 | C/A/G/T | SNP | PMID:30598549 |
|  | Heel bone mineral density | CCR1 | rs4683184 | 3:46146215 | G/A/C | SNP | PMID:30598549 |
| Immune_Allergic disease | Eosinophil percentage of white cells; Eosinophil counts | CXCL12 | rs17482472 | 10:44364170 | G/A | SNP | PMID:32888494 |
|  | Allergy | CXCR5 | rs11217036 | 11:118805063 | C/G/T | SNP | PMID:27182965 |
|  | Allergic rhinitis | CXCR5 | rs28361986 | 11:118822452 | T/A | SNP | PMID:30013184 |
|  | Asthma (childhood onset); Asthma onset (childhood vs adult); Asthma; Asthma age of onset; Allergic disease (asthma, hay fever or eczema) | CXCR5 | rs12365699 | 11:118872577 | G/A | SNP | PMID:34103634, PMID:32296059, PMID:31959851, PMID:29083406, PMID:31036433, PMID:30929738 |
|  | Allergic disease asthma, hay fever and/or eczema multivariate analysis; Allergic disease asthma, hay fever and/or eczema age of onset | CXCR5 | rs4938576 | 11:118876060 | T/C/G | SNP | PMID:32603359 |
|  | Asthma | CCL22 | rs223819 | 16:57360950 | C/A/G/T | SNP | PMID:32296059 |
|  | Eosinophil counts; Eosinophil percentage of white cells | CX3CL1 | rs2239354 | 16:57385075 | G/A | SNP | PMID:32888494 |
|  | Asthma; Asthma (childhood onset) | CCR7 | rs9893132 | 17:40598769 | A/C/G/T | SNP | PMID:30929738 |
|  | Allergic disease asthma, hay fever and/or eczema age of onset; Allergic disease asthma, hay fever and/or eczema multivariate analysis | CCR7 | rs7216890 | 17:40600717 | G/A/C/T | SNP | PMID:32603359 |
|  | Asthma (childhood onset); Allergic rhinitis; Asthma; Eczema; Allergic disease (asthma, hay fever or eczema) | CCR7 | rs112401631 | 17:40608272 | T/A | SNP | PMID:29083406, PMID:31361310, PMID:31959851, PMID:30929738 |
|  | Allergic disease (asthma, hay fever or eczema) | CCR7 | rs11464691 | 17:40614390 | A/AA | indel | PMID:29083406 |
|  | Estimated glomerular filtration rate | CXCR4 | rs6708702 | 2:136316562 | G/A/T | SNP | PMID:31152163 |
|  | Basophil count | CXCR1 | rs16858768 | 2:218161975 | A/C | SNP | PMID:32888494 |
|  | Allergic disease asthma, hay fever and/or eczema age of onset; Asthma; Asthma (childhood onset); Allergic disease asthma, hay fever and/or eczema multivariate analysis; Asthma onset (childhood vs adult) | CCL20 | rs10187276 | 2:227805721 | T/A/C | SNP | PMID:32603359, PMID:30929738 |
|  | Asthma (childhood onset); Eating disorders (purging via substances) | CCL20 | rs10175070 | 2:227805859 | G/A/C/T | SNP | PMID:23568457, PMID:31036433 |
|  | Asthma | CCL20 | rs7423358 | 2:227840005 | T/C/G | SNP | PMID:31959851, PMID:32296059, PMID:34103634 |
|  | Allergic disease (asthma, hay fever or eczema) | CCL20 | rs13384448 | 2:227843146 | C/A/T | SNP | PMID:29083406 |
|  | Asthma | CCR4 | rs35570272 | 3:33006170 | G/T | SNP | PMID:31959851 |
|  | Eosinophil counts; Eosinophil percentage of white cells | CCR3 | rs138346219 | 3:46265822 | A/G | SNP | PMID:32888494 |
|  | Basophil percentage of granulocytes; Myeloid white cell count; Sum basophil neutrophil counts; Neutrophil percentage of white cells; Lymphocyte percentage of white cells; Neutrophil count; Granulocyte count | CXCL6 | rs62312418 | 4:73835795 | G/A | SNP | PMID:27863252 |
|  | Basophil percentage of white cells | CXCL6 | rs1957078 | 4:73838865 | C/A/G/T | SNP | PMID:32888494 |
|  | Eosinophil percentage of granulocytes; Neutrophil percentage of granulocytes | CXCL6 | rs13148728 | 4:73845409 | C/T | SNP | PMID:27863252 |
|  | Response to zileuton treatment in asthma (FEV1 change interaction) | CCR6 | rs3093009 | 6:167135989 | A/G | SNP | PMID:26031901 |
|  | Eosinophil counts; Eosinophil percentage of white cells; Sum eosinophil basophil counts; Eosinophil percentage of granulocytes | CCL24 | rs11465296 | 7:75812976 | C/T | SNP | PMID:27863252 |
|  | Eosinophil percentage of white cells; Eosinophil counts | CCL24 | rs13226583 | 7:75824834 | A/T | SNP | PMID:32888494 |
|  | Eosinophil counts; Eosinophil percentage of white cells | CCL24 | rs62477649 | 7:75827606 | G/A/T | SNP | PMID:32888494 |
| Autoimmune disease | Anti-drug antibodies in autoimmune disease time to event | CXCL12 | rs10508884 | 10:44325939 | C/T | SNP | PMID:33125391 |
|  | Sjogren's syndrome | CXCR5 | rs7119038 | 11:118867572 | G/A/C/T | SNP | PMID:24097067 |
|  | Behcet Syndrome | XCR1 | rs10510749 | 3:46138924 | C/T | SNP | PMID:20622878 |
|  | Behcet Syndrome | CCR1 | rs10510749 | 3:46138924 | C/T | SNP | PMID:20622878 |
|  | Behcet Syndrome | XCR1 | rs7631551 | 3:46144818 | C/A/T | SNP | PMID:20622878 |
|  | Behcet Syndrome | CCR1 | rs7631551 | 3:46144818 | C/A/T | SNP | PMID:20622878 |
|  | Behcet Syndrome | CCR2,CCR3 | rs9990343 | 3:46298321 | A/G | SNP | PMID:20622878 |
|  | Behcet's disease | CCR3 | rs7616215 | 3:46164194 | C/G/T | SNP | PMID:23291587 |
|  | Behcet's disease | CCR3 | rs2087726 | 3:46166818 | G/A | SNP | PMID:33393726 |
|  | Behcet's disease; CELIAC DISEASE | CCR1 | rs7616215 | 3:46164194 | C/G/T | SNP | PMID:22057235, PMID:23291587 |
|  | Arthritis (juvenile idiopathic) | CXCR4 | rs953387 | 2:136149600 | A/C/G/T | SNP | PMID:27005825 |
|  | Arthritis (juvenile idiopathic) | CCR1 | rs79815064 | 3:46236086 | A/G | SNP | PMID:33106285 |
|  | Arthritis (juvenile idiopathic) | CCR3 | rs79815064 | 3:46236086 | A/G | SNP | PMID:33106285 |
|  | Juvenile idiopathic arthritis (oligoarticular or rheumatoid factor-negative polyarticular) | CCR1 | rs79893749 | 3:46212159 | C/T | SNP | PMID:23603761 |
|  | Juvenile idiopathic arthritis (oligoarticular or rheumatoid factor-negative polyarticular) | CCR3 | rs79893749 | 3:46212159 | C/T | SNP | PMID:23603761 |
|  | Rheumatoid arthritis | CXCR5 | rs10790268 | 11:118858682 | A/G/T | SNP | PMID:24390342, PMID:30423114 |
|  | Rheumatoid arthritis | CCR2 | rs34030880 | 3:46363251 | A/T | SNP | PMID:32868391 |
|  | Rheumatoid arthritis | CXCL13 | rs117605225 | 4:77587043 | T/G | SNP | PMID:32723749 |
|  | Rheumatoid arthritis | CCR6 | rs3093024 | 6:167119305 | A/G/T | SNP | PMID:20453841 |
|  | Rheumatoid arthritis | CCR6 | rs1854853 | 6:167119574 | A/G | SNP | PMID:24782177 |
|  | Rheumatoid arthritis | CCR6 | rs3093023 | 6:167120802 | G/A/C/T | SNP | PMID:24782177, PMID:20453842 |
|  | Rheumatoid arthritis | CCR6 | rs3093019 | 6:167126167 | C/A/G | SNP | PMID:32723749 |
|  | Rheumatoid arthritis | CCL19 | rs11574914 | 9:34710341 | G/A | SNP | PMID:30423114, PMID:24390342 |
|  | Rheumatoid arthritis | CCL21 | rs11574914 | 9:34710341 | G/A | SNP | PMID:30423114, PMID:24390342 |
|  | Rheumatoid arthritis | CCL21 | rs951005 | 9:34743684 | G/A | SNP | PMID:20453842 |
|  | Rheumatoid arthritis (ACPA-positive); Rheumatoid arthritis; Chronic inflammatory diseases (ankylosing spondylitis, Crohn's disease, psoriasis, primary sclerosing cholangitis, ulcerative colitis) (pleiotropy); CELIAC DISEASE; Autoimmune traits pleiotropy | CCL21 | rs2812378 | 9:34710263 | G/A/C | SNP | PMID:24532676, PMID:23143596, PMID:30572963, PMID:26974007, PMID:18794853 |
|  | Rheumatoid arthritis; Rheumatoid arthritis (ACPA-positive) | CCR6 | rs59466457 | 6:167124266 | A/G | SNP | PMID:23143596 |
|  | Rheumatoid arthritis; Rheumatoid arthritis (ACPA-positive) | CCR6 | rs1571878 | 6:167127354 | C/G/T | SNP | PMID:24390342, PMID:24532676, PMID:23143596, PMID:30423114 |
|  | CELIAC DISEASE | CCR2 | rs9873580 | 3:159911578 | C/T | SNP | PMID:25920553 |
|  | CELIAC DISEASE | CCR3 | rs9873580 | 3:159911578 | C/T | SNP | PMID:25920553 |
|  | CELIAC DISEASE | CCR4 | rs13314993 | 3:32973977 | G/C/T | SNP | PMID:20190752 |
|  | CELIAC DISEASE | CCR4 | rs4678523 | 3:32996229 | T/C | SNP | PMID:22057235 |
|  | CELIAC DISEASE | CCR1 | rs13098911 | 3:46193709 | C/G/T | SNP | PMID:20190752 |
|  | CELIAC DISEASE | CCR2 | rs13098911 | 3:46193709 | C/G/T | SNP | PMID:20190752 |
|  | CELIAC DISEASE | CCR3 | rs13098911 | 3:46193709 | C/G/T | SNP | PMID:20190752 |
|  | CELIAC DISEASE | CCR5 | rs13098911 | 3:46193709 | C/G/T | SNP | PMID:20190752 |
|  | CELIAC DISEASE | CCR9 | rs13098911 | 3:46193709 | C/G/T | SNP | PMID:20190752 |
|  | CELIAC DISEASE | CCRL2 | rs13098911 | 3:46193709 | C/G/T | SNP | PMID:20190752 |
|  | CELIAC DISEASE | CCR1 | rs41432345 | 3:46208883 | T/C | SNP | PMID:30572963 |
|  | CELIAC DISEASE | CCR2 | rs13096142 | 3:46240253 | C/T | SNP | PMID:24999842 |
|  | CELIAC DISEASE | CCR3 | rs13096142 | 3:46240253 | C/T | SNP | PMID:24999842 |
|  | CELIAC DISEASE | CCR1,CCR3 | rs6441961 | 3:46310893 | T/C | SNP | PMID:18311140 |
|  | CELIAC DISEASE | CCR2 | rs2097282 | 3:46336534 | C/A/G/T | SNP | PMID:22057235 |
|  | CELIAC DISEASE | CCR3 | rs2097282 | 3:46336534 | C/A/G/T | SNP | PMID:22057235 |
|  | Celiac disease and Rheumatoid arthritis | CXCR5 | rs11217040 | 11:118809939 | C/A | SNP | PMID:26546613 |
|  | Celiac disease and Rheumatoid arthritis | CCR1 | rs67676925 | 3:46232768 | T/C | SNP | PMID:26546613 |
|  | Celiac disease and Rheumatoid arthritis | CCR3 | rs67676925 | 3:46232768 | T/C | SNP | PMID:26546613 |
|  | Crohn's disease | CCL1 | rs3091316 | 17:34266955 | G/A/C | SNP | PMID:22412388 |
|  | Crohn's disease | CCL8 | rs3091316 | 17:34266955 | G/A/C | SNP | PMID:22412388 |
|  | Crohn's disease | CCR6 | rs2149085 | 6:166957622 | T/A/C | SNP | PMID:23850713 |
|  | Crohn's disease | CCR6 | rs415890 | 6:166993145 | G/A/C/T | SNP | PMID:21102463 |
|  | Crohn's disease | CCR6 | rs2301436 | 6:167024500 | C/T | SNP | PMID:18587394, PMID:20570966 |
|  | Crohn's disease; Gut microbiota (functional units) | CCL2 | rs3091315 | 17:34266646 | A/G/T | SNP | PMID:21102463, PMID:27694959 |
|  | Crohn's disease; Gut microbiota (functional units); Inflammatory bowel disease | CCL2 | rs3091316 | 17:34266955 | G/A/C | SNP | PMID:28067908, PMID:22412388, PMID:27694959, PMID:23128233 |
|  | Crohn's disease; Inflammatory bowel disease | CCR6 | rs1819333 | 6:166960059 | T/C/G | SNP | PMID:27569725, PMID:23128233, PMID:28067908 |
|  | Multiple sclerosis | CXCR5 | rs9736016 | 11:118854185 | T/A | SNP | PMID:24076602 |
|  | Multiple sclerosis | CXCR5 | rs523604 | 11:118885029 | A/C/G | SNP | PMID:24076602 |
|  | Multiple sclerosis | CXCR4 | rs10191360 | 2:136127109 | T/C/G | SNP | PMID:31604244 |
|  | Multiple sclerosis | CCR4 | rs4679081 | 3:32971991 | T/A/C | SNP | PMID:24076602 |
|  | Multiple sclerosis; Electrocardiography | CXCR4 | rs882300 | 2:136218685 | T/A/C/G | SNP | PMID:19525953, PMID:17903306 |
|  | Multiple sclerosis; Inflammatory bowel disease | CXCR5 | rs630923 | 11:118883644 | C/A | SNP | PMID:23128233, PMID:21833088 |
|  | Systemic lupus erythematosus | CXCR5 | rs6589706 | 11:118877104 | A/G/T | SNP | PMID:32771030 |
|  | Systemic lupus erythematosus | CCL22 | rs223883 | 16:57354818 | G/A | SNP | PMID:28714469 |
|  | Systemic lupus erythematosus | CCL22 | rs669763 | 16:57356566 | C/G | SNP | PMID:33272962 |
|  | Systemic lupus erythematosus; Multiple sclerosis | CCL22 | rs223889 | 16:57358329 | T/C | SNP | PMID:24076602, PMID:28714469 |
|  | Autoimmune thyroid disease | CX3CR1 | rs11720041 | 3:39278509 | C/T | SNP | PMID:32581359 |
|  | Vitiligo | CXCR5 | rs638893 | 11:118827828 | G/A/C | SNP | PMID:22951725 |
|  | Vitiligo | CCR6 | rs2236313 | 6:166946901 | T/C | SNP | PMID:20526339 |
|  | Vitiligo | CCR6 | rs2247314 | 6:166956742 | T/C | SNP | PMID:27723757 |
| HIV | AIDS progression | CCRL2 | rs6441975 | 3:46386699 | A/C/G/T | SNP | PMID:21502085 |
|  | CCR5 POLYMORPHISM, AFRICAN-AMERICAN | CCR5 | rs1800944 | 3:46373906 | C/G/T | SNP | MIM:601373 |
|  | CCR5 POLYMORPHISM, ORIENTAL 1 | CCR5 | rs796065305 | 3:46373795-46373796 | CC/C | indel | MIM:601373 |
|  | CCR5 POLYMORPHISM, ORIENTAL 2 | CCR5 | rs1800452 | 3:46373570 | G/A | SNP | MIM:601373 |
|  | CCR5 PROMOTER POLYMORPHISM; HUMAN IMMUNODEFICIENCY VIRUS TYPE 1, SUSCEPTIBILITY TO; Acquired immunodeficiency syndrome, delayed progression to | CCR5 | rs1799987 | 3:46370444 | A/G | SNP | MIM:601373 |
|  | Human immunodeficiency virus type 1, increased perinatal transmission of | CCR5 | rs41469351 | 3:46370771 | C/T | SNP | MIM:601373 |
|  | HUMAN IMMUNODEFICIENCY VIRUS TYPE 1, SUSCEPTIBILITY TO | CXCL12 | rs387906400 | 10:44378102 | C/T | SNP | MIM:600835 |
|  | HUMAN IMMUNODEFICIENCY VIRUS TYPE 1, SUSCEPTIBILITY TO | CCR5 | rs1800940 | 3:46373082 | G/T | SNP | MIM:601373 |
|  | HUMAN IMMUNODEFICIENCY VIRUS TYPE 1, SUSCEPTIBILITY TO | CCR5 | rs1800560 | 3:46373205 | T/A | SNP | MIM:601373 |
|  | Setpoint viral load in HIV-1 infection; Pre-treatment viral load in HIV-1 infection | CCRL2 | rs1015164 | 3:46410189 | A/C/G/T | SNP | PMID:31219150, PMID:26553974 |
| HIV_TB | Resistance to Mycobacterium tuberculosis in HIV-positive individuals measured by a negative tuberculin skin test (continuous) | CXCL12 | rs7082209 | 10:44318888 | A/G | SNP | PMID:28628665 |
|  | Response to anti-retroviral therapy (ddI/d4T) in HIV-1 infection (Grade 3 peripheral neuropathy) | CXCL12 | rs266095 | 10:44368720 | C/T | SNP | PMID:24554482 |
| COVID-19 | Severe COVID-19 infection with respiratory failure analysis I; Severe COVID-19 infection with respiratory failure analysis II | CXCR6 | rs11385942 | 3:45834968-45834969 | AA/AAA | indel | PMID:32558485 |
|  | Severe COVID-19 infection with respiratory failure analysis II; Severe COVID-19 infection with respiratory failure analysis I | XCR1 | rs11385942 | 3:45834968-45834969 | AA/AAA | indel | PMID:32558485 |
|  | Severe COVID-19 infection with respiratory failure analysis II; Severe COVID-19 infection with respiratory failure analysis I | CCR9 | rs11385942 | 3:45834968-45834969 | AA/AAA | indel | PMID:32558485 |
| herpes virus | Human herpes virus 7 U14 antibody levels | CXCR5 | rs75438046 | 11:118896855 | G/A | SNP | PMID:33204752 |
| Inflammatory | Gut microbiota (functional units) | CCL11 | rs3091315 | 17:34266646 | A/G/T | SNP | PMID:27694959 |
|  | Gut microbiota (functional units); Crohn's disease | CCL7 | rs3091315 | 17:34266646 | A/G/T | SNP | PMID:27694959, PMID:21102463 |
|  | Gut microbiota (functional units); Crohn's disease; Inflammatory bowel disease | CCL11 | rs3091316 | 17:34266955 | G/A/C | SNP | PMID:28067908, PMID:27694959, PMID:23128233, PMID:22412388 |
|  | Inflammatory bowel disease | CXCR1 | rs2382817 | 2:218286495 | A/C/G/T | SNP | PMID:28067908, PMID:23128233 |
|  | Inflammatory bowel disease | CXCR2 | rs2382817 | 2:218286495 | A/C/G/T | SNP | PMID:28067908, PMID:23128233 |
|  | Inflammatory bowel disease | CXCL1 | rs2472649 | 4:73991991 | A/G/T | SNP | PMID:23128233 |
|  | Inflammatory bowel disease | CXCL2 | rs2472649 | 4:73991991 | A/G/T | SNP | PMID:23128233 |
|  | Inflammatory bowel disease | CXCL4 | rs2472649 | 4:73991991 | A/G/T | SNP | PMID:23128233 |
|  | Inflammatory bowel disease | CXCL4L1 | rs2472649 | 4:73991991 | A/G/T | SNP | PMID:23128233 |
|  | Inflammatory bowel disease | CXCL5 | rs2472649 | 4:73991991 | A/G/T | SNP | PMID:23128233 |
|  | Inflammatory bowel disease | CXCL6 | rs2472649 | 4:73991991 | A/G/T | SNP | PMID:23128233 |
|  | Inflammatory bowel disease; Crohn's disease | CCL13 | rs3091316 | 17:34266955 | G/A/C | SNP | PMID:23128233, PMID:22412388 |
|  | Inflammatory bowel disease; Crohn's disease; Gut microbiota (functional units) | CCL7 | rs3091316 | 17:34266955 | G/A/C | SNP | PMID:28067908, PMID:22412388, PMID:27694959 |
|  | Inflammatory bowel disease; Crohn's disease; Ulcerative colitis | CCL20 | rs7556897 | 2:227795396 | C/A/G/T | SNP | PMID:26192919 |
|  | Inflammatory bowel disease; Type 1 diabetes; Ulcerative colitis | CCR5 | rs113010081 | 3:46415921 | T/C | SNP | PMID:26192919, PMID:25751624, PMID:28067908 |
|  | Inflammatory bowel disease; Ulcerative colitis | CCR3 | rs113010081 | 3:46415921 | T/C | SNP | PMID:28067908, PMID:26192919 |
|  | Ulcerative colitis | CCR1 | rs907611 | 11:1852842 | G/A | SNP | PMID:26192919 |
|  | Ulcerative colitis | CCR2 | rs907611 | 11:1852842 | G/A | SNP | PMID:26192919 |
|  | Ulcerative colitis | CCR3 | rs907611 | 11:1852842 | G/A | SNP | PMID:26192919 |
|  | Ulcerative colitis | CCR5 | rs907611 | 11:1852842 | G/A | SNP | PMID:26192919 |
|  | Ulcerative colitis; Inflammatory bowel disease | CCR1 | rs113010081 | 3:46415921 | T/C | SNP | PMID:26192919, PMID:28067908 |
|  | Ulcerative colitis; Inflammatory bowel disease | CCR2 | rs113010081 | 3:46415921 | T/C | SNP | PMID:26192919, PMID:28067908 |
| Matabolism | Urate levels (BMI interaction) | CXCL5 | rs11729931 | 4:74007918 | T/A/C | SNP | PMID:25811787 |
|  | Waist circumference adjusted for body mass index | CXCL13 | rs191403645 | 4:77581902 | G/A/T | SNP | PMID:28552196 |
|  | Body Mass Index | CXCL12 | rs11239187 | 10:44637829 | A/C/G/T | SNP | PMID:25760438 |
|  | Body Mass Index | CCL3 | rs12150665 | 17:36558947 | T/C | SNP | PMID:26426971 |
|  | Body Mass Index | CCL3L1 | rs12150665 | 17:36558947 | T/C | SNP | PMID:26426971 |
|  | Body Mass Index | CCL4 | rs12150665 | 17:36558947 | T/C | SNP | PMID:26426971 |
|  | Body Mass Index | CCL4L2 | rs12150665 | 17:36558947 | T/C | SNP | PMID:26426971 |
|  | Body Mass Index | CCL3L3 | rs12150665 | 17:36558947 CHR_HSCHR17_7_CTG4:36559319 | T/C T/C | SNP | PMID:26426971 |
|  | Body Mass Index | CXCR4 | rs4988235 | 2:135851076 | G/A/C | SNP | PMID:26426971 |
|  | Body Mass Index; Blood Vessels | CXCL1 | rs2117721 | 4:73921168 | T/C/G | SNP | PMID:17903300, PMID:17903301 |
|  | Body Mass Index; Blood Vessels | CXCL7 | rs552582 | 4:73990141 | A/C/G/T | SNP | PMID:17903301, PMID:17903300 |
|  | Hypothyroidism | CCL2 | rs9901756 | 17:34137135 | A/C | SNP | PMID:22493691 |
|  | Idiopathic intracranial hypertension | CXCR1 | rs2234671 | 2:218164385 | C/G | SNP | PMID:29608535 |
|  | Initial pursuit acceleration | CCL16 | rs145146434 | 17:35965836 | G/A | SNP | PMID:29064472 |
|  | Medication use thyroid preparations | CCR8 | rs11714050 | 3:39315616 | A/G | SNP | PMID:31015401 |
|  | Metabolite levels | XCL1 | rs2207216 | 1:168724974 | C/A/G | SNP | PMID:23823483 |
|  | Metabolite levels | CCL13 | rs7217062 | 17:34418144 | A/C/G | SNP | PMID:23823483 |
|  | Metabolite levels | CCL14 | rs854666 | 17:36030108 | A/G/T | SNP | PMID:23823483 |
|  | Metabolite levels | CCL25 | rs7257658 | 19:8088439 | G/T | SNP | PMID:23823483 |
|  | Metabolite levels | CXCL4 | rs16850360 | 4:74006728 | A/G | SNP | PMID:22916037 |
|  | Metabolite levels | CXCL5 | rs16850360 | 4:74006728 | A/G | SNP | PMID:22916037 |
|  | Metabolite levels | CXCL7 | rs16850360 | 4:74006728 | A/G | SNP | PMID:22916037 |
|  | Metabolite levels | CXCL2 | rs526993 | 4:74125919 | G/A/T | SNP | PMID:23823483 |
|  | Metabolite levels | CCL19 | rs577442 | 9:34768598 | A/G/T | SNP | PMID:23823483 |
|  | Metabolite levels (X-11787) | CCL28 | rs11951515 | 5:43382756 | T/C | SNP | PMID:23934736 |
|  | Midgestational total 25-hydroxyvitamin D levels fetal genetic effect | ACKR1 | rs17666424 | 1:159217740 | G/A | SNP | PMID:32047095 |
|  | Obesity-related traits | CCR2 | rs12636651 | 3:46240900 | T/C | SNP | PMID:23251661 |
|  | Pancreatic beta-cell glucose sensitivity | CCR3 | rs71327027 | 3:46131718 | T/G | SNP | PMID:32944759 |
|  | Serum metabolite levels CMS; Serum metabolite levels | CXCL4 | rs11574452 | 4:73980944 | C/A | SNP | PMID:31636271 |
|  | Sphingolipid levels | CCL25 | rs12610250 | 19:8212829 | A/G | SNP | PMID:26068415 |
|  | Triglycerides | CCR6 | rs62436827 | 6:167135059 | A/G | SNP | PMID:24886709 |
|  | Trunk fat mass | CXCR5 | rs12363277 | 11:118893027 | T/C | SNP | PMID:28552196 |
|  | Type 1 diabetes | CCR7 | rs757411 | 17:40618898 | C/T | SNP | PMID:25751624 |
| smoking | Smoking initiation (ever regular vs never regular) | CXCL12 | rs117097449 | 10:44687273 | C/T | SNP | PMID:31089300 |
|  | Smoking status (ever vs never smokers) | CXCR4 | rs2222016 | 2:136385737 | C/A/G/T | SNP | PMID:30643258 |
| multiple | Age-related macular degeneration 12; HUMAN IMMUNODEFICIENCY VIRUS TYPE 1, RAPID PROGRESSION TO AIDS; Coronary artery disease, resistance to | CX3CR1 | rs3732379 | 3:39265765 | C/T | SNP | MIM:601470 |
|  | Chronic inflammatory diseases (ankylosing spondylitis, Crohn's disease, psoriasis, primary sclerosing cholangitis, ulcerative colitis) (pleiotropy) | CXCR2 | rs11676348 | 2:218145423 | C/G/T | SNP | PMID:26974007 |
|  | Chronic kidney disease; Hypertension; Myocardial infarction; Ischemic stroke; Coronary Artery Disease | CXCL8 | rs188378669 | 4:73741568 | G/A/T | SNP | PMID:30226566, PMID:29930801, PMID:30402224 |
|  | SPINA BIFIDA, SUSCEPTIBILITY TO; Coronary artery disease, development of, in hiv; Coronary artery disease, modifier of; MYCOBACTERIUM TUBERCULOSIS, SUSCEPTIBILITY TO | CCL2 | rs1024611 | 17:34252769 | A/G | SNP | MIM:158105 |
|  | Warts, hypogammaglobulinemia, infections, and myelokathexis | CXCR4 | rs104893625 | 2:136114901 | C/A | SNP | MIM:162643 |
|  | Warts, hypogammaglobulinemia, infections, and myelokathexis | CXCR4 | rs730880320 | 2:136114911-136114913 | AGA/A | indel | MIM:162643 |
|  | Warts, hypogammaglobulinemia, infections, and myelokathexis; none provided; ClinVar: phenotype not specified | CXCR4 | rs104893626 | 2:136114915 | G/C | SNP | MIM:162643 |
|  | WEST NILE VIRUS, SUSCEPTIBILITY TO; Resistance to hepatitis C virus; ClinVar: phenotype not specified; HUMAN IMMUNODEFICIENCY VIRUS TYPE 1, SUSCEPTIBILITY TO; Multiple sclerosis modifier of disease progression | CCR5 | rs333 | 3:46373453-46373487 | ACAGTCAGTATCAATTCTGGAAGAATTTCCAGACA/ACA | indel | MIM:601373 |
| other | CCL24 levels | CCL24 | rs6946822 | 7:75850130 | C/G/T | SNP | PMID:25147954 |
|  | CXCL5 levels | CXCL5 | rs425535 | 4:73998280 | T/A/C | SNP | PMID:25147954 |
|  | Interferon gamma-induced protein 10 levels | CXCL10 | rs192716315 | 4:75978023 | T/C | SNP | PMID:31217265 |
|  | Interferon gamma-induced protein 10 levels/CXCL10 | CXCL10 | rs141053179 | 4:76668758 | C/G | SNP | PMID:27989323 |
|  | QT interval (ambient particulate matter interaction) | CXCL12 | rs1619661 | 10:44237935 | T/C | SNP | PMID:28749367 |
|  | Smooth-surface caries | CXCR1 | rs1079204 | 2:218265791 | G/A/C | SNP | PMID:23470693 |
|  | Smooth-surface caries | CXCR2 | rs1079204 | 2:218265791 | G/A/C | SNP | PMID:23470693 |
|  | Telomere length | CXCR4 | rs4452212 | 2:136258421 | G/A | SNP | PMID:20421499 |
|  | Tonsillectomy | CXCL13 | rs7685785 | 4:77519271 | C/T | SNP | PMID:27182965, PMID:28928442 |

Note: The data were extracted from https://asia.ensembl.org/.
