# Supplementary material for: New genetic and epigenetic insights into the chemokine system: the latest discoveries aiding progression toward precision medicine
Source: Cell Mol Immunol. 2023 May 17;20(7):739–76. doi: 10.1038/s41423-023-01032-x (PMC10189238; doi:10.1038/s41423-023-01032-x)
Supplement: Supplementary file 3 — Sup Table 3 [file 41423_2023_1032_MOESM3_ESM.docx]

**Table S3.** Phenotypic trait-associated differentially methylated CpGs occluding chemokine ligands and receptors with health condition and diseases

| Group | Phenotypes/Trait | Probe ID | Position | Gene | P value | PMID |
| --- | --- | --- | --- | --- | --- | --- |
| Aging | aging | cg04131610 | 3:46411447 | *CCR5* | 1.63E-08 | 30626398 |
|  | aging | cg01506627 | 3:46449289 | *CCRL2* | 1.46E-08 | 30626398 |
|  | aging | cg16021018 | 3:46449313 | *CCRL2* | 1.33E-08 | 30626398 |
|  | aging | cg20742784 | 5:43397451 | ***CCL28*** | 6.88E-11 | 30626398 |
|  | aging | cg08708644 | 5:43397467 | ***CCL28*** | 5.95E-09 | 30626398 |
|  | aging | cg22724765 | 5:43397572 | ***CCL28*** | 1.08E-10 | 30626398 |
|  | aging | cg00182712 | 5:43397617 | ***CCL28*** | 3.84E-09 | 30626398 |
|  | aging | cg26088632 | 5:43401961 | *CCL28* | 1.59E-08 | 30626398 |
|  | aging | cg15195412 | 16:57406955 | ***CX3CL1*** | 6.67E-09 | 30626398 |
|  | aging | cg26596307 | 16:57411505 | ***CX3CL1*** | 5.33E-10 | 30626398 |
|  | aging | cg23663547 | 17:38710320 | ***CCR7*** | 3.46E-09 | 30626398 |
|  | aging | cg26716902 | 17:40835920 | ***CCR10*** | 1.07E-09 | 30626398 |
|  | aging | cg09776463 | 17:40836087 | ***CCR10*** | 1.06E-09 | 30626398 |
|  | aging | cg27616541 | 17:40837288 | ***CCR10*** | 1.53E-09 | 30626398 |
|  | aging | cg04167480 | 17:6433689 | ***PITPNM3*** | 9.25E-09 | 30626398 |
|  | aging | cg11830644 | 17:6443322 | ***PITPNM3*** | 6.60E-09 | 30626398 |
| Cancer | head and neck squamous cell carcinoma (HNSCC) | cg13519373 | 2:219030898 | *CXCR1* | 1.22E-08 | 32961999 |
|  | head and neck squamous cell carcinoma (HNSCC) | cg13519373 | 2:219030898 | ***CXCR1*** | 1.76E-10 | 32961999 |
|  | head and neck squamous cell carcinoma (HNSCC) | cg05094429 | 6:167536184 | *CCR6* | 1.08E-07 | 32961999 |
|  | chemotherapy for breast cancer | cg11303839 | 7:75405967 | ***CCL26*** | 3.79E-13 | 30867049 |
|  | chemotherapy for breast cancer | cg23663547 | 17:38710320 | ***CCR7*** | 7.61E-10 | 30867049 |
|  | noninvasive sporadic breast cancer | cg22163463 | 17:6458640 | *PITPNM3* | 0.0489 | 33145876 |
| Cardiovascular | diastolic blood pressure | cg09425228 | 2:228678005 | *CCL20* | 3.07E-05 | 31999706 |
|  | coronary artery disease (CAD) | cg11267527 | 10:44881934 | *CXCL12* | 0.00984 | 30844764 |
| Genetic | ancestry | cg22022041 | 3:45928137 | *CCR9* | 9.51E-05 | 33108347 |
| Perinatal | cell-specific characterization of the placental methylome | cg19225688 | 2:218990043 | ***CXCR2*** | 4.26E-15 | 33407091 |
|  | gestational age | cg03341377 | 3:39309355 | ***CX3CR1*** | 2.75E-09 | 30966880 |
|  | maternal hypertensive disorders in pregnancy | cg03341377 | 3:39309355 | *CX3CR1* | 6.63E-06 | 31230546 |
|  | maternal hypertensive disorders in pregnancy | cg24310395 | 3:39309435 | *CX3CR1* | 1.20E-06 | 31230546 |
|  | gestational age | cg03928384 | 3:46395191 | ***CCR2*** | 7.56E-12 | 30966880 |
|  | maternal hypertensive disorders in pregnancy | cg03928384 | 3:46395191 | *CCR2* | 6.56E-05 | 31230546 |
|  | gestational age | cg05670596 | 3:46448496 | ***CCRL2*** | 3.76E-10 | 30966880 |
|  | polychlorinated biphenyls (PCBs) exposure | cg16055869 | 4:74864612 | *CXCL5* | 5.46E-06 | 30776747 |
|  | gestational age | cg22724765 | 5:43397572 | *CCL28* | 8.09E-08 | 30966880 |
|  | birth weight | cg11303839 | 7:75405967 | ***CCL26*** | 6.38E-21 | 31015461 |
|  | gestational age | cg18728264 | 11:118766491 | ***CXCR5*** | 2.24E-13 | 30966880 |
|  | maternal hypertensive disorders in pregnancy | cg08298591 | 16:57391829 | *CCL22* | 3.75E-05 | 31230546 |
|  | maternal hypertensive disorders in pregnancy | cg15195412 | 16:57406955 | *CX3CL1* | 4.79E-07 | 31230546 |
|  | gestational age | cg02483931 | 17:34202461 | ***CCL5*** | 1.87E-11 | 30966880 |
|  | birth weight | cg22647738 | 17:34304462 | ***CCL16*** | 1.70E-10 | 31015461 |
|  | birth weight | cg23663547 | 17:38710320 | *CCR7* | 1.41E-08 | 31015461 |
|  | gestational age | cg23663547 | 17:38710320 | *CCR7* | 2.54E-08 | 30966880 |
|  | cell-specific characterization of the placental methylome | cg16047279 | 17:38717242 | ***CCR7*** | 3.89E-22 | 33407091 |
|  | cell-specific characterization of the placental methylome | cg07248223 | 17:38717275 | ***CCR7*** | 1.49E-15 | 33407091 |
|  | prenatal bisphenol A (BPA) exposure | cg24389239 | 17:6365559 | *PITPNM3* | 1.48E-05 | 31451752 |
|  | maternal hemoglobin levels in pregnancy | cg06928695 | 17:6384119 | *PITPNM3* | 2.73E-06 | 33331245 |
| Immune disease | systemic lupus erythematosus (SLE) | cg23374992 | 2:136872067 | ***CXCR4*** | 8.27E-98 | 31428085 |
|  | systemic lupus erythematosus (SLE) | cg12595667 | 2:136872094 | ***CXCR4*** | 5.38E-68 | 31428085 |
|  | systemic lupus erythematosus (SLE) | cg25941354 | 2:218989983 | ***CXCR2*** | 3.97E-78 | 31428085 |
|  | diffuse cutaneous systemic sclerosis | cg06547715 | 2:218990976 | *CXCR2* | 4.35E-05 | 30947741 |
|  | systemic lupus erythematosus (SLE) | cg06547715 | 2:218990976 | ***CXCR2*** | 2.95E-54 | 31428085 |
|  | systemic lupus erythematosus (SLE) | cg15768138 | 2:219030752 | ***CXCR1*** | 7.19E-48 | 31428085 |
|  | Behcets disease | cg04498110 | 3:39306129 | *CX3CR1* | 2.28E-06 | 30863869 |
|  | autoimmune diseases | cg03341377 | 3:39309355 | ***CX3CR1*** | 1.12E-11 | 31024609 |
|  | systemic lupus erythematosus (SLE) | cg03341377 | 3:39309355 | ***CX3CR1*** | 3.13E-77 | 31428085 |
|  | autoimmune diseases | cg24310395 | 3:39309435 | ***CX3CR1*** | 2.20E-11 | 31024609 |
|  | systemic lupus erythematosus (SLE) | cg24310395 | 3:39309435 | ***CX3CR1*** | 3.88E-98 | 31428085 |
|  | systemic lupus erythematosus (SLE) | cg08450017 | 3:45984838 | ***CXCR6*** | 1.44E-130 | 31428085 |
|  | systemic lupus erythematosus (SLE) | cg01178899 | 3:45985168 | ***CXCR6*** | 5.97E-60 | 31428085 |
|  | Crohns disease (CD)-IBD | cg10499974 | 3:46244099 | *CCR3* | 5.70E-06 | 30779925 |
|  | perinatally acquired HIV | cg10499974 | 3:46244099 | *CCR3* | 0.000352 | 31324826 |
|  | systemic lupus erythematosus (SLE) | cg10499974 | 3:46244099 | ***CCR3*** | 3.60E-30 | 31428085 |
|  | perinatally acquired HIV | cg14312439 | 3:46283902 | *CCR3* | 0.0201 | 31324826 |
|  | systemic lupus erythematosus (SLE) | cg23350385 | 3:46448134 | ***CCRL2*** | 3.51E-63 | 31428085 |
|  | Behcets disease | cg08679238 | 3:46449100 | *CCRL2* | 3.49E-06 | 30863869 |
|  | systemic lupus erythematosus (SLE) | cg16021018 | 3:46449313 | ***CCRL2*** | 8.83E-57 | 31428085 |
|  | systemic lupus erythematosus (SLE) | cg18356190 | 4:74964260 | ***CXCL2*** | 1.01E-53 | 31428085 |
|  | systemic lupus erythematosus (SLE) | cg14192130 | 6:167535764 | ***CCR6*** | 5.54E-104 | 31428085 |
|  | systemic lupus erythematosus (SLE) | cg11303839 | 7:75405967 | ***CCL26*** | 3.28E-19 | 31428085 |
|  | limited cutaneous systemic sclerosis | cg07269146 | 9:34710657 | *CCL21* | 9.92E-05 | 30947741 |
|  | systemic lupus erythematosus (SLE) | cg04537602 | 11:118763859 | ***CXCR5*** | 3.42E-53 | 31428085 |
|  | systemic lupus erythematosus (SLE) | cg13298528 | 11:118763863 | ***CXCR5*** | 7.24E-56 | 31428085 |
|  | Crohns disease (CD) | cg08298591 | 16:57391829 | *CCL22* | 3.80E-05 | 30779925 |
|  | systemic lupus erythematosus (SLE) | cg01636591 | 17:32646156 | ***CCL8*** | 2.72E-62 | 31428085 |
|  | systemic lupus erythematosus (SLE) | cg17118262 | 17:32690569 | ***CCL1*** | 1.41E-56 | 31428085 |
|  | systemic lupus erythematosus (SLE) | cg10315334 | 17:34207332 | ***CCL5*** | 3.92E-60 | 31428085 |
|  | perinatally acquired HIV | cg22647738 | 17:34304462 | ***CCL16*** | 8.18E-09 | 31324826 |
|  | Autoimmue_ankylosing spondylitis | cg04850148 | 17:34539744 | *CCL4L1* | 1.50E-08 | 31128893 |
|  | Autoimmue_ankylosing spondylitis | cg04850148 | 17:34539744 | *CCL4L1* | 1.85E-05 | 31128893 |
|  | systemic lupus erythematosus (SLE) | cg26960939 | 17:38717206 | ***CCR7*** | 1.10E-44 | 31428085 |
|  | systemic lupus erythematosus (SLE) | cg07248223 | 17:38717275 | ***CCR7*** | 2.10E-33 | 31428085 |
|  | systemic lupus erythematosus (SLE) | cg06426114 | 17:6355097 | ***PITPNM3*** | 1.33E-25 | 31428085 |
|  | opioid dependence (OD) | cg21274724 | 17:6386543 | *PITPNM3* | 1.07E-05 | 30874594 |
| Metabolism | bariatric surgery | cg13854983 | 2:136875315 | ***CXCR4*** | 1.27E-11 | 31959221 |
|  | bariatric surgery | cg03101422 | 2:136876737 | ***CXCR4*** | 8.01E-11 | 31959221 |
|  | end-stage kidney disease attributed to¬†diabetic kidney disease | cg24310395 | 3:39309435 | ***CX3CR1*** | 2.91E-14 | 33933144 |
|  | end-stage kidney disease attributed to¬†diabetic kidney disease | cg05717123 | 3:39314701 | ***CX3CR1*** | 4.37E-15 | 33933144 |
|  | differentiation of skeletal muscle | cg13070763 | 3:46448963 | *CCRL2* | 1.80E-08 | 32958812 |
|  | body mass index (BMI) | cg04004578 | 4:74904790 | *CXCL3* | 9.03E-05 | 31910897 |
|  | bariatric surgery | cg18995088 | 5:134914734 | ***CXCL14*** | 6.23E-10 | 31959221 |
|  | end-stage kidney disease attributed to¬†diabetic kidney disease | cg04269510 | 6:167527544 | ***CCR6*** | 4.28E-13 | 33933144 |
|  | end-stage kidney disease attributed to¬†diabetic kidney disease | cg14192130 | 6:167535764 | ***CCR6*** | 1.56E-16 | 33933144 |
|  | end-stage kidney disease attributed to¬†diabetic kidney disease | cg05824215 | 6:167536046 | ***CCR6*** | 1.74E-14 | 33933144 |
|  | end-stage kidney disease attributed to¬†diabetic kidney disease | cg05094429 | 6:167536184 | ***CCR6*** | 1.52E-15 | 33933144 |
|  | bariatric surgery | cg25721625 | 10:44880236 | ***CXCL12*** | 4.33E-12 | 31959221 |
|  | end-stage kidney disease attributed to¬†diabetic kidney disease | cg16280667 | 11:118754593 | ***CXCR5*** | 1.38E-13 | 33933144 |
|  | end-stage kidney disease attributed to¬†diabetic kidney disease | cg12839838 | 11:118755586 | ***CXCR5*** | 5.88E-13 | 33933144 |
|  | end-stage kidney disease attributed to¬†diabetic kidney disease | cg26026450 | 11:118756203 | ***CXCR5*** | 1.37E-13 | 33933144 |
|  | bariatric surgery | cg01305736 | 17:40831891 | ***CCR10*** | 4.97E-15 | 31959221 |
|  | differentiation of skeletal muscle | cg17259086 | 17:4640603 | *CXCL16* | 2.21E-07 | 32958812 |
| Neurological and mental | Gulf War Illness | cg12595667 | 2:136872094 | *CXCR4* | 0.001013 | 30920300 |
|  | oculo-auriculo-vertebral-spectrum | cg02902079 | 2:136874716 | ***CXCR4*** | 2.71E-12 | 33530447 |
|  | Gulf War Illness | cg09075476 | 2:228680054 | *CCL20* | 0.001212 | 30920300 |
|  | Gulf War Illness | cg17642041 | 3:45928007 | *CCR9* | 0.000163 | 30920300 |
|  | Gulf War Illness | cg06997537 | 3:45928018 | *CCR9* | 5.20E-05 | 30920300 |
|  | alcohol consumption | cg13468041 | 4:74902951 | *CXCL3* | 0.002 | 30938765 |
|  | Gulf War Illness | cg17023631 | 4:76957478 | *CXCL11* | 0.000221 | 30920300 |
|  | Gulf War Illness | cg12125606 | 4:78500969 | *CXCL13* | 0.000616 | 30920300 |
|  | circadian rhythm | cg16296902 | 5:43396802 | *CCL28* | 5.99E-03 | 30606238 |
|  | alcohol consumption | cg18728264 | 11:118766491 | *CXCR5* | 2.94E-08 | 31789449 |
|  | opioid dependence (OD) | cg26799806 | 16:57437848 | *CCL17* | 1.43E-06 | 30874594 |
|  | Gulf War Illness | cg23663547 | 17:38710320 | *CCR7* | 0.000122 | 30920300 |
|  | Gulf War Illness | cg06864083 | 17:40832319 | *CCR10* | 0.000908 | 30920300 |
|  | Gulf War Illness | cg05043557 | 17:6460360 | *PITPNM3* | 0.00093 | 30920300 |

**Note:** In the phenotypes/trait, RE: the correlation with positive, BLAC: negative, or BLUE: no indication (NA). CAD: coronary artery disease; CD: Crohns disease; HDP: hypertensive disorders in pregnancy; HNSCC: head and neck squamous cell carcinoma; SLE: systemic lupus erythematosus.
